# Systematic assessment of prognostic gene signatures for breast cancer shows distinct influence of time and ER status

**DOI:** 10.1186/1471-2407-14-211

**Published:** 2014-03-19

**Authors:** Xi Zhao, Einar Andreas Rødland, Therese Sørlie, Hans Kristian Moen Vollan, Hege G Russnes, Vessela N Kristensen, Ole Christian Lingjærde, Anne-Lise Børresen-Dale

**Affiliations:** 1 Department of Genetics, Institute for Cancer Research, Oslo University Hospital, The Norwegian Radium Hospital, Montebello 0310 Oslo, Norway; 2 The K.G. Jebsen Center for Breast Cancer Research, Institute for Clinical Medicine, Faculty of Medicine, University of Oslo, Oslo, Norway; 3 Department of Radiology, Center for Cancer Systems Biology, School of Medicine, Stanford University, Stanford, USA; 4 Department of Informatics, Biomedical Research Group, Faculty of Mathematics and Natural Sciences, University of Oslo, Oslo, Norway; 5 Center for Cancer Biomedicine, University of Oslo, Oslo, Norway; 6 Department of Oncology, Division of Cancer Medicine, Surgery and Transplantation, Oslo University Hospital Radiumhospitalet, Montebello 0310 Oslo, Norway; 7 Department of Pathology, Institute for Cancer Research, Oslo University Hospital, The Norwegian Radium Hospital, Montebello 0310 Oslo, Norway

**Keywords:** Breast cancer, Prognosis, Gene signature, Long-term survival prediction, Molecular subtype

## Abstract

**Background:**

The aim was to assess and compare prognostic power of nine breast cancer gene signatures (Intrinsic, PAM50, 70-gene, 76-gene, Genomic-Grade-Index, 21-gene-Recurrence-Score, EndoPredict, Wound-Response and Hypoxia) in relation to ER status and follow-up time.

**Methods:**

A gene expression dataset from 947 breast tumors was used to evaluate the signatures for prediction of Distant Metastasis Free Survival (DMFS). A total of 912 patients had available DMFS status. The recently published METABRIC cohort was used as an additional validation set.

**Results:**

Survival predictions were fairly concordant across most signatures. Prognostic power declined with follow-up time. During the first 5 years of followup, all signatures except for Hypoxia were predictive for DMFS in ER-positive disease, and 76-gene, Hypoxia and Wound-Response were prognostic in ER-negative disease. After 5 years, the signatures had little prognostic power. Gene signatures provide significant prognostic information beyond tumor size, node status and histological grade.

**Conclusions:**

Generally, these signatures performed better for ER-positive disease, indicating that risk within each ER stratum is driven by distinct underlying biology. Most of the signatures were strong risk predictors for DMFS during the first 5 years of follow-up. Combining gene signatures with histological grade or tumor size, could improve the prognostic power, perhaps also of long-term survival.

## Background

Breast cancer is a heterogeneous disease. Tumors with similar clinico-pathological characteristics can have markedly different clinical courses. Gene signatures developed from genome-wide expression profiling of breast cancer have been shown to provide overlapping clinico-pathological classifications, and more importantly, to add prognostic accuracy and could potentially guide clinical decisions [1-9].

Despite the fact that a large number of expression-based gene signatures have been developed for breast cancer for prognostic and predictive purpose, the clinical value of these signatures has not been confirmed in prospective studies and the consequence for therapy remains unclear. The 10-year results of ongoing clinical trials [10,11] for testing the clinical benefit of gene signatures [4,12] will not be available until 2020. Outcome prediction by gene signatures has been criticized for being inaccurate [13]. Most studies evaluating various signatures [14-18] have been carried out on relatively small scales. Compatibility between the signatures and the targeted cohorts with respect to biological and pathological characteristics (Additional file 1: Table S1) is often ignored [16]. Use of validation sets not completely independent of the original training sets may have influenced the results leading to biased interpretation [14]. Furthermore, computing signature scores from inadequately transformed data may have resulted in unreliable or spurious results [19,20]. It therefore remains desirable to evaluate existing signatures in greater scrutiny on a reasonably sized and representative breast cancer cohort and pinpoint important specifications for more effective use of molecular-based tests in clinical settings.

With this in mind, we investigated nine signatures that have received great interest and been validated in multiple studies. These are Intrinsic signature [1-3,21] and PAM50 [9] for classifying breast tumors into five subtypes: luminal A (LumA), luminal B (LumB), HER2-enriched, basal-like, and normal-like; 70-gene profile or MammaPrint® (Agendia, Amsterdam, The Netherlands) [4,5,22-24] for predicting metastasis free survival over a five-year period; 76-gene signature [6,25,26] for predicting distant metastasis within five years for lymph-node-negative breast cancers; genomic grade index (GGI) [7,27] for reclassifying histologic grade (HG) 2 tumors into HG1-like or HG3-like groups; Wound-Response (WR) signature [28,29] for classifying tumors into *activated* or *quiescent* WR groups; Hypoxia signature [15,30] for assigning *hypoxic* or *non*-*hypoxic* tumors; 21-gene-recurrence-score (RS) or Oncotype DX® (Genomic Health Inc., Redwood City, CA) [12] for predicting distant recurrence at ten years in adjuvant-tamoxifen-treated patients [12,31] and EndoPredict (EP) [32], a recently developed 11-gene assay for predicting distant recurrence at ten years in ER-positive and HER2-negative patients who were treated with adjuvant hormonal therapy.

We found that the prognostic effects of signatures declined with follow-up time and were generally better in ER-positive than ER-negative disease. In particular, signatures that had strong predictive power in ER-positive disease, mostly had little predictive power in ER-negative disease, the main exception being WR which had some predictive power also in ER-negative disease; on the other hand, Hypoxia was the only signature with clear predictive power in ER-negative disease, but had no predictive power in ER-positive disease. This illustrates the need for designing robust prognostic tools separately for ER-positive and ER-negative disease.

## Methods

Detailed description, together with reproducible code and data, are provided in the Additional files 2, 3 and 4, respectively.

### Microarray Data

The gene expression dataset [33] (n = 947) is a collection of six published breast cancer microarray datasets [26,27,34-37] on Affymetrix Human Genome HG-U133A arrays. The datasets were retrieved from Gene Expression Omnibus [38] (http://www.ncbi.nlm.nih.gov/geo) and ArrayExpress (http://www.ebi.ac.uk/arrayexpress) under accession number GSE6532 [27], GSE3494 [34], GSE1456 [35], GSE7390 [26], GSE2603 [36] and E-TABM-158 [37] respectively. Data were processed and RMA-normalized [39] as previously described [33].

### Clinical data

We compiled comprehensive clinical information on these 947 samples in addition to what have been collected previously [33]. This includes additional and up-to-date (if available) information on ER status [35], node status [35], tumor size [35], and DMFS follow-ups [34,35], and treatment information [26,27,34-37].

Distant Metastasis Free Survival (DMFS: n = 912) was used as clinical endpoint (Additional file 1: Table S2A). Additional file 1: Table S2B summarizes the clinicopathological characteristics with respect to the clinical endpoint. For tumors lacking ER and HER2 status from standard immunohistochemistry (or FISH), the gene expression value for *ESR1* and *ERBB2*, respectively, were used [33]. Among 335 tumors [34,37] with available *TP53* mutation status, 82 tumors were *TP53*-mutated and 253 were wild-type. Pathological characteristics including tumor size (DMFS: n = 905), lymph node status (DMFS: n = 893) and histological grade (DMFS: n = 781) were recorded. Datasets with adjuvant treatment information [26,27,34,35,37] included 403 patients (DMFS: n = 395) who did not receive systemic treatment.

### Applicability of signatures

We investigated all nine signatures (Table 1; Additional file 1: Table S1) on the full dataset (n = 947), although some of the signatures were originally developed on specific patient subgroups. Most analyses were done separately for ER-positive and ER-negative disease. While RS [12] has only been applied to ER-positive breast cancer, and GGI [7,27] was developed on ER-positive and only later validated on ER-negative disease (Supplement), for completion we have included both along with the other signatures in the analyses on ER-negative disease.

**Table 1:** Summary of gene signatures in the study and the annotation mapping coverage

**Signature**	**Description**	**Validation**	**Training platform**	**Coverage (%)**
Intrinsic [1,21]	Intrinsic subtype	Ref. [2,3]	Stanford cDNA array	410/549 (75%)
PAM50 [9]	PAM50 subtype	Ref. [9]	Agilent Human oligo array	42/50 (84%)
70gene [4]	MammaPrint	Ref. [5,22-24]	Agilent 25 k Human oligo array	46/70 (65.7%)
76gene [6]	Veridex	Ref. [25,26]	Affymetrix u133a GeneChip	76/76 (100%)
Hypoxia [30]	Hypoxia signature	Ref. [15,30]	Stanford cDNA array	117/253 (46.2%)^a^
WR [28]	Wound response	Ref. [15,29]	Stanford cDNA array	298/380 (78.4%)
GGI [7]	Genomic Grade Index	Ref. [17,27]	Affymetrix u133a GeneChip	128/128 (100%)
RS [12]	OncoType DX Recurrent Score	Ref. [12,31]	qRT-PCR	21/21 (100%)
EP [32]	EndoPredict risk score	Ref. [32,40]	qRT-PCR	11/11 (100%)

^a^The original study [30] reported 168 UniGene IDs annotated from 253 clones in the Hypoxia signature, of which 117 clones mapped to the Affy probes (46.2%). Considering these mapped clones represented 116 unique Unigene clusters (under UniGene Build Number 222), the mapping coverage for this signature on the studied data is higher than the percentage reported here.

The 76-gene signature has only been validated in node-negative disease [25,26], but we found that it was also a valid predictor on node-positive disease and have therefore assessed it on the full dataset. Indeed, several of the signatures were originally developed on node-negative disease, and later validated on node-positive disease (see Additional file 1: Table S1 for details).

The EP signature [32] was originally designed for ER-positive/HER2-negative breast cancer patients for predicting distant recurrence. In this study, it showed significant prognostic power on the complete dataset, ER-positive/HER2-negative treated and untreated subgroups (Figure eight of Additional file 2).

### Computing original gene signature scores

Affymetrix probes were matched against the genes of the signatures (Table 1). Risk scores were then generated using the original algorithms of the signatures and recalibrated on the studied dataset for risk-group assignments.

For Intrinsic and PAM50, subtype classification was performed based on the nearest of the five centroids (distances calculated using correlation to the centroids). Risk score per sample was computed by linear combination of the centroid correlations in ROR-S model (Risk-Of-Relapse scores by Subtype alone) [9]. A *pseudo Oncotype DX*® *Recurrence Score* per patient was computed by the unscaled Recurrence Score [12]. Similarly, a *pseudo EP Score* per patient was obtained by the unscaled risk score for EP [32]. For 76-genes, GGI, RS and EP, rather than assigning risk groups based on published cutoffs, we used a population-based approach in which a fixed proportion of the population was assigned to each risk group. The proportions were derived from previous datasets associated with individual signatures [7,12,26,40]. We found this necessary as our analyses differed from the original methods in technical or methodological manners (Supplement).

### Survival analysis

Distant Metastasis Free Survival (DMFS: n = 912) is used as clinical endpoint. Follow-up time was defined as time from diagnosis until distant metastasis, or time of last follow-up if the patient is not known to have distant metastasis. It was noted that DMFS in the Pawitan set [35] was defined as distant metastasis or death, whichever occurs first. Since this only consists of a small portion of the studied cohort, it is unlikely to bias or confound our results.

Continuous risk scores from the original signatures were used instead of categorized risk-groups. For Intrinsic and PAM50, the ROR-S scores were used. For 70-gene, the centroid correlations were reversed to represent the risk.

The concordance index [41] (C-index, an analogy to area under ROC curve) was chosen to compare the predictive strength of the signatures. The contribution of a signature predictor in the univariate setting was evaluated using the *proportion of variation explained* in the outcome variable (PVE) [42].

Univariate Cox models were fitted for each risk signature. Assessment of the proportional hazard assumption by different methods [43-45] indicated clear time-dependencies in the predictive power of the risk signatures and was used to identify suitable time intervals for separate Cox analyses. Standardized hazard ratios (HR) indicate the relative risk associated with a one-standard-deviation increase in the risk score.

Effects of common prognostic factors: tumor size (pT1, pT2 and pT3-pT4), node status (positive versus negative) and histological grade (I-III) were investigated using multivariate Cox models.

### METABRIC data

METABRIC [46] expression discovery set (n = 996) was used. Gene annotations on the original IlluminaHT12v3 probes were retrieved using BioMart through R library biomaRt (Ensembl release 68, HG19 human assembly). Disease-specific survival was used as endpoint. Follow-up time was defined as time from diagnosis until death, or time of last follow-up if the patient is not known to have died. Data is available through European Genome-Phenome Archive (http://www.ebi.ac.uk/ega/), under accession number EGAS00000000083.

## Results

### Subtype signatures comparison

We compared the subtype classification between Intrinsic and PAM50 on the full dataset (n = 947). Overall, their subtype assignments were moderately concordant (Cohen’s kappa [47]*κ* = 0 · 54). Noticeably, nearly half of the Intrinsic LumA tumors were assigned as LumB by PAM50 (40.7%), while the two signatures appeared to highly agree on classification of basal-like tumors (86.5%; Additional file 5: Figure S1A). Indeed, basal-like was the most concordant subtype with a Pearson correlation of 0 · 94 between Intrinsic and PAM50 (Additional file 5: Figure S1B), followed by normal-like (0.85), LumA (0.68), LumB (0.55) and Her2-enriched (0.42). More specifically, basal-like was the most distinctly classified subtype across these two signatures (Additional file 5: Figure S1C) with disagreement limited to a few borderline classifications. Furthermore, agreements between the subtypes and their immunohistochemistry receptor status counterparts were similar for both signatures. A majority of the 709 IHC ER-positive samples were classified as Luminal tumors (62% for Intrinsic and 64% for PAM50), and half of the IHC HER2-positive samples were classified as HER2-enriched subtype (55% for Intrinsic and 50% for PAM50). The overlap between Basal-like tumors and triple-negative samples was 79% for both PAM50 and Intrinsic (Figure two and three of Additional file 2).

One property that distinguishes these signatures is that proliferation-associated genes were intentionally added when developing PAM50. This may partially explain the disconcordance between PAM50 and Intrinsic in their LumA and LumB classifications. Both signatures were kept for further analysis.

### Similarity for risk assessment among gene signatures

**Figure 1:**
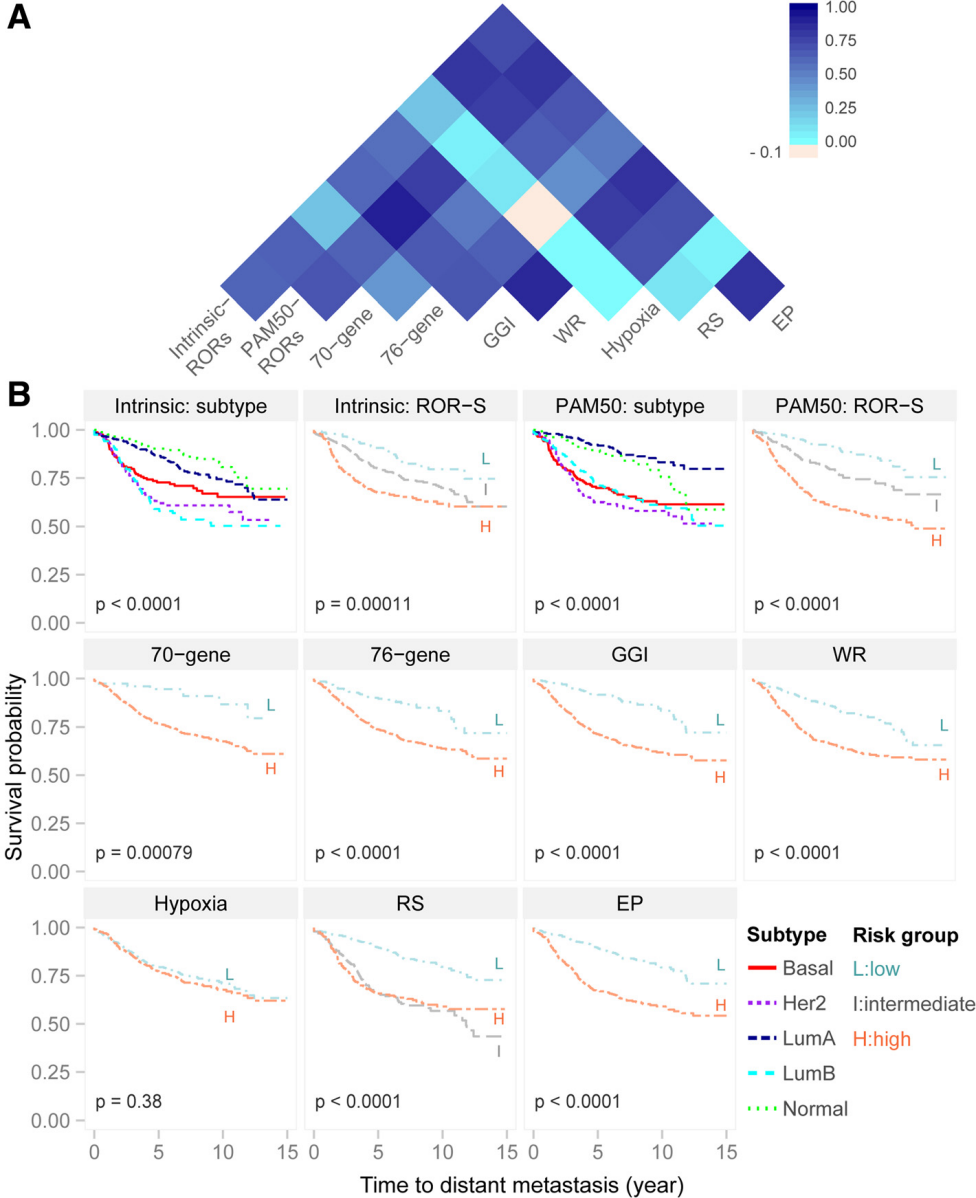
**Risk prediction by gene signatures. (A)** Heatmap of the pairwise correlations of the predicted risk scores from the gene signatures. The predicted risk scores by Intrinsic and PAM50 are generated by the ROR-S (Risk of Relapse by Subtype along) model. The risk predictions are generally fairly concordant across different signatures, except for Hypoxia that has week correlations with the other signatures. **(B)** Comparison of 15-year period prediction for Distant Metastasis Free Survival (DMFS) using risk groups identified by published cutoffs in original gene signatures. Survival probabilities associated with the risk groups are shown by Kaplan Meier plot up to 15 years. For most of the signatures, the reported cutoffs were applied to generate risk group assignments. Thresholds for risk groups assignment were modified for 76-gene, GGI and RS using population based strategy. For 76-gene, “good” prognosis is defined as less than 30% percentile of the raw relapse score in ER + group and less than 22% percentile in ER- group [26]. For GGI, the third of the patients with low GGI scores being defined as low-risk and the remaining patients as high-risk [7]. For RS, 27% patients with high unscaled Recurrence Score were assigned as “high-risk” and 51% with low score as “low-risk”, and the remaining 22% of the patients were assigned to the “intermediate-risk” group [12]. For the Intrinsic signature and PAM50, in addition to the survival curves associated with subtype groups, the risk groups defined by the ROR-S model (risk of relapse subtype-only model) are also shown.

The Pearson correlations of the continuous risk scores from individual signatures were generally high (Figure 1A; Additional file 1: Table S3). The correlations were above 0.4, except those involving Hypoxia and between 76-gene and Intrinsic (ρ=0.23), indicating reasonably good concordance across the signatures. The highest correlations were between GGI and PAM50 (0.9), followed by GGI with WR (0.87) and Intrinsic with RS (0.81). Intrinsic and PAM50 ROR-S scores correlated well (ρ=0.61). The Hypoxia signature was negatively associated with the 76-gene classifier (ρ= - 0.02), and 76-gene was also less in agreement with other signatures: correlation with Intrinsic (0.23), 70-gene (0.4) and RS (0.44). Thus, Hypoxia and 76-gene appear distinct from the other signatures.

### Comparison of performances of gene signatures for survival prediction

For all signatures except Hypoxia, differences in DMFS between risk groups were highly significant (n = 912; Figure 1B).

Using the continuous risk scores to predict DMFS, PAM50 had the highest C-index of 0.658 with 95% CI [0.64–0.68] (Table 2), followed by GGI (0.656), WR (0.651), RS (0.648), 76-gene (0.642), 70-gene (0.612), Intrinsic (0.598) and Hypoxia (0.525). All signatures received a C-index exceeding the threshold 0.5 for random prediction. The importance of individual signatures in univariate setting as measured by PVE (Table 2) ranked PAM50 (5.74%), GGI (4.87%) and WR (4.83%) as the top three predictors for DMFS, while Hypoxia explained the lowest portion of variation (0.6%). The rankings by C-index and PVE were fairly similar.

**Table 2:** Assessment of univariate performance of individual gene signatures on Distant Metastasis Free Survival prediction

	**C**^**a **^**(95% CI)**	**PVE**^**b **^**(%)**
Intrinsic-RORs	0.598 [0.58, 0.62]	1.60
PAM50-RORs	0.658 [0.64, 0.68]	5.74
70-gene	0.612 [0.60, 0.63]	2.76
76-gene	0.642 [0.62, 0.66]	4.58
GGI	0.656 [0.64, 0.67]	4.87
WR	0.651 [0.63, 0.67]	4.83
Hypoxia	0.525 [0.50, 0.55]	0.60
RS	0.648 [0.63, 0.67]	4.05
EP	0.648 [0.63, 0.67]	4.78

^a^C: concordance index.

^b^*PVE*: proportion of variation explained in the outcome variable, comparable with the *R*^*2*^ in regression modeling.

The variability of the C-index was estimated from 1000 bootstrap iterations.

### Time- & ER-dependency of gene signatures for DMFS prediction

The assumption of time-independent proportional hazard was examined for ER-positive group and ER-negative group separately using a univariate Cox model with signature risk scores as covariate. Time-dependency was clearly visible for most of the signatures (Additional file 5: Figure S2A-B; Table 3). In general, signatures seemed to lose their predictive power over time for forecasting DMFS.

**Table 3:** **Time**- &**ER**-**dependent effect assessment of individual gene signatures in predicting Distant Metastasis Free Survival** (**DMFS**)

					***ER *** **+ (*n *** **= *692*)**					***ER *– (*n *** **= *220*)**	
**Gene signature**	**PH**^**a **^**ρ (p)**	**Time**	**n**_***risk***_	**n**_***event***_	**HR [95% CI]**	**p**	**PH**^**a **^**ρ (p)**	**n**_***risk***_	**n**_***event***_	**HR [95% CI]**	**p**
Intrinsic-RORs	-0.23(0.0032)						-0.38(0.0069)				
		0-5 yr	692	126	1.46 [1.24-1.72]	<0.0001		220	61	1.24 [0.94-1.63]	0.1230
		5-10 yr	566	41	0.83 [0.59-1.15]	0.2576		159	7	0.95 [0.49-1.85]	0.8892
		>10 yr	525	10	0.76 [0.36-1.60]	0.4650		152	5	0.49 [0.25-0.98]	0.0440
PAM50-RORs	-0.27(0.0005)						-0.13(0.3676)				
		0-5 yr	692	126	2.16 [1.78-2.61]	<0.0001		220	61	1.11 [0.86-1.44]	0.4312
		5-10 yr	566	41	1.26 [0.93-1.70]	0.1383		159	7	1.6 [0.64-4.03]	0.3161
		>10 yr	525	10	0.91 [0.48-1.73]	0.7773		152	5	0.54 [0.27-1.09]	0.0835
70-gene	-0.18(0.0232)						-0.24(0.0702)				
		0-5 yr	692	126	1.70 [1.43-2.03]	<0.0001		220	61	1.00 [0.79-1.28]	0.9739
		5-10 yr	566	41	1.16 [0.87-1.55]	0.3054		159	7	0.84 [0.43-1.65]	0.6189
		>10 yr	525	10	1.06 [0.57-1.96]	0.8468		152	5	0.40 [0.21-0.78]	0.0066
76-gene	-0.20(0.0092)						-0.26(0.0565)				
		0-5 yr	692	126	1.83 [1.55-2.17]	<0.0001		220	61	1.30 [1.04-1.62]	0.0228
		5-10 yr	566	41	1.32 [0.99-1.76]	0.0551		159	7	1.12 [0.51-2.48]	0.7745
		>10 yr	525	10	0.58 [0.28-1.20]	0.1446		152	5	0.38 [0.09-1.57]	0.1828
GGI	-0.32(<0.0001)						-0.29(0.0296)				
		0-5 yr	692	126	2.11 [1.77-2.52]	<0.0001		220	61	1.07 [0.82-1.38]	0.6288
		5-10 yr	566	41	1.23 [0.91-1.66]	0.1735		159	7	1.22 [0.55-2.71]	0.6170
		>10 yr	525	10	0.73 [0.37-1.44]	0.3592		152	5	0.39 [0.18-0.84]	0.0165
WR	-0.28(0.0001)						-0.43(0.0002)				
		0-5 yr	692	126	2.07 [1.72-2.48]	<0.0001		220	61	1.39 [1.05-1.85]	0.0214
		5-10 yr	566	41	1.16 [0.86-1.56]	0.3341		159	7	0.80 [0.39-1.63]	0.5334
		>10 yr	525	10	0.63 [0.33-1.20]	0.1612		152	5	0.30 [0.12-0.75]	0.0098
Hypoxia	-0.02(0.7717)						-0.04(0.7132)				
		0-5 yr	692	126	1.06 [0.88-1.26]	0.5483		220	61	1.50 [1.20-1.89]	<0.0001
		5-10 yr	566	41	0.93 [0.68-1.27]	0.6427		159	7	1.41 [0.70-2.83]	0.3389
		>10 yr	525	10	1.10 [0.57-2.10]	0.7773		152	5	0.48 [0.17-1.35]	0.1645
RS	-0.25(0.0019)						-0.23(0.0628)				
		0-5 yr	692	126	1.79 [1.55-2.07]	<0.0001		220	61	1.19 [0.92-1.53]	0.1919
		5-10 yr	566	41	1.06 [0.78-1.43]	0.7311		159	7	0.78 [0.39-1.58]	0.4902
		>10 yr	525	10	0.65 [0.26-1.61]	0.3535		152	5	0.57 [0.25-1.30]	0.1798
EP	-0.27(0.0004)						-0.18(0.2457)				
		0-5 yr	692	126	1.97 [1.66-2.33]	<0.0001		220	61	1.11 [0.86-1.45]	0.4199
		5-10 yr	566	41	1.13 [0.83-1.53]	0.4393		159	7	1.04 [0.50-2.15]	0.9198
		>10 yr	525	10	1.02 [0.55-1.91]	0.9462		152	5	0.51 [0.25-1.04]	0.0628

^a^*PH* test for time trend: scaled Schoenfeld residuals were tested against transformed time (Kaplan-Meier estimates) for violation of proportional hazard assumption in a univariate Cox model for individual gene signatures. P values are shown.

Analysis was carried out on ER + group and ER- group separately. Preliminary test for time trend was performed by checking proportional hazard assumption in a Cox model^§^ per signature fitted on all tumors with follow-up time and event status available (column “PH”: correlation and asscoaited p value are reported)). Main effect associated with a signature for DMFS prediction in a certain follow-up time interval was estimated by a Cox model within each ER stratification. The Hazard Ratio (*HR*) along with its 95% confidence interval and the p value from the Wald test are shown. Numbers of patients at risk (n_*risk*_) were computed at time point 0, 5 and 10 year, respectively.

To investigate the nature of time-dependency in ER-positive tumors, we inspected the cumulative regression plots of the estimate along with 95% confidence intervals from a univariate additive regression model (Additional file 5: Figure S2C). The estimated curve in each plot reflects the cumulative effect of a signature covariate on survival over time, and a time-independent effect should therefore result in a curve with a constant slope. Hypoxia did not seem to have an effect on DMFS prediction. For all the other signatures, there were significant and strong initial positive effects up to around 5 years; these effects tended to disappear after about 10 years. However, the estimates are uncertain towards the end of the time span as few patients remain in the risk set. In ER-negative breast cancers (Additional file 5: Figure S2D), while similar time-dependency was evident for individual signatures, the effects on DMFS predictions were less substantial than in the ER-positive subset, and rather uncertain for most of the signatures. Contrary to its non-predictive behavior in the ER-positive group, Hypoxia predicted DMFS (higher hypoxic scores associated with a shorter survival time) for ER-negative cancers. In addition, WR, 76-gene and Intrinsic also potentially have predictive effect in the early follow-up period.

**Figure 2:**
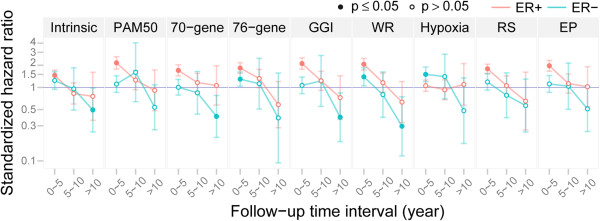
**Evaluation of time- & ER-dependency in predicting Distant Metastasis Free Survival (DMFS) by gene signatures (n = 912).** Estimated effect (standardized hazard ratios, e^β^, with 95% confidence intervals) of gene signatures for survival prediction within different time intervals and stratified by ER status. The X-axis indicates the follow-up time intervals: up to 5-year, 5–10 year, and beyond 10 year. Within each subinterval, a univariate Cox model per signature was fitted. The Y-axis indicates the estimated hazard ratios (HR) on a logarithmic scale corresponding to a 1 standard deviation increase in the signature. The null, HR = 1, is indicated by the blue line. Solid dots indicate HRs significantly different from 1 (P < 0 · 05). ER + (n = 692) is denoted as red and ER– (n = 220) is denoted as blue. The number of events for each follow-up subinterval in ER + subgroup is 126, 41 and 10, respectively; and in ER- subgroup 61, 7 and 5, respectively.

Based on these results, we divided follow-up time into three intervals: first 5 years, 5–10 years, and beyond 10 years. Patients experiencing an event before the start of the interval were excluded, while those that remained at risk at the end of the time interval were censored. For each time interval, univariate Cox models for each signature were fitted in ER-positive and ER-negative tumors separately. The estimated HRs with 95% confidence interval per time interval and ER status are shown for each signature (Figure 2; Table 3). The HRs were systematically higher at earlier time points and decayed with time; predictions were generally stronger in the ER-positive group than in the ER-negative group. Within the first 5 years, all signatures except for Hypoxia had significant positive effects (p < 0.0001) in the ER-positive group; while in the ER-negative group, Hypoxia (p < 0.0001), WR (p = 0.021) and 76-gene (p = 0.023) were the only classifiers with significant positive effects on DMFS prediction. We observed borderline significant protective effects (HR < 1: higher risk scores had lower risks for distant metastasis) within the last time interval (>10 years) in the ER-negative group for Intrinsic (p = 0.044), 70-gene (p = 0.007), GGI (p = 0.017) and WR (p = 0.01).

### Possible effects of cohort differences

Since cohort differences could potentially lead to spurious effects, we ran survival analyses adjusted for cohort differences. However, as cohort differences did not contribute significantly to the models (Figure one and Box five of Additional file 2), it seems unlikely that cohort differences may have biased the results.

### Analysis on a systemically untreated subpopulation

To avoid bias introduced by adjuvant treatment, the same analyses were performed on patients that were only treated with surgery with/without radiotherapy (n = 395). Similar indications related to follow-up time and ER status for signatures predicting DMFS hold in this subgroup of patients (Additional file 5: Figure S3), indicating that treatment alone does not explain the effects described above.

Analyses of systemically treated patients confirmed the predictive power of the signatures during the first 5 years of follow-up in the ER-positive group, but had too few events after 5 years for any reliable assessment of time-dependency.

### Multivariate analysis on signatures with known prognostic parameters

Node status, tumor size and histological grade all significantly predict DMFS on the complete dataset (n = 912; Additional file 5: Figure S4A). A multivariate Cox model was fitted with node, size, histological grade and individual signatures for the two ER groups separately. In the ER-positive group (Additional file 1: Table S4A), with the exception of Hypoxia (p = 0.7351), signatures remain significant with the presence of size, node and histological grade (Model 1: p < 0.0001 except Intrinsic p = 0.0397). Inclusion of tumor size in the model removed the time trends associated with the signatures (Model 2). The prognostic power of the included predictors were dismal for ER-negative tumors (Additional file 1: Table S4B),

### Analysis on prognosis of gene signatures associated with HER2 status for DMFS prediction

We investigated the performance of gene signatures in relation to HER2 status. We observe a decreasing time dependency associated with the prognostic power in the HER2-negative group (Additional file 5: Figure S5A). Due to limited number of events in the 5–10 year followup interval, we cannot draw conclusions about the time trend in the HER2-positive group and the differences in prognostic power between the two HER2 groups (Additional file 5: Figure S5A).

The analysis on groups defined by both HER2 status and ER status revealed a decreasing time trend for the signature’s prognostic power for both the HER2-/ER + and HER2-/ER- groups (Additional file 5: Figure S5B), where at least two events are presented for each time intervals. And HER2-/ER + is generally better than HER2-/ER- in term of prognostic power. This can be largely explained by the ER stratification.

### Validation on METABRIC data

We observed similar ER-dependency and similar pattern of gene signatures for the long-term prognosis on the METABRIC complete set, systemically untreated set as well as on the systemically treated set (Additional file 5: Figure S6 & Additional file 1: Table S7). Similarly, including histological grade and tumor size seems to reduce the strength of the time dependency of the signatures (Additional file 1: Table S8).

## Discussion

### Applicability of individual gene signatures

Growing evidence suggests that expression-based gene signatures are of clinical relevance, especially for identifying patients at high risk of early distant metastasis. One important challenge is to robustly identify patients with low risk, thereby reducing the number of patients receiving cytotoxic treatment. Translating signatures to a new dataset is complicated by differences in microarray platforms and data processing procedures, as well as the clinical differences between cohorts.

Methods based on centroid correlations (e.g. subtype signatures, 70-gene and WR) and methods that transform the data into an invariant scale before computing the risk scores (e.g. GGI) have more consistent performances across different studies. We suspect that summarizing gene expression patterns through weighted averages (e.g. 76-gene, RS, Hypoxia) is more sensitive to data scales and missing gene information. Different normalization procedure from the original study [6] may explain why the original 76-gene signature, prior to the population-based recentering, did not predict any good prognosis in our data. Generally, when the distribution of risk scores depends on platform and normalization procedure, cutoffs for risk group assignment need to be recalibrated. The population-based strategy is more general and applicable for a study with a pure prognostic purpose, but requires the tumors to be representative of the population of breast cancer.

### Time- and ER-dependency of prognostic gene signatures

Prognostication by gene-expression signatures seemed harder for ER-negative than for ER-positive tumors. It should be noted that most of the signatures have been trained on populations containing a majority of ER-positive tumors. All studied signatures except for Hypoxia showed prognostic power in assessing DMFS in ER-positive breast cancer in the first few years after diagnosis. Only the 76-gene, Wound-Response and Hypoxia signatures were prognostic in the ER-negative group within the first five years. The time-dependent prognostic effect was previously reported for the 76-gene [26] and RS [31].

**Figure 3:**
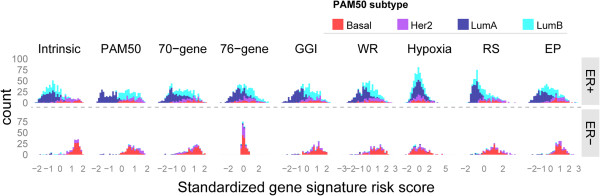
**Gene**-**signature risk scores in relation to biological entities**. Distributions of the subtypes (called by PAM50) stratified by ER status for individual gene signatures. Note that we used PAM50-classifications as proxy of proliferation. Luminal tumors dominate ER-positive group, while basal and Her2-enriched tumors drive the risk score higher in ER-negative group. In the ER-positive stratum, the risk assessment in most of the gene signatures is highly consistent with classification of luminal A and B tumors. Being more proliferative is known to distinguish luminal B tumors from luminal As. This indicates that the proliferation module, underlying many signatures, may drive the prognostication in ER-positive tumors.

Most of the signatures were tightly correlated. We believe this may be due to common underlying biological processes. Studies [16,27,48-50] suggest that cell proliferation is a common characteristic among many signatures (e.g. 76-gene, 70-gene, RS, GGI, PAM50, WR). If the proliferation module drives prognostication in ER-positive tumors, the risk-group separation will be highly comparable to the classification of LumA and LumB tumors within the ER-positive subgroup, as LumB tumors are characterized by higher proliferation. This seemed to be the case for the majority of the signatures (Figure 3). Different signatures essentially detect the low-proliferation subset as low-risk in the ER-positive group [27,48,49]. Furthermore, histological grade, which strongly reflects proliferation, shows prognostic value only in the ER-positive subgroup (Additional file 5: Figure S4B; ER + p = 0.0002 vs ER- p = 0.57). This highlights the need for robust prognostic tools designed for each ER subgroup.

The dismal performances in ER-negative tumors of most of the signatures, except 76-gene and Hypoxia, resulted from classifying most of them into the high-risk category [48,51]. This elevated risk score was predominantly driven by highly proliferative basal-like and Her2-enriched tumors (Figure 3), and left the signatures with poor discriminative power for risk assessment within ER-negative tumors. Clinically, patients with ER-negative tumors are heterogeneous with respect to age as well as treatment received. Most patients with ER-negative tumors receive cytotoxic chemotherapy. All these factors pose difficulties in marker identification and further building prognostic/predictive signatures specific for this subgroup. The ER-specific markers within the 76-gene signature (60 genes from ER + and 16 from ER-) contribute to its prognostic ability in both ER stratifications. Intriguingly, signatures characterizing tumor microenvironment (Hypoxia and Wound-Response) showed prognostic values for ER-negative breast cancer. In line with previous indications [18,30], Hypoxia seems to carry biological and prognostic information distinct from the other signatures (Figure 1A). More specifically, certain genetic components and the microenvironment of breast tumors are likely to be important for the predictive ability of the Hypoxia signature. Tumors with “high hypoxia response” were more likely to have TP53 mutations and to be ER negative [30]. In this study, *TP53*-mutated and ER-negative tumors had elevated hypoxic score (one-tailed t-test p = 0.029; Additional file 5: Figure S4C), while no significant differences in the hypoxic score associated with *TP53* status were found in the ER-positive tumors (one-tailed t-test p = 0.29). Distinct features of the tumor microenvironment associated with basal-like and luminal tumors [52] possibly underlie the variation in hypoxia responses observed in different ER subgroups.

Proliferation seems to be the common driving force for prognostication in ER-positive breast cancers, while different biological mechanisms such as stress response may be crucial for risk stratification in ER-negative tumors. Additionally, immune-related gene modules have been implicated to be prognostic in high-risk ER-positive breast cancers [53] and ER-negative breast cancers [54,55].

In most gene expression studies, information on patient treatment is limited and inconsistent. In our combined cohort, treatment data were compiled for systemic adjuvant treatment. Results for patients that did not receive systemic treatment (Additional file 5: Figure S3A-C) were consistent with the main findings. Data on patient cohorts homogeneously treated is important to be able to distinguish between ability to predict treatment response to a specific therapy and prediction of prognosis.

In multivariate analyses on the ER-positive tumors (Additional file 1: Table S4A), signatures remained powerful predictors and added significant information beyond known prognostic parameters, including tumor size, node and histological grade. Histological grade lost much of its prognostic power in models with signature, size and node (Model 1). The signatures’ change in prognostic power over time fell or disappeared in models that included histological grade (Model 3) or tumor size (Model 2). More advanced tumors, grade-3 or large tumor size, tended to experience early relapse, with late relapse more common in less advanced tumors (grade-1 or small tumor size). The inclusion of histological grade or tumor size in the model may thus have captured and masked some of the time-dependency of the signatures’ prognostic power (see Additional file 1: Table S5, Additional file 1: Table S6 and Additional file 5: Figure S4D-E for more detail), although it also indicates that signatures may provide more accurate long-term prognosis when combined with information on histological grade or tumor size. Multivariate analyses on the ER-negative group were not presented because none of the included predicators was significant.

We did not find any notable effect of cohort differences on our analyses (Figure one and Box five of Additional file 2).

The METABRIC set [46] served as an independent validation set for our study. We did not have access to this data until after the original analyses had been performed. The fact that we are able to confirm the observations from the original analyses (based on the meta-cohort; n = 947) in an independent large dataset, undoubtedly validates our study, greatly strengthens the indications and authenticates the conclusions. These findings were confirmed in both the systemically treated and untreated groups, and thus does not seem to be affected by the use of breast cancer specific survival as event instead of DMFS. We did not include the classification for molecular subtype proposed by Curtis et al. [46] as the IC (Integrative Cluster) subgroups are based on clustering on both gene expression and copy number data through a joint latent model [56]. The majority of samples in our main analysis did not have copy number data available, while evaluating the ICs in METABRIC together with other signatures would bias the results since the IC classification was developed using this cohort.

The indications from our study that prognostic power of gene signatures depend on ER-status, has previously been reported by Desmedt et al. [49]. They used a gene module score to estimate HER2 and ER activity, and used this to split the samples by HER2 status, and the HER2-negative were further split by ER status, resulting in three groups. We used ER and HER2 status based on IHC where available, or imputed from gene expression if not. Since we did not see a substantial effect of HER2 status on DMFS or time dependency (Additional file 5: Figure S5), we did not focus on stratification based on the HER2 status.

By the rule of thumb that 10 events per covariate is generally sufficient for Cox analyses [57-60], we consider the sample size and number of events sufficient to reliably assess the prognostic power of gene signatures in the different follow-up time intervals for both ER states (Table 3 & Additional file 1: Table S7A), although with some reservations for the last time interval (>10 years) for the ER-negative group which had few events (n = 5 in both studied datasets). However, we consider our results convincing given the consistency across the two datasets and across several signatures.

It is interesting to observe that higher risk scores associated with lower risks for distant metastasis after >10 years follow-up in the ER-negative group. These estimates are based on a small number of events (n = 5 in both datasets), but the fact that it occurs in both datasets lends the finding some credibility. For ER-negative cases still under study after 10 years, high risk signatures tended to correspond to higher histological grade and HER2 positive status.

### Compatibility between signatures and target cohort

Some of the signatures had been developed on specific patient subgroups (Additional file 1: Table S1). In particular, several of them were developed on node-negative disease and only later validated on node-positive disease. Signatures developed on one patient subgroup, may be expected to have reduced power on other patient subgroups despite later validation, and so the use of a signature from one patient group extended to a larger group should be done and interpreted with caution.

Specifically in our study, the 76-gene signature is intended for lymph-node-negative cancers. However, since it was predictive for the node-positive patients as well (p = 0.005 for raw relapse scores predicting DMFS, see Supplement), we judged that the 76-gene signature was a valid predictor also for node-positive disease, and could be assessed along with the other signatures without substantial loss of predictive power.

Although the RS was used as a prognostic test in the tamoxifen-treated breast cancers, we found that RS had significant prognostic power for the ER-positive patients in the untreated cohort as well (Additional file 5: Figure S3C). The RS signature was only intended for ER-positive, and so cannot be criticized for performing badly on ER-negative. Indeed, it performed no worse than many of the other signatures, which were intended to cover ER-negative disease.

The EP signature was designed as a prognostic test in ER-positive, HER2-negative breast cancer patients treated with adjuvant endocrine therapy only. We found that the EP had significant prognostic power on the ER-positive, HER2-negative, untreated cancers, as well as the complete set (Supplement). As the treatment information in our main analysis is limited to systemic treatment, the stratified subset for EP is not strictly based on adjuvant endocrine therapy only.

## Conclusions

In summary, our study highlights conditions under which it is appropriate to use individual published gene signatures for survival prediction. The distinctions in prognostic behavior of the signatures with respect to ER status suggest that different molecular mechanisms are involved in risk stratifications within each ER stratum. Also, the signatures were primarily able to predict relapse with the first 5 years of follow-up, with little ability to predict later relapses. Incorporating characteristics of the advancement of the tumor might help improve the quality of the prognosis, perhaps also with respect to long-term prognosis. While the majority of the tested signatures are strong risk predictors in the early follow-up time intervals for ER-positive tumors, there are urgent needs to improve risk stratifications for long-term prognosis and ER-negative breast cancers.

## Abbreviations

70-gene: 70-gene gene signature (MammaPrint®); 76-gene: 76-gene gene signature; DMFS: Distant Metastasis Free Survival; ER: Estrogen receptor; GGI: Genomic Grade Index; HR: hazard ratio; Hypoxia: Hypoxia gene signature; Intrinsic: Intrinsic signature; PAM50: PAM50 signature; ROR: Risk Of Relapse; RS: 21-gene-recurrence-score (Oncotype DX®); WR: Wound Response signature.

## Competing interests

The authors declare that they have no competing interests.

## Author’s contributions

Conceived of and designed the study: XZ EAR ALBD OCL. Organized and analyzed the data: XZ. Interpreted the results: XZ EAR TS HKMV HGR ALBD OCL. Wrote the paper: XZ EAR TS. Contributed to manuscript and discussions: HKMV HGR VNK OCL ALBD. All authors read and approved the final manuscript.

## Grant support

This study was supported by Norwegian Research Council (NFR) FUGE program, Grant 175240 and NFR Cancer program, Grant 193387. The funders had no role in study design, data collection and analysis, decision to publish, or preparation of the manuscript.

## Pre-publication history

The pre-publication history for this paper can be accessed here:

http://www.biomedcentral.com/1471-2407/14/211/prepub

## Supplementary Material

Additional file 1: Table S1 Characteristics of the studied gene signatures and the breast cancer cohorts they were developed from and validated on. **Table S2.** Summary of the studied cohort (n = 947). **Table S3.** The pairwise Pearson correlations matrix of the predicted risk scores on continuous scale identified by individual gene signatures. **Table S4.** Multivariate analysis on gene signatures with known prognostic factors. **Table S5.** Univariate analysis on gene signatures with G1, G2, G3 separately in ER + samples. **Table S6.** Univariate analysis on gene signatures with T1, T2, T3 separately in ER + samples. **Table S7.** Time- & ER-dependent effect assessment of individual gene signatures in predicting Disease-specific Survival on the METABRIC set. **Table S8.** Time trend analysis on METABRIC set. Click here for file

Additional file 2 Supplement. Click here for file

Additional file 3 Sweave file containing reproducible report for Zhao et al. Click here for file

Additional file 4 Rnw source file containing code and text used to create Additional file 3. Click here for file

Additional file 5 Supplementary figures. Click here for file
