# Supplementary material for: Systematic assessment of prognostic gene signatures for breast cancer shows distinct influence of time and ER status
Source: BMC Cancer. 2014 Mar 19;14:211. doi: 10.1186/1471-2407-14-211 (PMC4000128; doi:10.1186/1471-2407-14-211)
Supplement: Additional file 1: Table S1 — Characteristics of the studied gene signatures and the breast cancer cohorts they were developed from and validated on. Table S2. Summary of the studied cohort (n = 947). Table S3. The pairwise Pearson correlations matrix of the predicted risk scores on continuous scale identified by individual gene signatures. Table S4. Multivariate analysis on gene signatures with known prognostic factors. Table S5. Univariate analysis on gene signatures with G1, G2, G3 separately in ER + samples. Table S6. Univariate analysis on gene signatures with T1, T2, T3 separately in ER + samples. Table S7. Time- & ER-dependent effect assessment of individual gene signatures in predicting Disease-specific Survival on the METABRIC set. Table S8. Time trend analysis on METABRIC set. [file 1471-2407-14-211-S1.pdf]

Table S1. Characteristics of the studied gene signatures and the breast cancer cohorts they were developed from and validated on.

Signature	*Predicted phenotype/endpoint*	*Training Cohort*	*Validation Cohort*
Intrinsic	Subtypes	Locally advanced BC [1]	Consecutive BC [a] [2, 3]
PAM50	Subtypes<br>5-year relapse	Consecutive BC [9]<br>Node– [9]	Consecutive BC [9, 61]<br>Node– & + [9, 61]
70gene	5 year distant metastasis	Node– [4]	Node– & + [5, 22-24]
76gene	5 year distant metastasis	Node– [6]	Node– [25, 26]
GGI	HG1- or HG3-like in HG2	ER+ [7]	Consecutive BC [17, 27]
WR	Active or Quiescent CSR<br>Population based prognosis	Representative BC [b] [28]	Representative BC [15, 29]<br>Consecutive BC
Hypoxia	Hypoxic or Non-hypoxic	Representative BC [30]	Representative BC [15, 30]
RS	10 year distant metastasis	Tamoxifen-treated/ER+;Node–[12]	Tamoxifen-treated/ER+;Node–,+[12, 31]
EP	10 year distant metastasis	ER+, HER2-, treat by adjuvant hormonal therapy	ER+, HER2-, treat by adjuvant hormonal therapy

[a] *Consecutive BC*: heterogeneous breast cancer cohort with consecutive clinical parameter distribution as reflected in the whole population of this disease.
[b] *Representative BC*: breast cancer dataset at hand carries representative features that are associated with a certain breast cancer subpopulation.

Table S2. Summary of the studied cohort (n=947). (A) Sample sizes for available clinical endpoints of the included studies. Summary was calculated using samples with both survival time and status available. Distant Metastasis Free Survival (DMFS) was selected as endpoint due to its clinical and biological implications. Being able to metastasize to distant organs makes cancer a deadly disease. DMFS is an endpoint that impacts treatment decisions. In addition, information for DMFS is available across all included studies. **(B)** Clinicopathological characteristics of the studied cohort for Distant Metastasis Free Survival.

(A)

			DMFS			*BCSS*			*RFS*			*OS*	
Dataset	*n*	***DMFS***	n_{event}	t_{med}	*BCSS*	n_{event}	t_{med}	*RFS*	n_{event}	t_{med}	*OS*	n_{event}	t_{med}
Desmedt [26]	147	√	48	130·7		-	-	√	72	103·8	√	43	139·6
Miller [34]	247	√	69	78·0	√	54	124·0	√	92	75·0		-	-
Minn [36]	96	√	26	65·4		-	-		-	-		-	-
Pawitan [35]	156	√ [a]	46	81·5	√	29	84·7	√	40	85·6	√	40	84·7
Loi [27]	178	√	36	72·1		-	-	√	59	66·6		-	-
Chin [37]	123	√	25	67·6	√	28	72·8	√	37	67·6	√	41	72·8
n	947	**912**	250	80·8	525	111	92·8	842	300	78	425	124	91·1

(B)

**Characteristics**		**n**	**%** [b]
All		912	
Estrogen receptor (IHC)			
	Positive	687	76·1
	Negative	216	23·9
	Missing	9	
Estrogen receptor (IHC + gene expression)	Positive	692	75·9
	Negative	220	24·1
	Missing	0	
Human Epidermal Growth Factor Receptor 2 (IHC)			
	Positive	16	15.4
	Negative	88	84.6
	Missing	808	
Human Epidermal Growth Factor Receptor 2 (IHC + gene expression)	Positive	114	12.5
	Negative	798	87.5
	Missing	0	
Progesterone receptor (IHC)			
	Positive	291	65.1
	Negative	156	34.9
	Missing	465	
Histological Grade			
	I	155	19·8
	II	354	45·3
	III	272	34·8
	Missing	131	
Tumor status			
	pT1 (≤ 2cm)	431	47·6
	pT2 (> 2 ≤ 5cm)	441	48·7
	pT3-4 (> 5cm)	33	3·6
	missing	7	
Nodal status			
	Positive	291	32·6
	Negative	602	67·4
	Missing	19	

Systemic Treatment			
	Yes	436	52·5
	No	395	47·5
	Unknown[c]	81	
Event			
	Yes	250	27·4
	Censored	662	72·6
Median follow-up (month)		80·8	

Abbreviations:
DMFS: Distant Metastasis Free Survival
BCSS: Breast Cancer Specific overall Survival
RFS: Relapse Free Survival
OS: Overall Survival
$n_{event:}$: number of events
t_{med}: median follow-up time in month

[a] In Pawitan set, a modified DMFS was used as an event was defined as distant metastasis or death.
[b] For all parameters, numbers are calculated excluding missing data.
[c] Treatment information was not provided for the Minn dataset.[36]

Table S3. The pairwise Pearson correlations matrix of the predicted risk scores on continuous scale identified by individual gene signatures.

	Intrinsic-RORs	PAM50-RORs	70-gene	76-gene	GGI	WR	Hypoxia	RS	EP
Intrinsic-RORs	1	0·611	0·627	0·228	0·602	0·566	0·223	0·809	0·724
PAM50-RORs	0·611	1	0·677	0·626	0·903	0·777	0·051	0·777	0·814
70-gene	0·627	0·677	1	0·403	0·662	0·529	0·093	0·649	0·679
76-gene	0·228	0·626	0·403	1	0·671	0·634	-0·024	0·436	0·525
GGI	0·602	0·903	0·662	0·671	1	0·868	0·006	0·786	0·827
WR	0·566	0·777	0·529	0·634	0·868	1	0·009	0·692	0·696
Hypoxia	0·223	0·051	0·093	-0·024	0·006	0·009	1	0·098	0·041
RS	0·809	0·777	0·649	0·436	0·786	0·692	0·098	1	0·823
EP	0·724	0·814	0·679	0·525	0·827	0·696	0·041	0·823	1

Table S4. Multivariate analysis on gene signatures with known prognostic factors.

(A) In ER-positive samples. Using multivariate Cox model, prognosis of individual signature was adjusted by clinical parameters. The following Cox models were fitted: (**Model 1**) *Risk = Node + Grade + Tumor size + Signature*; **(Model 2**) *Risk = Tumor size + Signature;*(**Model 3**) *Risk = Grade + Signature*. Results from univariate models are presented for comparison: (**Univariate**) *Risk = Signature* as well as similar univariate models for each of the clinical parameters. Models were fitted on the complete data set with available information for endpoint and included clinical parameters (n=586) and on the entire follow-up time. For each Cox model, parameters were added sequentially into the model in the order specified in the table, and the added (type I) effect of each subsequent covariate is reported under *Analysis of deviance* as the reduction in the residual deviance χ^2 with its corresponding p value. Time trend associated with individual gene signature covariate was assessed by testing for violation of proportional hazard (PH) assumption in the corresponding Cox model ("*Time trend for signature"*). Violation of the PH assumption (significant p value for correlation ρ) indicates significant time dependency of individual signatures on prognosis.

	Univariate		***Model 1***		***Model 2***		***Model 3***	
**Deviance (type I)**	χ^2	p	χ^2	p	χ^2	p	χ^2	p
Clinical variables:								
Node (df=1)	21.5	<0·0001	21.5	<0·0001	-	-	-	-
Tumor size (df=2)	36.4	<0·0001	22	<0·0001	36.4	<0·0001	-	-
Histological grade (df=2)	16.3	0·0003	12.6	0.0018	-	-	16.3	0·0003
Gene signature (df=1):								
Intrinsic-RORs	11.8	0·0006	4.2	0.0397	9.7	0.0018	5.1	0.0242
PAM50-RORs	53.2	<0·0001	31.9	<0·0001	44.3	<0·0001	38.1	<0·0001
70-gene	36.0	<0·0001	21.5	<0·0001	32.8	<0·0001	24.9	<0·0001
76-gene	40.6	<0·0001	18.8	<0·0001	30.6	<0·0001	27.1	<0·0001
GGI	55.0	<0·0001	36.1	<0·0001	46.6	<0·0001	40.6	<0·0001
WR	45.3	<0·0001	25.4	<0·0001	34.1	<0·0001	31.6	<0·0001
Hypoxia	0.01	0.9275	0.1	0.7351	0.2	0.6501	0.1	0.7317
RS	43.6	<0·0001	23.1	<0·0001	34.3	<0·0001	30.2	<0·0001
EP	53.6	<0·0001	31.4	<0·0001	43.8	<0·0001	38.8	<0·0001
**Time trend for signature**	ρ	p	ρ	p	ρ	p	ρ	p
Intrinsic-RORs	-0.233	0.0032	-0.160	0.0584	-0.042	0.6121	-0.209	0.0109
PAM50-RORs	-0.269	0·0005	-0.154	0.0698	0.008	0.9211	-0.183	0.0226
70-gene	-0.180	0.0232	-0.156	0.0658	-0.044	0.591	-0.211	0.0102
76-gene	-0.202	0.0092	-0.160	0.0514	-0.022	0.7835	-0.205	0.0100
GGI	-0.324	<0·0001	-0.145	0.0888	0.009	0.9108	-0.186	0.0224
WR	-0.283	0·0001	-0.146	0.0879	0.017	0.8391	-0.192	0.0189
Hypoxia	-0.020	0.7717	-0.161	0.0576	-0.067	0.418	-0.219	0.0073
RS	-0.250	0.0019	-0.146	0.0866	-0.006	0.9446	-0.193	0.0185
EP	-0.274	0·0004	-0.148	0.0831	0.004	0.9642	-0.189	0.0195

(B) In ER-negative samples. The following Cox models were fitted: (**Model 1**) *Risk = Node + Signature;* (**Model 2**) *Risk = Tumor size + Signature* and (**Model 3**) *Risk = Grade + Signature* and (**Model 4**) *Risk = Node + Tumor size + Grade + Signature.* Results from univariate models are presented for comparison: (**Univariate**) *Risk = Signature* as well as similar univariate models for each of the clinical parameters. Models were fitted on the complete data set with available information for endpoint and included clinical parameters (n=174) and on the entire follow-up time.

Deviance (type I)	*Univariate* χ^2	*Univariate* p	*Model 1* χ^2	*Model 1* p	*Model 2* χ^2	*Model 2* p	*Model 3* χ^2	*Model 3* p	*Model 4* χ^2	*Model 4* p
Clinical variables:										
Node (df=1)	1.9	0.1659	1.9	0.1659	-	-	-	-	1.9	0.165[illegible]
Tumor size (df=2)	0.6	0.7342	-	-	0.6	0.7342	-	-	0.4	0.839[illegible]
Histological grade (df=2)	1.3	0.5248	-	-	-	-	1.3	0.5248	1.3	0.531[illegible]
Gene signature (df=1):										
Intrinsic-RORs	0.7	0.408	0.6	0.4219	0.8	0.3814	1.3	0.251	1.3	0.255[illegible]
PAM50-RORs	0.2	0.6827	0.2	0.6785	0.2	0.6853	0.5	0.4726	0.4	0.543[illegible]
70-gene	0.6	0.4381	0.6	0.4355	0.5	0.4589	0.3	0.5989	0.3	0.594[illegible]
76-gene	4.1	0.0427	4.6	0.0311	4.3	0.0379	4.2	0.0396	5	0.024[illegible]
GGI	0	0.9934	0	0.975	0	0.9805	0.1	0.7337	0.1	0.763[illegible]
WR	0.8	0.3589	1.3	0.2594	1	0.3151	1.3	0.2607	1.7	0.191
Hypoxia	3.2	0.0734	4	0.0462	3.2	0.0758	3.4	0.0642	4.1	0.042
RS	0.5	0.468	0.6	0.4208	0.5	0.4719	1.2	0.2651	1.2	0.269[illegible]
EP	0.1	0.7334	0.2	0.6695	0.1	0.7808	0.5	0.5006	0.4	0.525[illegible]
**Time trend for signature**	ρ	**p**	ρ	**p**	ρ	**p**	ρ	**p**	ρ	**p**
Intrinsic-RORs	-0.377	0.0069	-0.173	0.2094	-0.171	0.2074	-0.185	0.1875	-0.171	0.193[illegible]
PAM50-RORs	-0.127	0.3676	-0.182	0.1873	-0.220	0.1108	-0.214	0.1175	-0.186	0.157[illegible]
70-gene	-0.244	0.0702	-0.180	0.1912	-0.205	0.1266	-0.221	0.1056	-0.175	0.181[illegible]
76-gene	-0.260	0.0565	-0.215	0.1069	-0.248	0.0657	-0.206	0.1303	-0.212	0.092
GGI	-0.294	0.0296	-0.191	0.1644	-0.165	0.2325	-0.206	0.1284	-0.181	0.167[illegible]
WR	-0.432	0.0002	-0.260	0.0503	-0.089	0.5184	-0.150	0.2677	-0.222	0.081[illegible]
Hypoxia	-0.038	0.7132	-0.194	0.1579	-0.227	0.099	-0.219	0.1093	-0.167	0.205[illegible]
RS	-0.226	0.0628	-0.190	0.1647	-0.178	0.1902	-0.209	0.1271	-0.174	0.186[illegible]
EP	-0.184	0.2457	-0.195	0.154	-0.195	0.1564	-0.212	0.1196	-0.183	0.162[illegible]

Table S5. Univariate analysis on gene signatures with G1, G2, G3 separately in ER+ samples.

(A) Analysis of deviance & time trend test. Prognosis of individual signature was tested in univariate Cox model: *Risk = Signature* in ER+ group (n= 715; column ***Univariate***), G1 samples of the ER+ group, (n= 153; column ***G1***), G2 samples of the ER+ group, (n= 311; column ***G2***) and G3 samples of the ER+ group, (n=149; column ***G3***). For each Cox model, the added (type I) effect of each subsequent covariate is reported under *Analysis of deviance* as the reduction in the residual deviance χ^2 with its corresponding p value. Time trend associated with individual gene signature covariate was assessed by testing for violation of proportional hazard (PH) assumption in the corresponding Cox model ("*Time trend for signature"*). Violation of the PH assumption (significant p value for correlation ρ) indicates significant time dependency of individual signatures on prognosis.

	ER+		*ER+ / G1*		*ER+ / G2*		*ER+ / G3*	
**Deviance (type I)**	χ^2	p	χ^2	p	χ^2	p	χ^2	p
Gene signature (df=1):								
Intrinsic-RORs	9.8	0.0018	<0·0001	0.8443	5.1	0.0238	0.5	0.4877
PAM50-RORs	58	<0·0001	15.8	0·0001	24.5	<0·0001	2.1	0.1434
70-gene	31.2	<0·0001	3.9	0.0469	27.1	<0·0001	0.1	0.7575
76-gene	42.9	<0·0001	12.8	0·0003	11.2	0. 0008	7	0.008
GGI	56.1	<0·0001	10.5	0.0012	23.2	<0·0001	6.4	0.0113
WR	46.5	<0·0001	6	0.0144	19	<0·0001	7.3	0.0069
Hypoxia	0.1	0.723	<0·0001	0.9628	1.2	0.2737	0	0.9412
RS	42.4	<0·0001	2.2	0.1395	19.6	<0·0001	6.2	0.0125
EP	49.5	<0·0001	7.5	0.0061	21	<0·0001	9	0.0028
**Time trend for signature**	ρ	p	ρ	p	ρ	p	ρ	p
Intrinsic-RORs	-0.233	0.0032	-0.228	0.3061	-0.161	0.1738	0.161	0.3238
PAM50-RORs	-0.269	0·0005	-0.700	0·0005	-0.107	0.3355	0.131	0.3856
70-gene	-0.180	0.0232	-0.458	0.0215	-0.101	0.4128	0.282	0.0423
76-gene	-0.203	0.0092	-0.529	0.007	0.001	0.9963	-0.042	0.7763
GGI	-0.324	<0·0001	-0.667	0.0034	-0.149	0.2146	-0.021	0.8881
WR	-0.283	0·0001	-0.565	0.0037	-0.112	0.2966	-0.049	0.7045
Hypoxia	-0.020	0.7717	-0.144	0.3479	0.033	0.7404	-0.153	0.3078
RS	-0.250	0.0019	-0.541	0.0597	-0.154	0.2079	0.200	0.1711
EP	-0.274	0. 0004	-0.641	0.0079	-0.204	0.1016	0.152	0.2448

(B) Standardized Hazard Ratio. Risk scores for individual gene signatures were standardized, and prognostic effects of signatures were compared using Hazard Ratio from univariate Cox model: Risk = *Signature* in ER+ group (n= 715; column ***ER+***), G1 samples of the ER+ group, (n= 153; column ***ER+/G1***), G2 samples of the ER+ group, (n= 311; column ***ER+/G2***) and G3 samples of the ER+ group, (n=149; column ***ER+/G3***).

	ER+		*ER+ / G1*		*ER+ / G2*		*ER+ / G3*	
Gene signature	**HR[95%CI]**	p	**HR[95%CI]**	p	**HR[95%CI]**	p	**HR[95%CI]**	p
Intrinsic-RORs	1.26 [1.09-1.45]	0.0014	1.04 [0.7-1.54]	0.8431	1.28 [1.04-1.58]	0.0213	1.1 [0.84-1.45]	0.4871
PAM50-RORs	1.8 [1.54-2.1]	<0·0001	2.13 [1.49-3.05]	<0·0001	1.76 [1.41-2.21]	<0·0001	1.24 [0.92-1.67]	0.1587
70-gene	1.51 [1.31-1.74]	<0·0001	1.45 [1.02-2.07]	0.0391	1.8 [1.44-2.25]	<0·0001	1.04 [0.79-1.38]	0.7579
76-gene	1.62 [1.4-1.87]	<0·0001	1.98 [1.39-2.83]	0·0002	1.45 [1.17-1.79]	6.00E-04	1.46 [1.1-1.93]	0.009
GGI	1.76 [1.52-2.03]	<0·0001	1.79 [1.3-2.45]	0·0003	1.71 [1.38-2.12]	<0·0001	1.47 [1.08-2]	0.0155
WR	1.69 [1.45-1.96]	<0·0001	1.59 [1.11-2.28]	0.0114	1.64 [1.32-2.05]	<0·0001	1.51 [1.11-2.06]	0.0083
Hypoxia	1.03 [0.88-1.2]	0.7225	0.99 [0.64-1.53]	0.9628	1.14 [0.9-1.43]	0.2707	0.99 [0.75-1.3]	0.9413
RS	1.58 [1.39-1.79]	<0·0001	1.26 [0.95-1.68]	0.1036	1.56 [1.3-1.87]	<0·0001	1.44 [1.08-1.92]	0.0135
EP	1.69 [1.46-1.96]	<0·0001	1.66 [1.18-2.32]	0.0032	1.67 [1.35-2.08]	<0·0001	1.56 [1.15-2.1]	0.0038

Table S6. Univariate analysis on gene signatures with T1, T2, T3 separately in ER+ samples.

(A) Analysis of deviance & time trend test. Prognosis of individual signature was tested in univariate Cox model: Risk = *Signature* in ER+ group (n= 715; column ***ER+***), T1 samples of the ER+ group, (n= 349; column ***ER+/T1***), T2 samples of the ER+ group, (n= 335; column ***ER+/T2***) and T3 samples of the ER+ group, (n=26; column ***ER+/T3***). For each Cox model, the added (type I) effect of each subsequent covariate is reported under *Analysis of deviance* as the reduction in the residual deviance χ^2 with its corresponding p value. Time trend associated with individual gene signature covariate was assessed by testing for violation of proportional hazard (PH) assumption in the corresponding Cox model ("*Time trend for signature"*). Violation of the PH assumption (significant p value for correlation ρ) indicates significant time dependency of individual signatures on prognosis.

	ER+		***ER+ / T1***		***ER+ / T2***		***ER+ / T3***	
**Deviance (type I)**	χ^2	p	χ^2	p	χ^2	p	χ^2	p
Gene signature (df=1):								
Intrinsic-RORs	9.8	0.0018	1.1	0.2901	8.2	0.0043	0.3	0.569
PAM50-RORs	58	<0·0001	18.2	<0·0001	28.5	<0·0001	2	0.159
70-gene	31.2	<0·0001	8.2	0.0042	18.2	<0·0001	0.8	0.3653
76-gene	42.9	<0·0001	22.2	<0·0001	12.5	0·0004	1.9	0.1685
GGI	56.1	<0·0001	15.9	0·0001	28.8	<0·0001	3.7	0.0531
WR	46.5	<0·0001	11.6	0·0006	21.2	<0·0001	3.7	0.0543
Hypoxia	0.1	0.723	0	0.8616	0.5	0.4996	0.1	0.7227
RS	42.4	<0·0001	11.7	0·0006	23.5	<0·0001	0.3	0.6108
EP	49.5	<0·0001	11.1	0·0009	29.2	<0·0001	0.9	0.3487
**Time trend for signature**	ρ	p	ρ	p	ρ	p	ρ	p
Intrinsic-RORs	-0.233	0.0032	-0.452	0·0006	-0.117	0.2537	0.306	0.4464
PAM50-RORs	-0.269	0·0005	-0.178	0.1766	-0.266	0.0083	0.182	0.664
70-gene	-0.180	0.0232	-0.241	0.0821	-0.135	0.1812	-0.005	0.9866
76-gene	-0.203	0.0092	-0.108	0.4056	-0.241	0.0166	0.309	0.4765
GGI	-0.324	<0·0001	-0.202	0.1397	-0.355	0·0004	0.270	0.4749
WR	-0.283	0·0001	-0.165	0.1981	-0.311	0·0005	0.536	0.2178
Hypoxia	-0.020	0.7717	-0.103	0.377	0.083	0.3817	-0.241	0.3351
RS	-0.250	0.0019	-0.343	0.0139	-0.217	0.0365	0.292	0.4172
EP	-0.274	0. 0004	-0.274	0.028	-0.249	0.0171	0.031	0.9275

(B) Standardized Hazard Ratio. Risk scores for individual gene signatures were standardized, and prognostic effects of signatures were compared using Hazard Ratio from univariate Cox model: Risk = *Signature* in ER+ group (n= 715; column ***ER+***), T1 samples of the ER+ group, (n= 349; column ***ER+/T1***), T2 samples of the ER+ group, (n= 335; column ***ER+/T2***) and T3 samples of the ER+ group, (n=26; column ***ER+/T3***).

	ER+		***ER+ / T1***		***ER+ / T2***		***ER+ / T3***	
Gene signature	**HR[95%CI]**	p	**HR[95%CI]**	p	**HR[95%CI]**	p	**HR[95%CI]**	p
Intrinsic-RORs	1.26 [1.09-1.45]	0.0014	1.15 [0.89-1.47]	0.2834	1.32 [1.09-1.58]	0.0036	1.22 [0.61-2.43]	0.5679
PAM50-RORs	1.8 [1.54-2.1]	<0·0001	1.74 [1.35-2.25]	<0·0001	1.71 [1.4-2.1]	<0·0001	1.62 [0.82-3.19]	0.1626
70-gene	1.51 [1.31-1.74]	<0·0001	1.43 [1.12-1.82]	0.004	1.51 [1.25-1.84]	<0·0001	1.35 [0.72-2.54]	0.3538
76-gene	1.62 [1.4-1.87]	<0·0001	1.8 [1.42-2.29]	<0·0001	1.4 [1.16-1.68]	4.00E-04	1.67 [0.78-3.58]	0.1893
GGI	1.76 [1.52-2.03]	<0·0001	1.64 [1.3-2.09]	<0·0001	1.71 [1.4-2.08]	<0·0001	2.09 [0.93-4.69]	0.075
WR	1.69 [1.45-1.96]	<0·0001	1.54 [1.21-1.97]	0·0005	1.59 [1.3-1.95]	<0·0001	1.95 [0.93-4.12]	0.0783
Hypoxia	1.03 [0.88-1.2]	0.7225	0.98 [0.75-1.27]	0.8617	1.07 [0.88-1.3]	0.4974	1.14 [0.56-2.32]	0.7199
RS	1.58 [1.39-1.79]	<0·0001	1.49 [1.2-1.84]	0·0002	1.58 [1.32-1.89]	<0·0001	1.18 [0.64-2.15]	0.5979
EP	1.69 [1.46-1.96]	<0·0001	1.53 [1.2-1.96]	7.00E-04	1.68 [1.39-2.04]	<0·0001	1.44 [0.67-3.08]	0.3459

Table S7. Time- & ER-dependent effect assessment of individual gene signatures in predicting Disease-specific Survival on the METABRIC set. (A) On the complete METABRIC set; (B) On the systemically untreated samples and (C) On the systemically treated samples . Main effect associated with a signature for Disease-specific Survival prediction in a certain follow-up time interval was estimated by a Cox model within each ER stratification. The Hazard Ratio (HR) along with its 95% confidence interval and the p value from the Wald test are shown. Numbers of patients at risk (n_{risk}) were computed at time point 0, 5 and 10 year, respectively.

(A)

		ER + (n=801)				*ER – (n=195)*			
**Gene signature**	**Time**	n_{risk}	n_{event}	**HR [95% CI]**	**p**	n_{risk}	n_{event}	**HR [95% CI]**	**p**
Intrinsic-RORs									
	0-5yr	801	101	2.00 [1.66-2.41]	<0·0001	195	73	1.11 [0.87-1.43]	0.4039
	5-10yr	700	80	0.96 [0.77-1.20]	0.7223	122	10	0.67 [0.47-0.95]	0.0235
	>10yr	620	37	1.03 [0.74-1.43]	0.8423	112	5	0.83 [0.38-1.80]	0.6328
PAM50-RORs									
	0-5yr	801	101	2.20[1.78-2.72]	<0·0001	195	73	1.03 [0.82-1.30]	0.7855
	5-10yr	700	80	1.32 [1.07-1.64]	0.0107	122	10	0.68 [0.45-1.03]	0.0658
	>10yr	620	37	1.04 [0.75-1.43]	0.8285	112	5	0.27 [0.09-0.81]	0.0187
70-gene									
	0-5yr	801	101	1.99 [1.63-2.41]	<0·0001	195	73	0.95 [0.76-1.18]	0.6242
	5-10yr	700	80	1.11 [0.89-1.38]	0.3514	122	10	0.74 [0.43-1.29]	0.2877
	>10yr	620	37	1.21 [0.87-1.68]	0.2545	112	5	0.85 [0.39-1.89]	0.6986
76-gene									
	0-5yr	801	101	1.57 [1.31-1.87]	<0·0001	195	73	0.89 [0.70-1.14]	0.3622
	5-10yr	700	80	1.23 [0.99-1.52]	0.0574	122	10	1.32 [0.73-2.42]	0.3605
	>10yr	620	37	0.76 [0.54-1.09]	0.1346	112	5	1.27 [0.56-2.88]	0.5727
GGI									
	0-5yr	801	101	1.91 [1.59-2.31]	<0·0001	195	73	1.11 [0.88-1.42]	0.3799
	5-10yr	700	80	1.35 [1.09-1.67]	0.0060	122	10	0.83 [0.44-1.57]	0.5736
	>10yr	620	37	0.87 [0.62-1.22]	0.4269	112	5	0.24 [0.07-0.88]	0.0318
WR									
	0-5yr	801	101	1.70 [1.41-2.06]	<0·0001	195	73	1.28 [1.00-1.65]	0.0509
	5-10yr	700	80	1.15 [0.93-1.43]	0.1974	122	10	0.97 [0.53-1.79]	0.9320
	>10yr	620	37	0.84 [0.6-1.17]	0.2950	112	5	0.36 [0.13-1.04]	0.0602
Hypoxia									
	0-5yr	801	101	1.32 [1.11-1.56]	0.0015	195	73	1.12 [0.90-1.40]	0.3011
	5-10yr	700	80	1.09 [0.88-1.36]	0.4194	122	10	0.76 [0.36-1.57]	0.4554
	>10yr	620	37	1.26 [0.88-1.80]	0.2102	112	5	1.47 [0.50-4.27]	0.4837
RS									
	0-5yr	801	101	1.94 [1.69-2.24]	<0·0001	195	73	1.22 [0.99-1.51]	0.0669
	5-10yr	700	80	1.11 [0.89-1.38]	0.3481	122	10	0.60 [0.33-1.10]	0.0975
	>10yr	620	37	1.19 [0.86-1.65]	0.2856	112	5	1.19 [0.56-2.54]	0.6535
EP									
	0-5yr	801	101	1.96 [1.64-2.33]	<0·0001	195	73	0.91 [0.74-1.13]	0.4069
	5-10yr	700	80	1.29 [1.04-1.59]	0.0183	122	10	0.73 [0.41-1.28]	0.2690
	>10yr	620	37	1.13 [0.82-1.55]	0.4593	112	5	0.71 [0.26-1.93]	0.5050

(B)

Gene signature	Time	*ER + (n=203)*				*ER – (n=50)*			
		n_{risk}	n_{event}	HR [95% CI]	p	n_{risk}	n_{event}	HR [95% CI]	p
Intrinsic-RORs									
	0-5yr	203	12	1.67 [1.03-2.71]	0.0367	50	13	0.75 [0.47-1.21]	0.2407
	5-10yr	191	24	1.14 [0.78-1.67]	0.491	37	2	2.3 [0.23-23.05]	0.4788
	>10yr	167	12	0.59 [0.27-1.32]	0.1994	35	0	-	-
PAM50-RORs									
	0-5yr	203	12	2.22 [1.27-3.88]	0.0049	50	13	0.97 [0.58-1.64]	0.9191
	5-10yr	191	24	1.4 [0.95-2.05]	0.0881	37	2	4.81 [0.3-76.04]	0.2645
	>10yr	167	12	0.85 [0.46-1.57]	0.6022	35	0	-	-
70-gene									
	0-5yr	203	12	3.25 [1.94-5.45]	<0.0001	50	13	0.68 [0.42-1.11]	0.121
	5-10yr	191	24	1.68 [1.14-2.48]	0.0083	37	2	49.96 [0.21-11631.36]	0.1596
	>10yr	167	12	0.78 [0.43-1.42]	0.4183	35	0	-	-
76-gene									
	0-5yr	203	12	1.97 [1.18-3.32]	0.0101	50	13	0.83 [0.42-1.65]	0.5916
	5-10yr	191	24	1.64 [1.14-2.38]	0.0082	37	2	1.02 [0.18-5.62]	0.9839
	>10yr	167	12	0.61 [0.35-1.08]	0.0919	35	0	-	-
GGI									
	0-5yr	203	12	2.24 [1.33-3.77]	0.0025	50	13	0.99 [0.58-1.71]	0.9787
	5-10yr	191	24	1.68 [1.17-2.41]	0.0051	37	2	3.43 [0.51-23.07]	0.2043
	>10yr	167	12	0.72 [0.4-1.3]	0.2712	35	0	-	-
WR									
	0-5yr	203	12	2.06 [1.18-3.59]	0.0111	50	13	1.35 [0.76-2.42]	0.3077
	5-10yr	191	24	1.49 [1.01-2.2]	0.0455	37	2	1.63 [0.34-7.69]	0.5391
	>10yr	167	12	0.71 [0.41-1.23]	0.223	35	0	-	-
Hypoxia									
	0-5yr	203	12	1.33 [0.83-2.14]	0.2352	50	13	1.25 [0.69-2.28]	0.4552
	5-10yr	191	24	1.05 [0.71-1.56]	0.7919	37	2	0.57 [0.12-2.61]	0.4675
	>10yr	167	12	0.98 [0.52-1.85]	0.9439	35	0	-	-
RS									
	0-5yr	203	12	1.86 [1.23-2.79]	0.0031	50	13	1.21 [0.66-2.23]	0.532
	5-10yr	191	24	1.05 [0.71-1.53]	0.8169	37	2	1.04 [0.25-4.35]	0.9546
	>10yr	167	12	0.94 [0.5-1.76]	0.8476	35	0	-	-
EP									
	0-5yr	203	12	1.75 [1.08-2.85]	0.0229	50	13	0.74 [0.46-1.2]	0.2208
	5-10yr	191	24	1.58 [1.12-2.22]	0.0092	37	2	3.98 [0.74-21.42]	0.108
	>10yr	167	12	0.96 [0.53-1.73]	0.895	35	0	-	-

(C)

Gene signature	Time	ER + (n=598)				ER – (n=145)			
		n_{risk}	n_{event}	HR [95% CI]	p	n_{risk}	n_{event}	HR [95% CI]	p
Intrinsic-RORs									
	0-5yr	598	89	1.99 [1.62-2.45]	<0.0001	145	60	1.21 [0.89-1.64]	0.2269
	5-10yr	509	56	0.87 [0.67-1.14]	0.3228	85	8	0.63 [0.45-0.87]	0.0048
	>10yr	453	25	1.07 [0.75-1.53]	0.7023	77	5	0.89 [0.46-1.73]	0.7357
PAM50-RORs									
	0-5yr	598	89	2.05 [1.63-2.57]	<0.0001	145	60	1.02 [0.79-1.31]	0.8705
	5-10yr	509	56	1.25 [0.96-1.61]	0.0962	85	8	0.41 [0.24-0.7]	0.0011
	>10yr	453	25	1.01 [0.7-1.46]	0.969	77	5	0.4 [0.2-0.81]	0.0111
70-gene									
	0-5yr	598	89	1.76 [1.43-2.18]	<0.0001	145	60	1.02 [0.79-1.32]	0.8568
	5-10yr	509	56	0.9 [0.69-1.18]	0.4468	85	8	0.52 [0.29-0.95]	0.0324
	>10yr	453	25	1.26 [0.85-1.86]	0.2485	77	5	0.96 [0.42-2.19]	0.9274
76-gene									
	0-5yr	598	89	1.45 [1.2-1.76]	0.0002	145	60	0.86 [0.66-1.12]	0.267
	5-10yr	509	56	1.07 [0.82-1.38]	0.6245	85	8	1.33 [0.71-2.51]	0.3763
	>10yr	453	25	0.85 [0.55-1.29]	0.443	77	5	1.23 [0.59-2.57]	0.5853
GGI									
	0-5yr	598	89	1.77 [1.45-2.18]	<0.0001	145	60	1.09 [0.83-1.42]	0.535
	5-10yr	509	56	1.19 [0.92-1.54]	0.196	85	8	0.57 [0.27-1.19]	0.1348
	>10yr	453	25	0.89 [0.59-1.34]	0.5863	77	5	0.24 [0.06-0.91]	0.0363
WR									
	0-5yr	598	89	1.56 [1.27-1.91]	<0.0001	145	60	1.22 [0.92-1.6]	0.1677
	5-10yr	509	56	1.01 [0.78-1.32]	0.9172	85	8	0.81 [0.41-1.6]	0.5433
	>10yr	453	25	0.87 [0.59-1.28]	0.4812	77	5	0.32 [0.11-0.95]	0.0404
Hypoxia									
	0-5yr	598	89	1.29 [1.07-1.55]	0.0064	145	60	1.07 [0.84-1.36]	0.5828
	5-10yr	509	56	1.1 [0.85-1.41]	0.4786	85	8	0.8 [0.35-1.85]	0.6055
	>10yr	453	25	1.26 [0.84-1.9]	0.2621	77	5	1.47 [0.51-4.22]	0.477
RS									
	0-5yr	598	89	1.91 [1.63-2.24]	<0.0001	145	60	1.18 [0.94-1.49]	0.1505
	5-10yr	509	56	1.11 [0.85-1.43]	0.4434	85	8	0.53 [0.27-1.02]	0.0579
	>10yr	453	25	1.15 [0.79-1.66]	0.4602	77	5	1.11 [0.53-2.31]	0.7872
EP									
	0-5yr	598	89	1.91 [1.58-2.31]	<0.0001	145	60	0.94 [0.74-1.2]	0.6272
	5-10yr	509	56	1.12 [0.86-1.46]	0.3846	85	8	0.56 [0.32-0.97]	0.0397
	>10yr	453	25	1.11 [0.76-1.62]	0.6028	77	5	0.65 [0.24-1.77]	0.3996

Table S8. Time trend analysis on METABRIC set. Analysis is carried out on ER-positive and ER-negative groups separately. Time trend associated with individual gene signature covariate was assessed by testing for violation of proportional hazard (PH) assumption in the corresponding Cox model (“*Time trend for signature”*). Violation of the PH assumption (significant p value for correlation ρ) indicates significant time dependency of individual signatures on prognosis. The following Cox models were fitted: (**Model 1**) *Risk = Signature;* (**Model 2**) *Risk = Signature + Grade*; (**Model 3**) *Risk = Signature + TumorSize.*

	**Model 1**		***Model 2***		***Model 3***	
**ER+**	ρ	**p**	ρ	**p**	ρ	**p**
Intrinsic-RORs	-0.3038	<0·0001	0·0003	0.9963	-0.0245	0.7195
PAM50-RORs	-0.2832	0·0001	0.0199	0.7697	0.0023	0.9728
70-gene	-0.2533	0·0001	0.0116	0.8646	-0.0048	0.9439
76-gene	-0.3112	0·0001	0.0185	0.7844	0.0038	0.9548
GGI	-0.3364	<0·0001	0.0298	0.6610	0.0093	0.8916
WR	-0.3317	<0·0001	0.0146	0.8304	0.015	0.8250
Hypoxia	-0.0215	0.7391	-0.0016	0.9816	-0.0237	0.7286
RS	-0.2572	0·0001	0.0044	0.9479	-0.0159	0.8151
EP	-0.2661	0·0003	0.0139	0.8375	-0.0077	0.9097
**ER-**	ρ	**p**	ρ	**p**	ρ	**p**
Intrinsic-RORs	-0.299	0.0030	0.0318	0.7683	0.1517	0.1547
PAM50-RORs	-0.2512	0.0493	-0.0025	0.9808	0.1915	0.0674
70-gene	-0.0987	0.3992	-0.0594	0.5871	0.1594	0.1339
76-gene	-0.0259	0.8024	-0.0607	0.5726	0.1627	0.1281
GGI	-0.2564	0.0144	-0.0299	0.7782	0.1681	0.1122
WR	-0.2792	0.0050	-0.0317	0.7651	0.1642	0.1218
Hypoxia	-0.0432	0.7032	-0.0579	0.5895	0.1763	0.1056
RS	-0.1254	0.2382	-0.0374	0.7272	0.1725	0.1040
EP	-0.0998	0.3776	-0.055	0.6115	0.1668	0.1163
