# Supplementary material for: Systematic assessment of prognostic gene signatures for breast cancer shows distinct influence of time and ER status
Source: BMC Cancer. 2014 Mar 19;14:211. doi: 10.1186/1471-2407-14-211 (PMC4000128; doi:10.1186/1471-2407-14-211)
Supplement: Additional file 2 — Supplement. [file 1471-2407-14-211-S2.pdf]

Supplemental text for

Systematic assessment of prognostic gene signatures for breast cancer shows distinct influence of time and ER status

Xi Zhao, Einar Andreas Rødland, Therese Sørlie, Hans Kristian Moen Vollan, Hege G. Russnes, Vessela N. Kristensen , Ole Christian Lingjærde, Anne-Lise Børresen-Dale.

Contents

I. GENE SIGNATURES OVERVIEW **2**

II. SUPPLEMENTARY METHODS **4**

i. Data **4**

ii. Gene annotation mapping **5**
Annotation for Affy947 expression set 6
Intrinsic gene signature 6
PAM50 6
70-gene signature 6
Wound response gene signature 7
Hypoxia signature 7
76-gene signature 8
Genomic Grade Index (GGI) 8
Oncotype DX (21-gene Genomic Health signature) 8
EndoPredict (EP signature) 8

iii. Gene signatures & the associated classifers **9**
Intrinsic gene signature & PAM50 9
70-gene signature 12
Wound response gene signature 12
Hypoxia signature 13
76-gene signature (Veridex) 13
Genomic Grade Index (GGI) 15
Oncotype DX (21-gene Genomic Health signature) 17
EndoPredict 19

iv. Statistical analysis **22**
Model evaluation: concordance index (C-index) 22
Model evaluation: proportion of variation explained (PVE) 22
Time-dependent effect estimation 22
Software 23

III. SUPPLEMENTARY DISCUSSION ON SIGNATURE CONSTRUCTION STRATEGIES **24**

IV. SUPPLEMENTARY RESULTS **25**
Multivariate analysis on gene signature 25

V. REFERENCE **33**

I. Gene signatures overview

We investigated nine gene signatures that have received the greatest clinical interest and been validated in multiple studies or datasets.

The subtype signature is used to classify breast tumors into five biological subgroups: luminal A, luminal B, HER2-enriched, basal-like, and normal-like. The original intrinsic classification was obtained through unsupervised clustering of 496 genes showing small intra-tumor variation and large inter-tumor variation (before and after neoadjuvant chemotherapy).[1] A couple of variants using different gene sets have emerged in subsequent publications[2-5] to define breast cancer subtypes. In this study, we evaluated the *Intrinsic signature*[1-3, 6] and *PAM50*.[7] Even though PAM50 is the most recent variant, there is at present no consensus on molecular taxonomy.[8]

The 70-gene profile or MammaPrint® (Agendia, Amsterdam, The Netherlands)[9-13] predicts metastasis free survival over a five-year period. It was validated subsequently in the NKI295 cohort consisting of both node negative and node positive patients[10], on another cohort of 241 breast cancer patients with 1–3 positive lymph nodes[11] and in the TRANSBIG consortium.[12] In addition, the results obtained with the 70-gene expression profile were shown to be reproducible with quantitative reverse-transcriptase polymerase chain reaction (qRT-PCR).[13]

The 76-gene signature (Veridex)[14-16] is designed to predict distant metastasis within 5 years for lymph-node-negative breast cancer patients. It was originaly developed based on 286 lymph-node-negative breast cancer patients [14] and later validated in an independent multicentric population of 180 untreated node-negative breast cancer patients [15] and another gene expression study of 198 node-negative breast cancer patients [16] from the same Affymetrix U133a platform used in the original study.[14]

The genomic grade index (GGI) [17, 18] has been designed to reclassify patients with histologic grade 2 tumors into two groups with distinct clinical outcomes similar to those of histologic grade 1 and 3, respectively.

The wound response (WR) or core serum response (CSR) gene signature[19, 20] was derived from the transcriptional response of normal fibroblasts to serum in cell culture. It classifies tumors into two classes (*Activated* vs. *Quiescent*) by comparing the averaged fibroblast serum-induced expression pattern of the CSR genes.

The epithelial hypoxia signature consists of a set of genes for which the expression was consistently induced by hypoxia in cultured epithelial cells (HMECs and RPTECs)[21]. A “hypoxia score” [22] was used to classify tumors as *hypoxic* or *non-hypoxic*.

We included Oncotype DX® (Genomic Health Inc., Redwood City, CA) [23] in our study. The 21-gene recurrence-score signature was developed from qRT-PCR assay to quantify the likelihood of distant recurrence at 10 years in adjuvant-tamoxifen-treated (ER-positive) patients in both node-negative [23] and node-positive disease [24].

Lastly, we also included EndoPredict [25], which is a recent introduced qRT-PCR based test for predicting the likelihood of distant recurrence in patients with ER-positive, HER2-negative breast cancer treated with adjuvant endocrine therapy only.

II. Supplementary Methods

i. Data

The datasets are accessible from NCBI's Gene Expression Omnibus (GEO, http://www.ncbi.nlm.nih.gov/geo/) with the following identifiers; GSE6532 for the Loi *et al.* dataset[18], GSE3494 for Miller dataset[26], GSE1456 for the Pawitan *et al.* dataset[27], GSE7390 for the Desmedt *et al.* dataset[16] and GSE2603 for the Minn *et al*. datset[28]. The Chin et al.[29] dataset is available from ArrayExpress (http://www.ebi.ac.uk/arrayexpress/experiments/E-TABM-158). All overlapping samples from the Desmedt and Loi datasets were excluded.

This pooled dataset (n = 947) was preprocessed and normalized as described previously[30]. Briefly, microarray quality-control assessment was carried out using the Bioconductor package AffyPLM[31]. The Relative Log Expression (RLE) test and the Normalized Unscaled Standard Errors (NUSE) test were applied. Chip pseudo-images were produced to assess artifacts on arrays that did not pass the preceding quality control tests. Selected arrays were normalized according to a three step procedure using the RMA expression measure algorithm (http://www.bioconductor.org[32]): RMA background correction convolution, median centering of each gene across arrays separately for each data set and quantile normalization of all arrays. The merged dataset did not show batch effect (Figure 1).

For samples that the IHC (Immunohistochemistry) ER status were unavailable, the ER status was determined by the expression level of the ER probe “205225_at”: ER negative when 205225_at ≤ -1.84 and ER positive when 205225_at > -1.84. The threshold was selected by ROC curve using the IHC assignments as true labels as previously described[30]. Similarly, for tumors without IHC HER2 status, their HER2 status was determined by expression level of ERBB2 probe “216836_S_at” [30].

The clinical data with updated follow-ups (March 2011 version on Distant Metastasis Free Survival, Relapse Free Survival and Disease Specific Survival) for Miller set[26] is downloadable from https://array.nci.nih.gov/caarray/project/mille-00271 (accessed on May 9, 2012). The treatment data for Pawitan dataset[27] was obtained from the author of the original study.

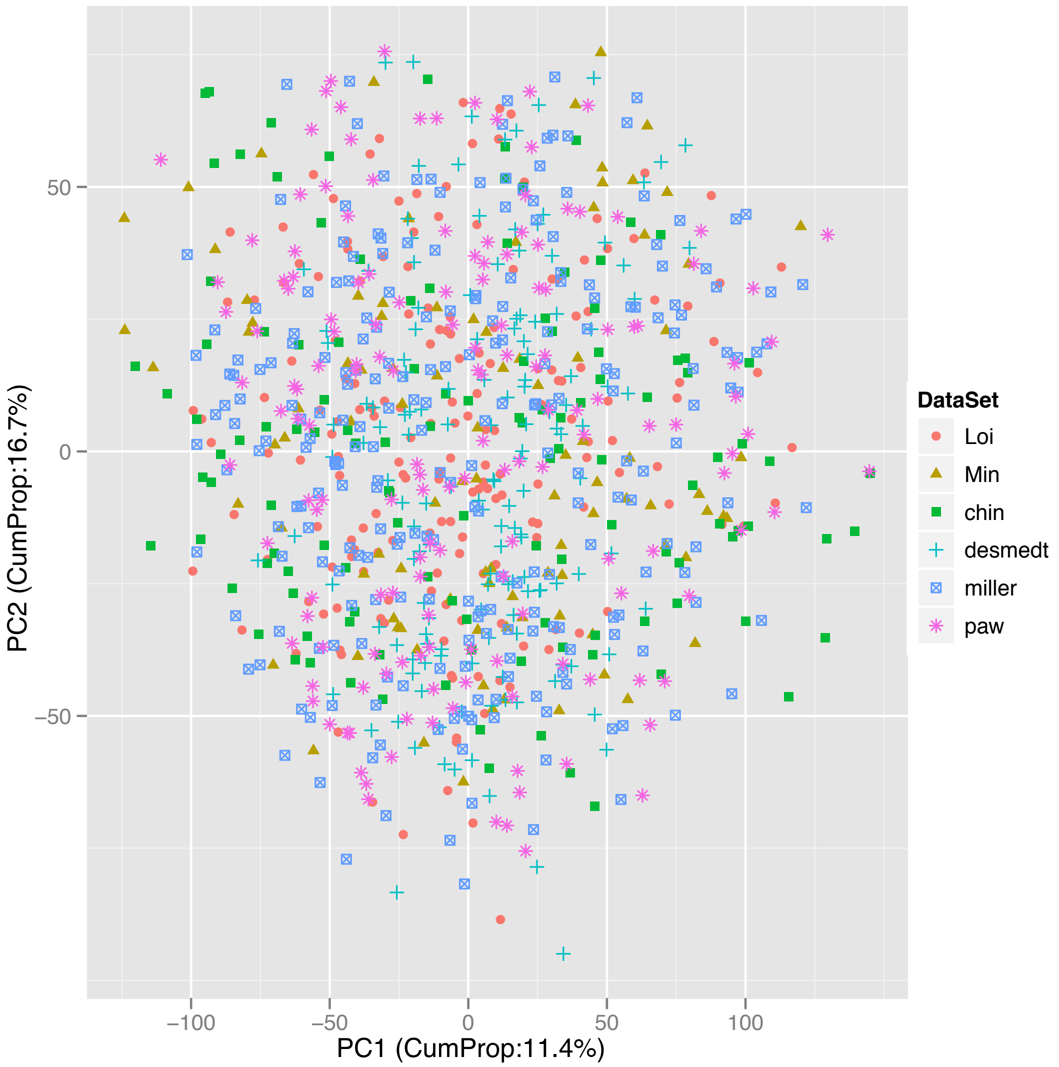

Figure 1. Batch effect examination for the Affy947 data merged from six published breast cancer expression sets.

ii. Gene annotation mapping

For gene signatures that were indexed by Affymetrix U133a probes, the original Affymetix probes in the gene signature were mapped directly to the expression set in the study. For gene signatures with identities other than affymetrix probes, the annotation mapping was carried out using proper identity linker(s).

In the case when multiple hits (Affy IDs in the studied dataset) for a target gene (in the signature) were found, we selected probe(s) with maximum interquartile range (IQR: difference between the third and first quartiles). If there were still more than one hit per target gene, we further averaged the expression values of those probes for each sample. The same *aggregating procedure* for multiple hits of a target gene was used when mapping all the gene signatures.

Annotation for Affy947 expression set

The gene annotations for the Affymetrix U133a probes in the studied expression set were retrieved from BioMart (http://www.ebi.ac.uk/biomart) through Bioconductor[33] implementation biomaRt[34] (Ensembl release 54/NCBI36 (hg18) human assembly).

Intrinsic gene signature

The intrinsic signature[1-3] was developed on Stanford cDNA two-color array. The centroids were indexed by 549 unique clone IDs. Annotations for Stanford 43k cDNA array were retrieved from SMD SOURCE (http://smd.stanford.edu/cgi-bin/source/sourceSearch) under UniGene Build Number 222. The probe identifiers were mapped to the cDNA clones using the following linkers: entrez gene ID, refseq and UniGene cluster ID. We reported all matched probe ID(s) for individual clone ID obtained by each of the linkers. And for matched probes shared the same clone ID, we carried out the aggregating procedure described above: selected probe(s) with maximum IQR, and If there were still more than one hit per clone ID, we further averaged the expression values of those probes for each sample.

Cross-platform mapping coverage: 410/549≈75%

PAM50

The PAM50 gene signature[7] was developed from Agilent human 1Av2 microarrays or custom-designed Agilent human 22k arrays. The reported centroids were indexed by 50 unique gene symbols. The Affymetrix U133A probes in the expression set were mapped to the gene identifications in the signature by gene symbols. And for matched probes shared the same gene symbol identifications, the aggregating procedure described above was carried out.

Cross-platform mapping coverage: 42/50≈84%

70-gene signature

The 70-gene signature was developed from the Rossetta Agilent Hu 25k platform. The centroid was downloaded from the publication site[9] (www.rii.com/download/nejm_table3.zip) on Feb. 5, 2010[*]. The centroid was indexed by the original probe sequence IDs from the Agilent Hu25k array. The associated GenBank accession numbers for ESTs or contigs was retrieved from

[*] www.rii.com has been deprecated. See http://bioinformatics.nki.nl/data/nejm_table3.zip. Accessed on March 16, 2012.

http://www.rii.com/publications/2002/vantveer.html on Feb. 5, 2010[†]. The associated EnsEMBL transcripts retrieved from GenSigDB [35] were obtained by using a BLAST sequence similarity search, filtering the results for at least 95% similarity and at least 50% of the target sequence covered by the match to an EnsEMBL transcript.

The Affymetrix probe identifiers were mapped to the reported Agilent probe sequence IDs using the following linkers: EnsEMBL and GenBank IDs. We reported all matched probe ID(s) for individual Sequence ID in the signature obtained by each of the linkers. And for matched probes shared the same sequence ID, the aggregating procedure described above was carried out.

Among the total reported 70 sequence IDs in the 70-gene signature, 46 mapped to the Affy probes (65.7%).

Wound response gene signature

The activated-fibroblast-centroid was downloaded from the publication website (http://microarray-pubs.stanford.edu/wound_NKI/Centroids.xls). And the CSR genes in the centroid were mapped back to the original clones on Stanford cDNA array using annotation in Chang et al study[20] (http://microarray-pubs.stanford.edu/wound/Data/CSR_genes.xls). The centroid was therefore indexed by the original image clones. Since both studies used annotation with the same genome build (UniGene build 158), the accuracy of the mapped centroid was ensured.

Annotations for Stanford 43k cDNA array were retrieved from SMD SOURCE (http://smd.stanford.edu/cgi-bin/source/sourceSearch) under UniGene Build Number 222. The Affymetrix probe identifiers of the expression set were mapped to the cDNA clones using the following linkers: entrez gene ID, refseq and UniGene cluster ID. We reported all matched probe ID(s) for individual clone ID obtained by each of the linkers. And for matched probes shared the same clone ID, we selected probe(s) with maximum interquartile range (IQR: difference between the third and first quartiles). If there were still more than one hit per clone ID, the aggregating procedure described above was carried out.

Among the total reported 380 clones in the CSR signature, 298 mapped to the Affy probes (78.4%).

Hypoxia signature

The hypoxia signature[21] was developed on Stanford cDNA two-color platform. The signature was indexed by 253 unique clone IDs. Annotations for Stanford 43k cDNA array were retrieved from SMD SOURCE (http://smd.stanford.edu/cgi-bin/source/sourceSearch) under UniGene Build Number 222.

[†] www.rii.com has been deprecated. See http://bioinformatics.nki.nl/data/van-t-Veer_Nature_2002/. Accessed on March 16, 2012.

The Affymetrix probe identifiers were mapped to the cDNA clones using the following linkers: entrez gene ID, refseq and UniGene cluster ID. We reported all matched probe ID(s) for individual clone ID obtained by each of the linkers. And for matched probes shared the same clone ID, we selected probe(s) with maximum interquartile range (IQR: difference between the third and first quartiles). If there were still more than one hit per clone ID, the aggregating procedure described above was carried out.

Among the total reported 253 clones in the hypoxia signature, 117 mapped to the Affy probes (46.2%). Considering these mapped clones represented 116 unique Unigene clusters (under UniGene Build Number 222), we believed that there was a fairly good coverage for this signature on the studied data compared to the original reported size [21] (168 Unigenes).

76-gene signature

The data from the same platform as the signature, the array probe IDs were used to the map the signature to the dataset. Among the total reported 60 ER+ markers and 16 ER- markers in the 76-gene signature, all markers mapped to the studied data (100%).

Genomic Grade Index (GGI)

For data from the same platform as the signature, the array probe IDs were used to the map the signature to the new dataset. Among the total reported 16 histologic grade 1 markers and 112 histologic grade 3 markers in the GGI signature, all markers mapped to the studied dataset (100%).

Oncotype DX (21-gene Genomic Health signature)

As symbols in the signature were reported, gene symbol was used as linker for the mapping genes in the signature on the Affymetrix expression set. All markers mapped to the studied data (100%).

EndoPredict (EP signature)

As symbols in the signature were reported, gene symbol was used as linker for the mapping genes in the signature on the Affymetrix expression set. All markers mapped to the studied data (100%).

iii. Gene signatures & the associated classifers

Intrinsic gene signature & PAM50

Molecular subtype assignment

The molecular subtype signatures are carried out under a population-based assumption that is the position of a single sample in an appropriately large and heterogeneous dataset must be determined to be able to make an acute classification. We previously discussed that gene centering is an essential step in molecular subtype classifications by the existing subtype signatures[8]. In addition to that it effectively removes the technical differences between a new dataset and the original training data, it is a fundamental step to remove the differences in the general expression level of different genes and help a sample to be correctly assigned to a subtype in a heterogeneous dataset. The title SSP ("single sample predictor") on the later variants [4, 7] does not bypass the population-based characteristic of the subtype signatures.

Gene median centering was performed on the expression set, where the median of the expression values for a specific gene across all samples was subtracted from that gene. Tumors were assigned to a subtype using Pearson correlation to the expression centroids. The subtype call corresponded to the label of the centroid with the highest correlation. No threshold was set on the correlation when performing subtyping. Every tumor sample had a subtype call.

ROR score for subtype signatures

We computed the Risk-Of-Relapse (ROR) scores for tumors with both node-negative and node-positive by the Relapse risk Prediction Models described in Parker et al 2009 [7] for both subtype signatures. Originally, the risk prediction models used clinical parameters and PAM50 molecular subtypes to predict relapse for individuals. In our analysis, we applied the ROR models on the PAM50 classifications and Sørlie intrinsic signature classifications. Performances using different subtype signatures were compared. Briefly, a ROR score was assigned to each test case using correlation to the subtype alone (ROR-S) or using subtype correlation along with tumor size (ROR-C):

$$ROR\text{-}S = 0.05 \cdot basal + 0.12 \cdot HER2 - 0.34 \cdot LumA + 0.23 \cdot LumB$$
$$ROR\text{-}C = 0.05 \cdot basal + 0.11 \cdot HER2 - 0.23 \cdot LumA + 0.09 \cdot LumB + 0.17 \cdot T$$

To classify samples into specific risk groups, the thresholds were chosen from the training set in Parker et al study [7] that required no LumA sample to be in the high-risk group and no basal-like sample to be in the low-risk group. Thresholds remained unchanged when evaluating samples in this study. For ROR-S model, a sample was assigned to low risk group with a score less than -0.15; to high risk group with a score larger than 0.1; to median risk otherwise. For ROR-C model, a sample was assigned to low risk group with a score less than -0.1; to high risk group with a score larger than 0.2; to median risk otherwise.

This model was trained with a multivariable Cox model using Ridge regression fit to the node-negative, untreated subset of the van de Vijver cohort [10]. The risk models were later validated on the node positive data [36].

Measurement for agreement on subtype assignment between subtype signatures

We used Pearson correlation to measure the agreement between the two gene signatures on risk assessments by ROR-S model. Cohen's kappa coefficient *κ* [37] was used to measure the agreement for the subtype assignments:

$$\kappa = \frac{p - p_e}{1 - p_e}$$

where p is the proportion of units where there is agreement, and p_e is the proportion of units which would be expected to agree by chance. It is a form of intra-class correlation coefficient, but only values between 0 and 1 have useful meaning. A value of 1 implies perfect agreement and 0 implies no relationship.

Measurement for stability of a subtype assignment

The stability of the assignment for a specific subtype across signatures was measured by a Pearson correlation coefficient between the corresponding centroid correlations of all samples from each of signatures.

Measurement for discriminate ability of a subtype assignment

We defined a distance measurement *D* (as Delta) per sample to gauge the *discriminant ability* for individual subtypes. D per sample measured the distance between the strongest centroid correlation $\rho_{(1)}$ and the 2nd strongest correlation $\rho_{(2)}$ computed from a certain subtype signature *g*:

$$\mathrm{D}^g = \rho^g_{(1)} - \rho^g_{(2)}$$

The larger the D, the more distinguishable the assigned subtype is compared to the other subtypes. Distance measurement for intrinsic signature on sample *j* was therefore:

$$D_j^{intrinsic} = \rho_{j(1)}^{intrinsic} - \rho_{j(2)}^{intrinsic}$$

where $\rho_{j(1)}^{intrinsic}$ and $\rho_{j(2)}^{intrinsic}$ denoted as the highest and the second highest centroid correlations, respectively, in subtyping for sample *j* by intrinsic signature.

D was used in the comparison across different signatures. When the subtype calls were consistent on the same sample between intrinsic and PAM50, the distance measurement for PAM50 on sample *j* carried out similarly:

$$\mathrm{D}_j^{\mathrm{PAM50}} = \rho_{j(1)}^{\mathrm{PAM50}} - \rho_{j(2)}^{\mathrm{PAM50}}$$

However, in the case of inconsistent subtype calls on the same sample, the distance between the intrinsic signature and PAM50 on this sample was measured by the difference of the PAM50 centroid correlations between the intrinsic call (notated as *C*) and the PAM50 call:

$$\mathrm{D}_j^{\mathrm{PAM50}} = \rho_{j(C)}^{\mathrm{PAM50}} - \rho_{j(1)}^{\mathrm{PAM50}}$$

where $\rho_{j(1)}^{PAM50}$ and $\rho_{j(2)}^{PAM50}$ were the highest and the second highest centroid correlations, respectively, in PAM50 subtyping for sample *j*; while $\rho_{j(C)}^{PAM50}$ was the PAM50 centroid correlation of the call by intrinsic signature for sample *j*. A positive D score indicates consistent calling for a particular sample by the two signatures and the magnitude reflects the distinguish ability of the subtype assignment. A negative D indicates discrepant subtype callings on the same sample; the larger the absolute value, the more different of the two subtype assignments.

Measurement for agreement on subtype assignment and receptor IHC status

For Intrinsic signature (Figure 2), among 709 IHC ER-positive samples, there were 442 (62%) samples were assigned to Luminal subtypes; 22 out of 28 IHC triple-negative samples (79 %) were assigned to Basal-like subtype and 11 out of 20 IHC HER2-positive samples (55%) were assigned to HER2-enriched subtype.

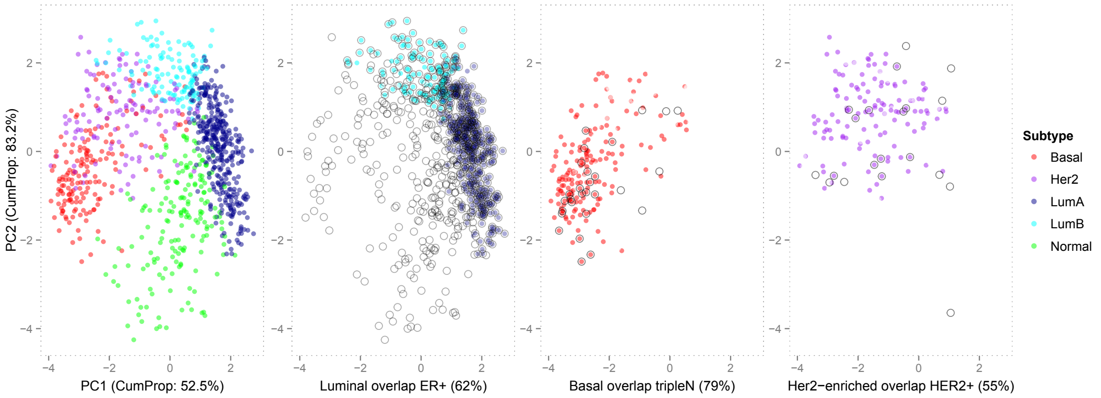

Figure 2. Overlap between molecular subtypes and receptor IHC status for Intrinsic signature subtype assignment. (Panel 1): PCA projection based on expression of all probes. Molecular subtypes were color-coded. **(Panel 2):** Luminal subtypes (solid color dots) overlap with IHC ER-positive samples (black circles). **(Panel 3):** Basal-like subtype (solid color dots) overlaps with IHC triple-negative samples (black circles) **(Panel 4):** HER2-enriched subtype (solid color dots) overlaps with IHC HER2-positive samples (black circles).

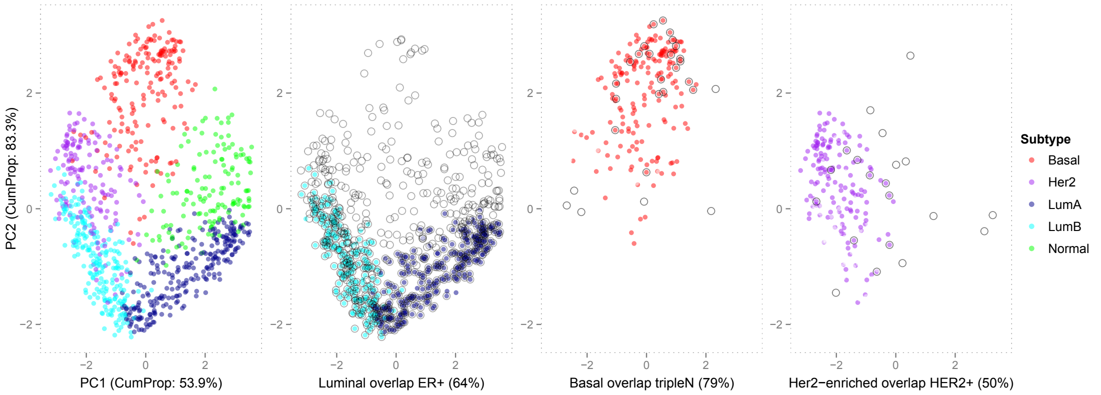

Figure 3. Overlap between molecular subtypes and receptor IHC status for PAM50 subtype assignment. Notations are the same as **Figure 2**.

For PAM50 (Figure 3), among 709 IHC ER-positive samples, there were 451 (64%) samples were assigned to Luminal subtypes; 22 out of 28 IHC triple-negative samples (79 %) were assigned to Basal-like subtype and 10 out of 20 IHC HER2-positive

samples (50%) were assigned to HER2-enriched subtype.

70-gene signature

The 70-gene prognosis profile or MammaPrint® (Agendia, Amsterdam, The Netherlands)[9] has been trained on a cohort of *lymph-node-negative* patients: expression of a set of 70 genes that was identified in a ''supervised'' fashion based on their ability to predict freedom from tumor metastasis (favorable prognosis) over a five-year period in the same dataset. It was validated subsequently on NKI295, a larger cohort consisting both node negative and positive patients[10] and another validation study[11] was done on cohorts of 241 patients with 1–3 positive lymph nodes. Despite the fact that part of the validation set in the original retrospective validation study[10] was overlapped with the training set of the signature[9], the 70-gene signature has been validated in the independent cohort by the TRANSBIG consortium[12]. Espinosa et. al[13] reproduced with quantitative reverse-transcriptase polymerase chain reaction (qRT-PCR) the results obtained with a 70-gene expression profile.

The gene signature classifies patient into good or bad prognostic group by the average profile of previously determined 70 genes in tumors from patients with a good prognosis. A patient with a correlation coefficient of more than 0.4 was then assigned to the group with a good-prognosis signature and all other patients were assigned to the group with a poor-prognosis signature. The threshold was set to achieve a 10 percent rate of false negative results in the 78 tumors in the previous study[9].

Wound response gene signature

The wound response or core serum response (CSR) gene signature[19] was derived from the transcriptional response of normal fibroblasts to serum in cell culture. It has been shown to improve the risk stratification of early breast cancer over that provided by standard clinic pathological features, in that the development of distant metastases is more likely among patients whose breast cancers have activated pathways for matrix remodeling, cell motility, and angiogenesis than among those whose cancers do not. The signature classifies tumors into two classes (*Activated* vs. *Quiescent*) through a centroid, which was built from the averaged fibroblast serum-induced expression pattern of the CSR genes[19, 20].

Computing Pearson correlation between the CSR genes expression of a tumor and the serum-activated fibroblast centroid results in a quantitative score reflecting the wound response for the tumor. The higher the correlation value, the more the sample resembles serum-activated fibroblasts ("activated" wound response signature). A negative correlation value indicates the opposite behavior and higher expression of the "quiescent" wound response signature. The threshold for the two classes can be moved up or down from zero depending on the clinical goal.

Gene expression matrix in the original studies[19, 20] was mean-centered prior to deriving the centroid. Median-centering was applied to each of the genes in expression data in this study.

Hypoxia signature

The epithelial hypoxia signature[21] consists of genes (253 image clones) that were consistently induced by hypoxia in cultured epithelial cells (HMECs and RPTECs). The 253 image clones were mapped to 168 Unigene clusters in the study [21]. A "hypoxia score" was computed for a patient by averaging expression levels for the hypoxia response genes. Patients were assigned into high or low hypoxia response group by a cutoff hypoxia-score at zero[22]. A positive score indicates *hypoxic* and nonpositive score indicates *non-hypoxic*. Using published data sets, the authors found that the "high hypoxia response" group tends to be higher grade, and more likely to have p53 and oestrogen receptor deficiencies, and, most importantly, a significant association with a poorer prognosis in breast and ovarian cancer.

The signature was downloaded from the publication website (http://microarray-pubs.stanford.edu/hypoxia/).

Genes were mean centered in the original studies [21]. We performed median centering on each of the genes in this study.

76-gene signature (Veridex)

The 76-gene signature[14] (Veridex) is designed to predict distant metastasis within 5 years for lymph-node-negative breast cancer patients. It was original developed in Wang et. al study[14] based on 286 lymph-node-negative breast cancer patients with expression profiles from Affymetrix U133a platform and validated on an independent multicentric population of 180 untreated N- breast cancer patients[15] and another gene expression study of 198 node-negative breast cancer patients[16] from the same microarray platform as in the original study[14].

Original algorithm

The 76-gene signature[14] is defined as a hierarchical model using two linear combinations of the top gene expressions with respect to a ranking based on Cox's proportional hazards model. The choice of the linear combination to compute the risk score depends on the estrogen receptor status of the patient. The gene signature consists of 60 ER+ markers and 16 ER- markers. Relapse score is calculated for ER+ and ER- samples using sum of the weighted log2-gene-expression of the 60 genes and 16 genes, respectively:

$$A + \sum_{i=1}^{60} w_i x_i \text{ (for ER positive sample)}$$

$$B + \sum_{j=1}^{16} u_j y_j \text{ (for ER negative sample)}$$

where *i* and *j* indicate markers for ER positive and ER negative group, respectively; w_i and u_j are the standardized Cox regression coefficients for ER positive and ER negative markers, respectively; x_i and y_j are the expression values in log2 scale of ER positive and ER negative markers, respectively. *A* and *B* are constants 313.5 and 280,

respectively. A negative relapse scores is defined as “good-prognosis” and positive or 0 as "poor-prognosis".

Population-based classification

Based on the procedure that derived the signature, the constants A and B in the relapse model are likely platform dependent. Ideally, applying this signature on a new dataset, one should follows the same protocol using the same platform with the same normalization procedure (log2 value; MAS5 normalized with a global mean 600).

For expression data from different platform, a more generalized population-based approach for 76-gene signature is sensible for pure prognosis purpose on the new cohort. For computing the raw relapse scores on Node negative sample, the same procedure[14] stays. However, instead of scaling by the constants A and B and further apply 0 as cutoff, the "good” prognosis is defined as less than 30% percentile of the raw relapse score in ER+ group and less than 22% percentile in ER- group. These thresholds were determined from the Desmedt et. al validation study [16], where around 30% ER positive patients were classified in the good prognosis group in while around 22% in the ER negative group was good prognosis.

Original classifier VS Population-based classifier

The Affy947 dataset consisting 147 samples in Desmedt et . al 2007[16]. Overlapping samples (n = 51) with other sets in this study were excluded from this study. For control purpose, we compared the published risk grouping calls of the 147 samples[16], which were generated on MAS5 processed dataset, with the predictions from the same samples on RMA processed data in our study. All the 147overlapping samples were classified as poor prognosis group in this study, while in Desmedt study[16] 40 samples were classified as good prognosis and 107 samples were in poor prognosis group.

The discrepancies suggested that the risk cutoffs and most likely the gene weights in the original 76-gene signature algorithm were sensitive to the data scale, which resulted from different normalization procedures given the same array platform.

Using population-based cutoffs for prognosis, we observed comparable results to the published calls of the 147 Desmedt samples.

	Population-based prognostic group in the study	
Risk assignment in Desmedt et al [16]	Good	Poor
Good	26	14
Poor	0	107

The raw relapse score was a significant predictor in a univariate Cox model for DMFS in the complete dataset (p =2.04e-11), significant in node positive group (p = 0.00527) and in node negative group (p = 5.42e-09).

Survival probabilities for distant metastasis associated with the risk groups identified using the raw relapse scores by the population-based strategy were shown in the following plot for node positive and node negative group, respectively (Figure 4).

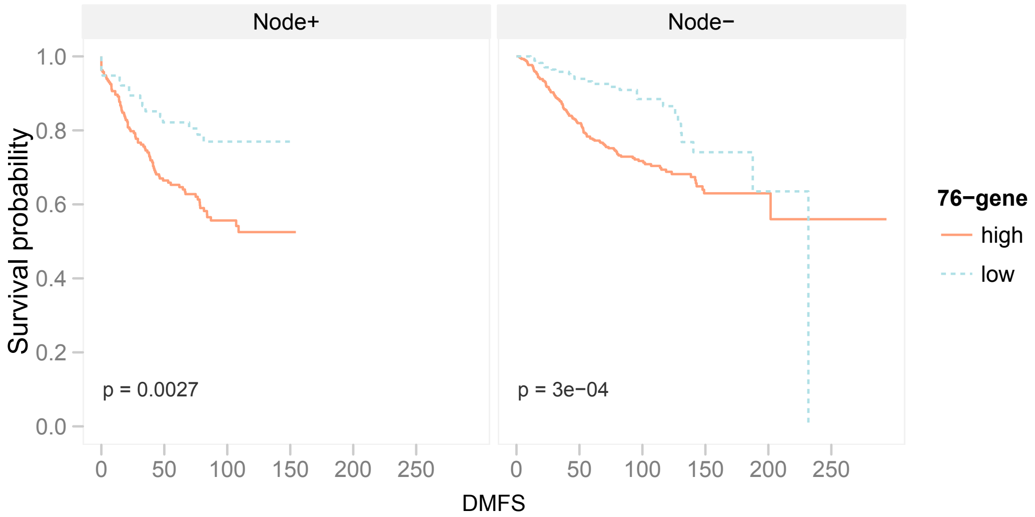

Figure 4. Kaplan meier plots for 76-gene risk groups for distant metastasis by population-based strategy in node subgroups.

Applicability on cohort with specific clinical characteristics

The 76-gene signature has been validated only on node negative cohorts[15, 16]. We did observe evidence suggesting that the signature was predictive for the node positive patients in the study ($p = 0.00527$ for continuous raw relapse score predicting DMFS in a Cox model; $p = 0.0027$ for population-based dichotomized risk groups for predicting DMFS). We applied this gene signature on the full dataset ($n = 947$) regardless of the node status.

Genomic Grade Index (GGI)

The genomic grade index (GGI) is a 97-gene measure of histologic tumor grade. The GGI was able to reclassify patients with histologic grade 2 tumors into two groups with distinct clinical outcomes similar to those of histologic grade 1 and 3, respectively[17]. High GGI is associated with decreased relapse-free survival in both untreated and tamoxifen-treated patients[18].

The GGI model consists of a linear combination of the expressions of the top ranked 128 Affymetrix probes (97 genes) according to their standardized mean difference between patients with histologic grade 1 and 3 tumors. The weights of the linear combination are simply the signs of the ranking statistics. The model was developed from data from Affymetrix U133A platform, RMA processed (with background correction, quantile normalization, and log transformation).

Because of the dependence between ER status and histologic grade; almost all ER-negative tumors were classified as either intermediate or high histologic grade, the authors[17] used only ER-positive tumors (33 histologic grade 1 tumors and the 31 histologic grade 3 tumors) for selecting the genes that were differentially expressed between histologic grade 1 and 3 tumors by a modified version of t test. Despite the

fact that the GGI genes were selected from ER positive cohort, the validation study were done on consecutive cohorts with both ER positive and ER negative tumors, as well as adjuvant systemic therapy treated and untreated patients, suggesting the usage of GGI on fairly heterogeneous breast cancer cohorts.

Original algorithm

Genomic Grade Index signature or grade-associated-97-gene signature [17] contain 128 affy probes (97 genes), of which 112 probes were with increased expression in histologic grade 3 tumors; and the remaining 16 probes with increased expression in histologic grade 1 tumors. The expressions of the 97 grade associated genes were further combined into the genomic grade index (GGI) by:

$$\text{raw GGI} = \sum_{j \in G_3} x_j - \sum_{j \in G_1} x_j$$

where x_j is the expression of either a grade 1 marker or grade 3 marker. The raw GGI scores were further scaled so that the mean of the GGI scores of histologic grade 1 tumors was − 1 and that of histologic grade 3 tumors was +1:

$$\text{GGI} = \text{scale}(\sum_{j \in G_3} x_j - \sum_{j \in G_1} x_j - \mathit{offset})$$

After scaling, tumors with negative GGI value were assigned as gene expression grade of 1 and tumors with zero or positive GGI were assigned as gene expression grade of 3.

In the original publication [17] the GGI signature was proposed to classify histologic grade 2 samples (or samples neither HG1 nor HG3) into "HG1-like" & "HG3-like". In doing so, the information of HG status are needed for the new data; the raw GGI scores are then scaled by grade status in the data with mean gene expression grade index of histologic grade 1 tumors was − 1 and that of histologic grade 3 tumors was +1. For those tumors with negative GGI score were classified as "HG1-like"; 0 or a positive GGI score put a tumor into "HG3-like" category (Figure 5).

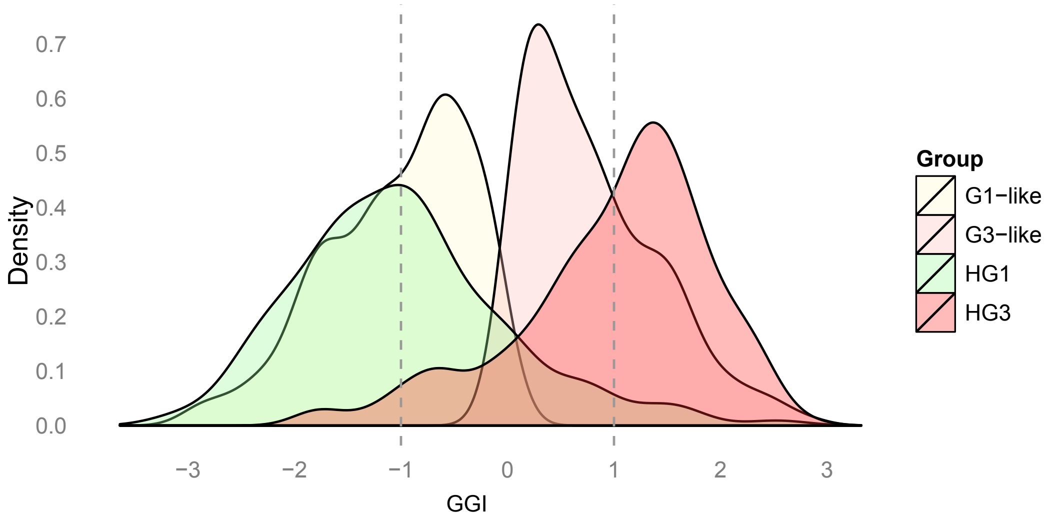

Figure 5. Distribution of the GGI scores by its classifications on Affy947 dataset. The raw GGIs were standardized so that the histologic grade 1 (HG1) group centered − 1 and histologic grade 3 (HG3) group centered +1. GGI classified HG 2 tumors into G1-like and G3-like.

Population-based classification

The cutoff based on histologic grade is suitable to discriminate HG1 and HG3-like tumors and is applicable to datasets from various sources and microarray platforms [17]. However, it may not be suitable for prognosis studies where it is usually advisable to have a small set of low-risk patients to ensure a high sensitivity. In Haibe-Kains et al study,[38] the authors dichotomized the raw GGI into "low-risk" and "high-risk" group based on 33% percentile in two populations, VDX and TRANSBIG, respectively: the third of the patients having the lowest GGI scores being defined as low-risk and the remaining patients as high-risk. The population based prognostic strategy for GGI signature particularly requires the samples are a good representative of the population of breast cancer with consecutive clinical parameter distribution.

Original classifier VS Population-based classifier

Survival probabilities of the original classifier and the population based classifier were compared in the endpoint distant metastasis (Figure 6).

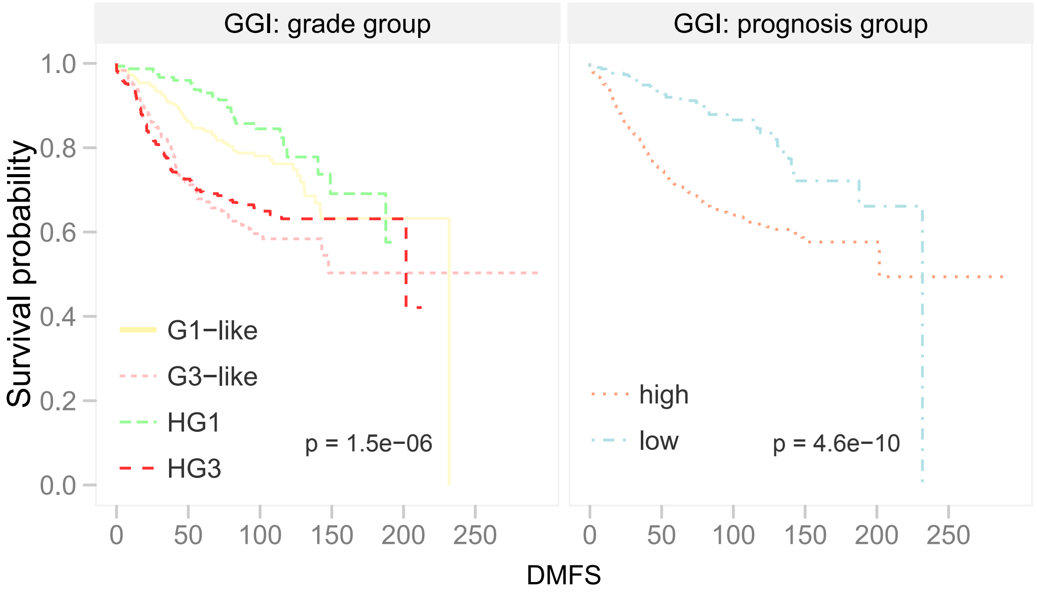

Figure 6. Kaplan meier plots for GGI risk groups for distant metastasis by the original classifier (left) and the prognosis groups using the population-based strategy (right).

Oncotype DX (21-gene Genomic Health signature)

Oncotype DX® (Genomic Health Inc., Redwood City, CA)[23] was developed specifically as a prognostic and predictive test for the benefit of chemotherapy in women with node-negative, estrogen receptor (ER)-positive breast cancer who have been treated with tamoxifen. This test is now extending its application as a predictive test for chemo therapy response in women with node-positive disease based on the results from the retrospective analysis of a trial conducted by the South Western

Oncology Group, which showed little or no benefit from chemotherapy in a low-risk node-positive patient group with tumors similar to those assessed in the node-negative setting.[24]

Original algorithm

Oncotype DX® is a 21-gene reverse transcription (RT)-PCR assay that was developed using a candidate-gene approach. It includes 16 cancer-related genes that can be grouped into five different biological domains—proliferation, HER2 signaling, ER signaling, invasion and other—along with five reference genes. The expression for each of the 16 cancer-related genes on RT-PCR assay (the number of cycles required to achieve a threshold, or Ct in triplicate, aggregated) is normalized relative to the expression of the five reference genes (ACTB, GAPDH, GUS, RPLPO, and TFRC) by subtracting the average of the expression of the reference gene. Reference-normalized expression measurements ranged from 0 to 15, where one unit increase reflects approximately a 2-fold increase in RNA, and further were linear combined into a recurrence score, RS. The score quantifies the likelihood of distant recurrence at 10 years in adjuvant-tamoxifen-treated patients with lymph node-negative, ER-positive breast cancer into categories of high risk (RS ≥ 31), intermediate risk (18 ≤ RS < 31), and low risk of recurrence (RS < 18).

To calculate the Recurrence Score, first, the scores for five different biological domains are computed as linear combination of the corresponding genes.

$$GRB7 \text{ group score } = 0.9 \times GRB7 + 0.1 \times HER2$$

$$GRB7 \text{ group score } = 8 \; if \; GRB7 \text{ group score } < 8$$

$$ER \text{ group score} = (0.8 \times ER + 1.2 \times PGR + BCL2 + SCUBE2) \div 4$$

$$\text{Proliferation group score } = (Survivin + KI67 + MYBL2 + CCNB1 + STK15) \div 5$$

$$\text{Proliferation group score} = 6.5 \; if \; \text{proliferation group score} < 6.5$$

$$\text{Invasion group score} = (CTSL2 + MMP11) \div 2$$

The unscaled RS is then computed as:

$$RSu = 0.47 \times GRB7 \; group \; score - 0.34 \times ER \; group \; score + 1.04 \times proliferation \; group \; score$$
$$+ 0.10 \times invasion \; group \; score + 0.05 \times CD68 - 0.08 \times GSTM1 - 0.07 \times BAG1$$

The RSu is further scaled into the reported Oncotype RS by:

$$RS = 0 \; if \; RSu < 0;$$

$$RS = 20 \times (RSu - 6.7) \; if \; 0 \le RSu \le 100;$$

$$RS = 100 \; if \; RSu > 100$$

Recalibration Oncotype DX® on microarray *data*

The normalized log2 ratios from the Affymetrix microarrays were rescaled to range 0 to 15, comparable to the "reference-normalized" expression in the RS calculation.[23] *Pseudo Oncotype DX® Recurrence Score* per patient was obtained by using the same grouping and coefficients used for the calculation of unscaled Recurrence Score for the Oncotype DX® assay. Due to the differences between microarray and the RT-PCR assay, applying original cutoffs for risk group identifications most likely are no longer

optimal or applicable. Instead of using cutoffs (0-18, 19-30, 31-100) to assign patients into the low, intermediate or high risk, we applied a population-based classification using the reported risk group percentages[23] where 27% patients with high score were assigned as “high risk”, and 51% with low score as “low risk”, and rest 22% patients were assigned to the “intermediate risk” group.

Original cutoffs VS Population-based cutoffs

There is moderate agreement of the identified risks groups between applying the original cutoffs and the population based approach (kappa = 0.37) on the ER+ and node- subset. Survival probabilities of the original classifier and the population-based classifier were compared in the endpoint distant metastasis (Figure 7). The continuous unscaled RS was a significant predictor in a Cox model (DMFS) in the complete set (p = 2.72e-10) and ER + group (p = 4.73e-12) but not in the ER- group (p = 0.555).

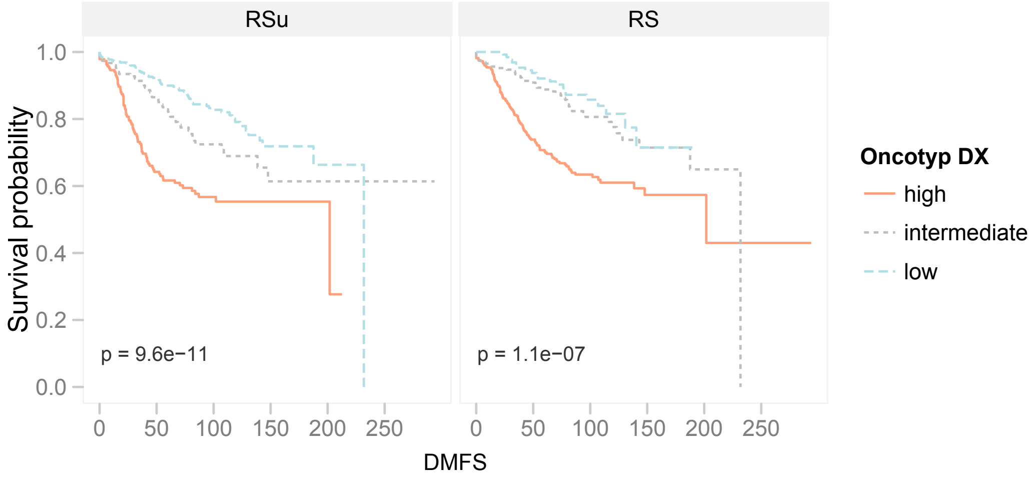

Figure 7. Kaplan meier plots for RS risk groups for distant metastasis identified by population-based strategy on unscaled Recurrence Score (RSu; left fig.) and the prognosis groups using the original classifier – applied the reported cutoffs on the scaled Recurrence Score (RS; right fig.).

Applicability on cohort with specific clinical characteristics

The Oncotype DX® applies to ER positive breast cancer patients for predicting recurrence, as no validation study has been done on ER negative cohort. Similar indications was observed on this dataset: the unscaled RS predicting of distant metastasis free survival in a Cox model for ER negative group was not significant (p = 0.555). However, we applied this gene signature on the complete set regardless of ER status to tradeoff between predictive ability and sample size.

EndoPredict

Recently, the EndoPredict [25] has been introduced as a novel qRT-PCR test for predicting the likelihood of distant recurrence in patients with ER-positive, HER2-negative breast cancer treated with adjuvant endocrine therapy only. The models were

adjusted for possible confounding covariates including ki67 and PgR. EndoPredict showed a significant added value to classic prognostic factors.

Original algorithm

The EP signature was developed using a candidate approach, and the formation of the test is quite similar to Oncotype DX®. The EP score is based on the quantification of mRNA levels of eight cancer-related genes (*BIRC5, UBE2C, DHCR7, RBBP8, IL6ST, AZGP1, MGP, and STC2*) in qRT-PCR assay, which is normalized relative to the expression of the three reference genes (*CALM2, OAZ1*, and *RPL37A*). The unscaled risk score is further computed as:

$$Su = 0.41 \times BIRC5 - 0.35 \times RBBP8 + 0.39 \times UBE2C - 0.31 \times IL6ST$$
$$- 0.26 \times AZGP1 + 0.39 \times DHCR7 - 0.18 \times MGP - 0.15 \times STC2 - 2.63$$

To avoid negative score values, the rescaled EP risk core is defined as:

$$\begin{aligned} s &= 0, && \text{if } 1.5 \cdot s_u + 18.95 < 0 \\ s &= 15, && \text{if } 1.5 \cdot s_u + 18.95 > 15 \\ s &= 1.5 \cdot s_u + 18.95, && \text{otherwise} \end{aligned}$$

Threshold for EP to discriminate patients into low and high risk of distant recurrence was set at 5.

Recalibration EP signature

Due to the differences between microarray and the RT-PCR assay, applying original cutoffs for risk group identifications most likely are no longer optimal or applicable. *Pseudo EP Score* per patient was obtained by using the same grouping and coefficients used for the calculation of unscaled risk score *Su* for EP. Instead of using cutoff 5 to assign patients into the low or high risk on the scaled risk score *s*, we applied a population-based classification using the reported risk group percentages [39] (also based on personal commutation with the original author) where 49% patients with low score were assigned as “low risk”, and rest patients were assigned to the “high risk” group.

Original cutoffs VS Population-based cutoffs

Applying the reported cutoff on the scaled risk score s, all samples were classified in the high risk group. Although the genes in EP algorithm were those tend to have highly correlated expression levels between PCR and microarray, the original training set was MAS5 processed Affymetrix dataset. Most likely, the reported coefficients and the cutoffs are not optimal for the RMA processed data in our study.

Meanwhile, using the population-based strategy on all samples (n = 947), 49% patient (n = 464) was assigned to low risk group and the rest 483 patients were classified as high risk.

Applicability on cohort with specific clinical characteristics

The EndoPredict was originally designed for ER-positive, HER2-negative breast cancer patients for predicting distant recurrence, as no validation study has been done on ER negative or HER2-postive cohort. The unscaled risk score *Su* predicting of distant metastasis free survival in a univariate Cox model on the complete dataset (n= 912, HR = 1.33, p = 4.3e-11) was comparable to the prediction for ER-positive, HER2-negative sub group (n= 627, HR = 1.46, p = 4.9e-11). In addition, the probabilities for distant metastasis associated with individual risk groups from the population-based strategy were significantly separated on the complete set, the ER-positive, HER2-negative subset, the ER-positive, HER2-negative treated and untreated subset, respectively (Figure 8). In our study, we applied the population-based strategy for this gene signature on the complete set (n = 912) regardless of ER and HER2 status.

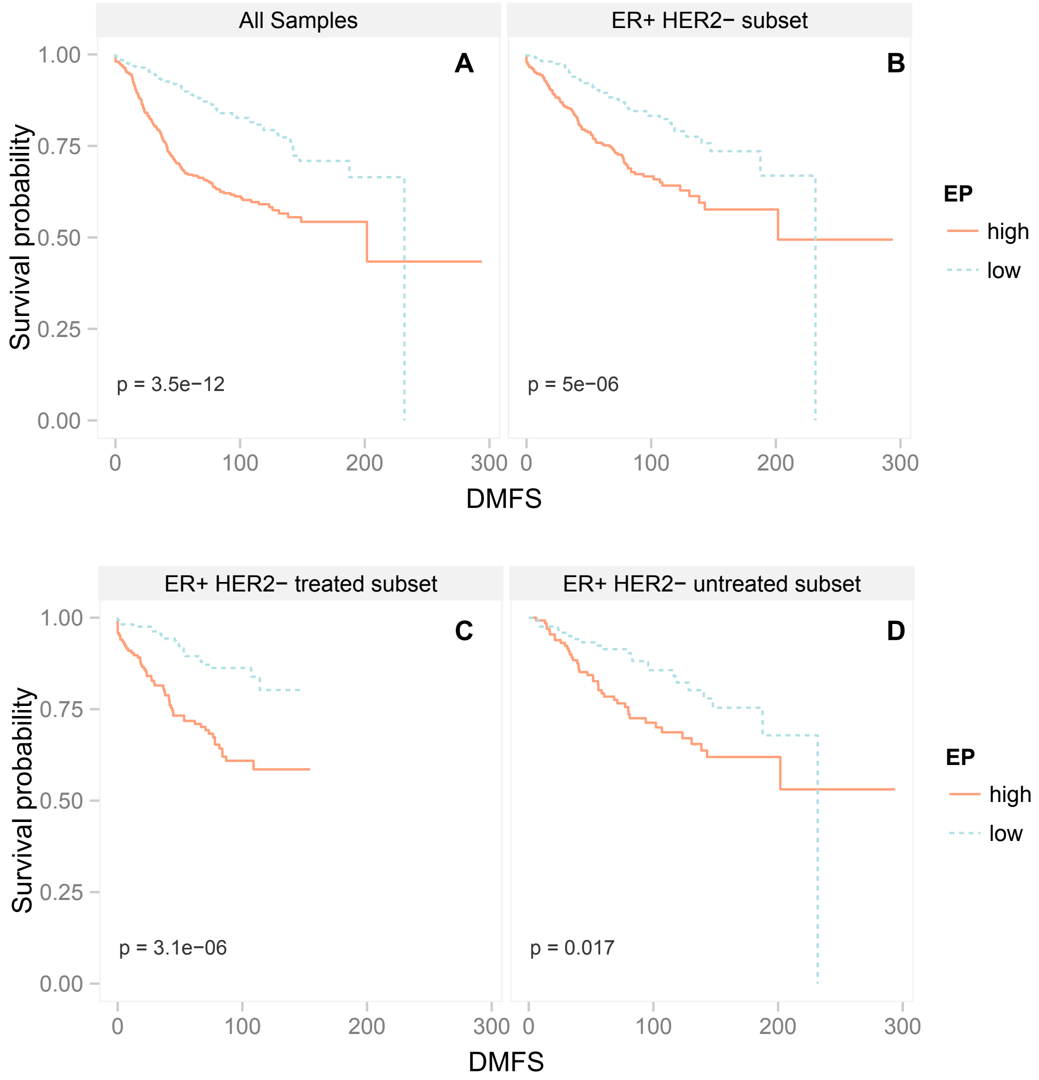

Figure 8. Kaplan meier plots for EP risk groups for distant metastasis identified by population-based strategy on unscaled risk score on the completed dataset (A; n = 912), ER-positive, HER2-negative subgroup (B; n = 627), ER-positive, HER2-negative treated with systemic treatment subgroup (C; n = 331) and ER-positive, HER2-negative not treated with systemic treatment subgroup (D; n = 254).

iv. Statistical analysis

Model evaluation: concordance index (C-index)

The concordance index (C-index)[40] is a widely accepted measurement for predictive discrimination of a given model. In survival analysis, it is a generalization of the area under the receiver operating characteristic (ROC) curve. The C-index is defined as the probability that risk assignments to members of a random pair are accurately ranked according to their prognosis. It measures the probability of concordance between the predicted and observed responses in terms of lengths of survival of any two patients:

$$C - index = \frac{\sum_{i,j \in \Omega} 1\{r_i > r_j\}}{|\Omega|}$$

where r_i and r_j are the predicted risk for *i*th and *j*th patient; respectively. Ω is a set of all possible pairs of patients, at least one of whom has experienced an event and time to event $t_i < t_j$. If the predicted risk is larger for the patient who lived shorter, the predictions for that pair are said to be concordant with the outcomes. The number of concordant pairs (order of failure and risk assignment agree), discordant pairs (order of failure and risk assignment disagree), and uninformative pairs are tabulated to calculate the measure. The C-index is ranging from 0 to 1. Values of 0.5 indicate random prediction and higher values indicate increasing prediction accuracy.

Variability in the C-index for each predictor and P values from comparisons were estimated from 1,000 bootstrap samples of the risk assignments. Calculation was done using the rcorrcens and rcorr.cens function implemented in the Hmisc (19) library in R statistical analysis environment[41] version 2.12.2.

Model evaluation: proportion of variation explained (PVE)

Comparable with the R^2 in regression modeling, the importance of covariates in the Cox model can be quantified using the proportion of variation explained in the outcome variable (PVE) [42] by one or more covariates:

$$R_M^2 = 1 - \left(L_R / L_U\right)^{2/n}$$

$$L_R \text{ : likelihood of a model without covariates } \left(\text{restricted}\right)$$

$$L_U \text{ : likelihood of a model with covariates } \left(\text{unrestricted}\right)$$

where *n* denotes total sample size. The relative importance of a covariate in a multivariate Cox model was measured by the partial PVE, which was calculated as the different of R_M^2 for the full model and R_M^2 for a model with a factor of interest excluded.

Time-dependent effect estimation

A univariate Cox model taking individual gene signature as predictor and ER as stratification covariate was fitted. Tests for the proportional-hazards assumption were performed by correlating the scaled Schoenfeld residuals with Kaplan-Meier estimates[43]. The scaled Schoenfeld residual is the difference between the covariate at the failure time and the expected value of the covariate at this time. When the proportional-hazards assumption is satisfied, the scaled Schoenfeld residuals should be evenly distributed around 0 along the time span; any systematic pattern would indicate non-constant effect of the covariate over time. The function cox.zph in the R library survival was used.

In order to unveil the nature of the non-proportional deviation, we used the additive regression model, namely Aalen's additive nonparametric model [44, 45] to investigate the time-dependent effect of *j*th gene signature for survival prediction by

$$h(t) = h_0(t) + \beta_j(t)x_j$$

where x_j is the risk score from the *j*th gene signature and $h_0(t)$ is the baseline hazard function, while the β_j *(t)* is the increase in the hazard at time *t* corresponding to a unit´s increase in the *j*th gene signature (covariate). The parameter β_j *(t)* is an arbitrary regression function and is assumed to impact additively upon the (unknown) baseline hazard, allowing the effect to change in magnitude and even sign with time. Continuous risk scores from each of the original gene signatures were used as numeric covariate in a univariate additive regression model. The covariate was mean-centered prior to estimating the cumulative baseline hazard. The additive model was fitted using function addreg implemented in R (http://www.med.uio.no/imb/stat/addreg/beta/Addreg-beta.html).

Our interpretations of the cumulative regression function estimator from an additive model were focused on the slope of the estimated curve and its evolvement along timeline, which reflected the effect of a specific gene signature changing over time for survival prediction. The additive model gives an appealing understanding of how the hazard profile of a gene signature is distributed. However, the cumulative regression functions do not easily transform into a single numerical estimate of the covariate effect. We then reply on Cox model within time interval to quantify the *relative risk* or *hazard ratio* (HR) for 1-unit-increase in risk prediction by a specific gene signature. The *Hazard Ratio* (HR) is usually an accuracy measure for the risk group prediction for categorical predictors. The larger the HR, the better is the discrimination between the groups of the patients, such as low- and high-risk. In our study, continuous covariates entering the Cox models were scaled into mean 0 with standard deviation 1. Thus the estimated HR on the standardized data characterized the relative risk for 1-standard-deviation increase in risk prediction by a gene signature.

Software

All analyses were performed in R statistical analysis platform.[41] The R plotting system "ggplot2"[46] was to used for visualization.

III. Supplementary Discussion on Signature construction strategies

Methods based on centroid correlations (e.g. subtype signatures, 70-gene and WR) and methods that transform the data into an invariant scale before computing the risk scores (e.g. GGI) have more consistent performances across different studies. We suspect that summarizing expression pattern by weighted average fashion (e.g. 76-gene, RS and Hypoxia) is more sensitive to the data scale and the issue of missing signature-gene(s) in the data at hand.

For the 76-gene signature, the pre-derived constants in the relapse model are likely platform-dependent. Additionally, we observed that this signature was unable to identify any Desmedt[16] sample with good prognosis when applied on RMA- instead of MAS5-normalized data. The discrepancies suggested that the risk cutoffs and possibly its original gene weights in the algorithm are sensitive to the data scale. Similarly for the RS, the reference-normalized expression measurements in this PCR-derived test ranged from 0 to 15, where one unit increase reflects approximately a 2-fold increase in RNA. Such exact quantification is less feasible in microarray-based measurements. Although GGI shares similarities with these two signatures in constructing risk estimation from gene expression pattern, it has a unique standardization procedure incorporating the information of histological grade, which likely increases its robustness when transferred to different microarray platforms.

Generally, the originally derived cutoffs for risk group assignment often become less optimal in a new study. The population-based strategy is more general and applicable for a study with pure prognosis purpose on the new cohort. However, it particularly requires the samples are a good representative of the population of breast cancer with consecutive clinical parameter distribution.

IV. Supplementary Results

Multivariate analysis on gene signature

With presence of clinical parameters

We tested prognostic power of the gene signatures after adjusting tumor size, lymph node status and histological grade. Information added by gene signature on top of clinical parameter was tested using analysis of deviance by comparing the following Cox models fitted with (model 2) or without the signature covariate:

Model 1: Risk = Clinical parameter + strata(ER)
Model 2: Risk = Signature + Clinical parameter + strata(ER)

Information added by clinical parameter on top of signature was tested by comparing Cox models fitted with (model 2) or without the clinical parameter (model 3):

Model 3: Risk = Signature + strata(ER)

Models were fitted on the same complete dataset with available information for endpoint and the tested parameters ($n = 760$) and for the entire follow-up time.

Gene signatures added significant information to tumor size (Box 1), node (Box 2) and histological grade (Box 3), respectively. Histological grade did not contribute additional prediction power to most of the gene signatures (Box 4), except for Intrinsic ($p = 0.017$) and Hypoxia ($p = 0.001$).

Analysis on dataset source as potential confounding factor

The data used in this study was pulled from six published studies, to ensure the observations from the study is not cofounded by dataset effect, we included the dataset source as a factor in a multivariate Cox model together with gene signature:

Risk = Signature + Dataset + strata(ER)

Models were fitted on the full dataset ($n = 912$) and for the entire follow-up time. To see if dataset source added significant amount of information towards prognosis on top on gene signature, analysis of deviance was carried out on deduction of deviance due to adding covariate Dataset. We did not observe significant effect associated with "Dataset" for any studied gene signatures (Box 5(A)).

We also examined dataset effect separately in ER+ group and ER- group using a multivariate Cox model:

Risk = Signature + Dataset

We did not observe significant effect associated with "Dataset" for any studied gene signatures for the ER+ group (Box 5(B)) nor ER- group (Box 5(C)).

Box 1: Analysis of Deviance for information added by signature to Size

```
# Results for Intrinsic
Cox model: response is  Surv(t_dmfs, e_dmfs)
 Model 1: ~ factor(TS) + strata(ER_IHC_expr)
 Model 2: ~ Intrinsic_RORs + factor(TS) + strata(ER_IHC_expr)
   loglik  Chisq Df P(>|Chi|)
1 -1134.2
2 -1129.5 9.4153  1  0.002152 **

# Results for PAM50
 Cox model: response is  Surv(t_dmfs, e_dmfs)
 Model 1: ~ factor(TS) + strata(ER_IHC_expr)
 Model 2: ~ PAM50_RORs + factor(TS) + strata(ER_IHC_expr)
   loglik  Chisq Df P(>|Chi|)
1 -1134.2
2 -1115.2 38.015  1 7.019e-10 ***

# Results for 70-gene
 Cox model: response is  Surv(t_dmfs, e_dmfs)
 Model 1: ~ factor(TS) + strata(ER_IHC_expr)
 Model 2: ~ gene70 + factor(TS) + strata(ER_IHC_expr)
   loglik  Chisq Df P(>|Chi|)
1 -1134.2
2 -1122.6 23.378  1 1.331e-06 ***

# Results for 76-gene
 Cox model: response is  Surv(t_dmfs, e_dmfs)
 Model 1: ~ factor(TS) + strata(ER_IHC_expr)
 Model 2: ~ gene76 + factor(TS) + strata(ER_IHC_expr)
   loglik  Chisq Df P(>|Chi|)
1 -1134.2
2 -1116.7 35.087  1 3.152e-09 ***

# Results for GGI
 Cox model: response is  Surv(t_dmfs, e_dmfs)
 Model 1: ~ factor(TS) + strata(ER_IHC_expr)
 Model 2: ~ GGI + factor(TS) + strata(ER_IHC_expr)
   loglik  Chisq Df P(>|Chi|)
1 -1134.2
2 -1116.4 35.764  1 2.227e-09 ***


# Results for WR
 Cox model: response is  Surv(t_dmfs, e_dmfs)
 Model 1: ~ factor(TS) + strata(ER_IHC_expr)
 Model 2: ~ WR + factor(TS) + strata(ER_IHC_expr)
   loglik  Chisq Df P(>|Chi|)
1 -1134.2
2 -1119.2 30.177  1 3.943e-08 ***

# Results for Hypoxia
 Cox model: response is  Surv(t_dmfs, e_dmfs)
 Model 1: ~ factor(TS) + strata(ER_IHC_expr)
 Model 2: ~ Hypoxia + factor(TS) + strata(ER_IHC_expr)
   loglik  Chisq Df P(>|Chi|)
1 -1134.2
2 -1133.5 1.6024  1    0.2056

# Results for RS
 Cox model: response is  Surv(t_dmfs, e_dmfs)
 Model 1: ~ factor(TS) + strata(ER_IHC_expr)
 Model 2: ~ RS + factor(TS) + strata(ER_IHC_expr)
   loglik  Chisq Df P(>|Chi|)
1 -1134.2
2 -1119.8 28.883  1 7.688e-08 ***

# Results for EP
 Cox model: response is  Surv(t_dmfs, e_dmfs)
 Model 1: ~ factor(TS) + strata(ER_IHC_expr)
 Model 2: ~ EP + factor(TS) + strata(ER_IHC_expr)
   loglik Chisq Df P(>|Chi|)
1 -1134.2
2 -1115.8 36.97  1   1.2e-09 ***
```

Box 2: Analysis of Deviance for information added by signature to Node

```
# Results for Intrinsic
 Cox model: response is  Surv(t_dmfs, e_dmfs)
 Model 1: ~ factor(Node) + strata(ER_IHC_expr)
 Model 2: ~ Intrinsic_RORs + factor(Node) + strata(ER_IHC_expr)
   loglik  Chisq Df P(>|Chi|)
1 -1138.2
2 -1132.9 10.554  1   0.00116 **

# Results for PAM50
 Cox model: response is  Surv(t_dmfs, e_dmfs)
 Model 1: ~ factor(Node) + strata(ER_IHC_expr)
 Model 2: ~ PAM50_RORs + factor(Node) + strata(ER_IHC_expr)
   loglik  Chisq Df P(>|Chi|)
1 -1138.2
2 -1118.6 39.188  1  3.85e-10 ***

# Results for 70-gene
 Cox model: response is  Surv(t_dmfs, e_dmfs)
 Model 1: ~ factor(Node) + strata(ER_IHC_expr)
 Model 2: ~ gene70 + factor(Node) + strata(ER_IHC_expr)
   loglik Chisq Df P(>|Chi|)
1 -1138.2
2 -1127.5 21.25  1 4.032e-06 ***

# Results for 76-gene
 Cox model: response is  Surv(t_dmfs, e_dmfs)
 Model 1: ~ factor(Node) + strata(ER_IHC_expr)
 Model 2: ~ gene76 + factor(Node) + strata(ER_IHC_expr)
   loglik  Chisq Df P(>|Chi|)
1 -1138.2
2 -1120.3 35.617  1 2.401e-09 ***

# Results for GGI
Cox model: response is  Surv(t_dmfs, e_dmfs)
 Model 1: ~ factor(Node) + strata(ER_IHC_expr)
 Model 2: ~ GGI + factor(Node) + strata(ER_IHC_expr)
   loglik  Chisq Df P(>|Chi|)
1 -1138.2
2 -1119.3 37.793  1 7.865e-10 ***

# Results for WR
 Cox model: response is  Surv(t_dmfs, e_dmfs)
 Model 1: ~ factor(Node) + strata(ER_IHC_expr)
 Model 2: ~ WR + factor(Node) + strata(ER_IHC_expr)
   loglik  Chisq Df P(>|Chi|)
1 -1138.2
2 -1118.9 38.508  1 5.453e-10 ***

# Results for Hypoxia
Cox model: response is  Surv(t_dmfs, e_dmfs)
 Model 1: ~ factor(Node) + strata(ER_IHC_expr)
 Model 2: ~ Hypoxia + factor(Node) + strata(ER_IHC_expr)
   loglik  Chisq Df P(>|Chi|)
1 -1138.2
2 -1137.4 1.5649  1     0.211

# Results for RS
 Cox model: response is  Surv(t_dmfs, e_dmfs)
 Model 1: ~ factor(Node) + strata(ER_IHC_expr)
 Model 2: ~ RS + factor(Node) + strata(ER_IHC_expr)
   loglik  Chisq Df P(>|Chi|)
1 -1138.2
2 -1122.1 32.174  1  1.41e-08 ***

# Results for EP
 Cox model: response is  Surv(t_dmfs, e_dmfs)
 Model 1: ~ factor(Node) + strata(ER_IHC_expr)
 Model 2: ~ EP + factor(Node) + strata(ER_IHC_expr)
   loglik  Chisq Df P(>|Chi|)
1 -1138.2
2 -1118.2 39.983  1 2.561e-10 ***
```

Box 3: Analysis of Deviance for information added by signature to Grade

```
# Results for Intrinsic
Cox model: response is  Surv(t_dmfs, e_dmfs)
 Model 1: ~ factor(Grade) + strata(ER_IHC_expr)
 Model 2: ~ Intrinsic_RORs + factor(Grade) + strata(ER_IHC_expr)
   loglik  Chisq Df P(>|Chi|)
1 -1142.3
2 -1139.4 5.9159  1   0.01501 *

# Results for PAM50
 Cox model: response is  Surv(t_dmfs, e_dmfs)
 Model 1: ~ factor(Grade) + strata(ER_IHC_expr)
 Model 2: ~ PAM50_RORs + factor(Grade) + strata(ER_IHC_expr)
   loglik  Chisq Df P(>|Chi|)
1 -1142.3
2 -1124.8 35.108  1 3.119e-09 ***

# Results for 70-gene
 Cox model: response is  Surv(t_dmfs, e_dmfs)
 Model 1: ~ factor(Grade) + strata(ER_IHC_expr)
 Model 2: ~ gene70 + factor(Grade) + strata(ER_IHC_expr)
   loglik  Chisq Df P(>|Chi|)
1 -1142.3
2 -1133.2 18.198  1 1.991e-05 ***

# Results for 76-gene
 Cox model: response is  Surv(t_dmfs, e_dmfs)
 Model 1: ~ factor(Grade) + strata(ER_IHC_expr)
 Model 2: ~ gene76 + factor(Grade) + strata(ER_IHC_expr)
   loglik  Chisq Df P(>|Chi|)
1 -1142.3
2 -1126.5 31.657  1 1.839e-08 ***

# Results for GGI
 Cox model: response is  Surv(t_dmfs, e_dmfs)
 Model 1: ~ factor(Grade) + strata(ER_IHC_expr)
 Model 2: ~ GGI + factor(Grade) + strata(ER_IHC_expr)
   loglik  Chisq Df P(>|Chi|)
1 -1142.3
2 -1125.4 33.803  1 6.097e-09 ***

# Results for WR
 Cox model: response is  Surv(t_dmfs, e_dmfs)
 Model 1: ~ factor(Grade) + strata(ER_IHC_expr)
 Model 2: ~ WR + factor(Grade) + strata(ER_IHC_expr)
   loglik Chisq Df P(>|Chi|)
1 -1142.3
2 -1127.5  29.7  1 5.045e-08 ***

# Results for Hypoxia
 Cox model: response is  Surv(t_dmfs, e_dmfs)
 Model 1: ~ factor(Grade) + strata(ER_IHC_expr)
 Model 2: ~ Hypoxia + factor(Grade) + strata(ER_IHC_expr)
   loglik  Chisq Df P(>|Chi|)
1 -1142.3
2 -1141.7 1.3663  1    0.2424

# Results for RS
 Cox model: response is  Surv(t_dmfs, e_dmfs)
 Model 1: ~ factor(Grade) + strata(ER_IHC_expr)
 Model 2: ~ RS + factor(Grade) + strata(ER_IHC_expr)
   loglik  Chisq Df P(>|Chi|)
1 -1142.3
2 -1128.5 27.726  1 1.398e-07 ***

# Results for EP
 Cox model: response is  Surv(t_dmfs, e_dmfs)
 Model 1: ~ factor(Grade) + strata(ER_IHC_expr)
 Model 2: ~ EP + factor(Grade) + strata(ER_IHC_expr)
   loglik  Chisq Df P(>|Chi|)
1 -1142.3
2 -1124.8 35.085  1 3.156e-09 ***
```

Box 4: Analysis of Deviance for information added by Grade to signature

```
# Results for Intrinsic
 Cox model: response is  Surv(t_dmfs, e_dmfs)
 Model 1: ~ Intrinsic_RORs + strata(ER_IHC_expr)
 Model 2: ~ Intrinsic_RORs + factor(Grade) + strata(ER_IHC_expr)
   loglik  Chisq Df P(>|Chi|)
1 -1143.5
2 -1139.4 8.1368  2   0.01711 *

# Results for PAM50
 Cox model: response is  Surv(t_dmfs, e_dmfs)
 Model 1: ~ PAM50_RORs + strata(ER_IHC_expr)
 Model 2: ~ PAM50_RORs + factor(Grade) + strata(ER_IHC_expr)
   loglik  Chisq Df P(>|Chi|)
1 -1126.3
2 -1124.8 3.0159  2    0.2214

# Results for 70-gene
 Cox model: response is  Surv(t_dmfs, e_dmfs)
 Model 1: ~ gene70 + strata(ER_IHC_expr)
 Model 2: ~ gene70 + factor(Grade) + strata(ER_IHC_expr)
   loglik  Chisq Df P(>|Chi|)
1 -1136.4
2 -1133.2 6.2753  2   0.04339 *

# Results for 76-gene
 Cox model: response is  Surv(t_dmfs, e_dmfs)
 Model 1: ~ gene76 + strata(ER_IHC_expr)
 Model 2: ~ gene76 + factor(Grade) + strata(ER_IHC_expr)
   loglik  Chisq Df P(>|Chi|)
1 -1127.9
2 -1126.5 2.6922  2    0.2603

# Results for GGI
 Cox model: response is  Surv(t_dmfs, e_dmfs)
 Model 1: ~ GGI + strata(ER_IHC_expr)
 Model 2: ~ GGI + factor(Grade) + strata(ER_IHC_expr)
   loglik  Chisq Df P(>|Chi|)
1 -1127.7
2 -1125.4 4.5252  2    0.1041

# Results for WR
 Cox model: response is  Surv(t_dmfs, e_dmfs)
 Model 1: ~ WR + strata(ER_IHC_expr)
 Model 2: ~ WR + factor(Grade) + strata(ER_IHC_expr)
   loglik  Chisq Df P(>|Chi|)
1 -1129.6
2 -1127.5 4.1341  2    0.1266

# Results for Hypoxia
 Cox model: response is  Surv(t_dmfs, e_dmfs)
 Model 1: ~ Hypoxia + strata(ER_IHC_expr)
 Model 2: ~ Hypoxia + factor(Grade) + strata(ER_IHC_expr)
   loglik  Chisq Df P(>|Chi|)
1 -1148.7
2 -1141.7 14.067  2 0.0008817 ***

# Results for RS
 Cox model: response is  Surv(t_dmfs, e_dmfs)
 Model 1: ~ RS + strata(ER_IHC_expr)
 Model 2: ~ RS + factor(Grade) + strata(ER_IHC_expr)
   loglik  Chisq Df P(>|Chi|)
1 -1130.8
2 -1128.5 4.6782  2   0.09641 .

# Results for EP
 Cox model: response is  Surv(t_dmfs, e_dmfs)
 Model 1: ~ EP + strata(ER_IHC_expr)
 Model 2: ~ EP + factor(Grade) + strata(ER_IHC_expr)
   loglik  Chisq Df P(>|Chi|)
1 -1126.4
2 -1124.8 3.1582  2    0.2062
```

Box 5: Analysis of Deviance for model with individual gene signature & dataset as covariates:
(A) In ER-stratified cox model

```
Analysis of Deviance Table
Cox model: response is Surv(t_dmfs, e_dmfs)
Terms added sequentially (first to last)

# Results for Intrinsic
                 loglik  Chisq Df Pr(>|Chi|)
NULL            -1442.5
Intrinsic_RORs  -1437.7 9.5240  1   0.002028 **
factor(Dataset) -1434.4 6.5953  5   0.252521

# Results for PAM50
                 loglik   Chisq Df Pr(>|Chi|)
NULL            -1442.5
PAM50_RORs      -1417.3 50.2752  1  1.336e-12 ***
factor(Dataset) -1413.7  7.1689  5     0.2084

# Results for 70-gene
                 loglik   Chisq Df Pr(>|Chi|)
NULL            -1442.5
gene70          -1432.1 20.7018  1  5.367e-06 ***
factor(Dataset) -1428.6  7.0919  5     0.2139

# Results for 76-gene
                 loglik  Chisq Df Pr(>|Chi|)
NULL            -1442.5
gene76          -1419.8 45.315  1  1.677e-11 ***
factor(Dataset) -1416.7  6.301  5      0.278

# Results for GGI
                 loglik   Chisq Df Pr(>|Chi|)
NULL            -1442.5
GGI             -1421.6 41.7951  1  1.014e-10 ***
factor(Dataset) -1418.1  6.8889  5      0.229

# Results for WR
                 loglik  Chisq Df Pr(>|Chi|)
NULL            -1442.5
WR              -1422.0 40.852  1  1.642e-10 ***
factor(Dataset) -1417.3  9.506  5     0.0905 .

# Results for Hypoxia
                 loglik  Chisq Df Pr(>|Chi|)
NULL            -1442.5
Hypoxia         -1440.1 4.7104  1    0.02998 *
factor(Dataset) -1437.2 5.8519  5    0.32090

# Results for RS
                 loglik   Chisq Df Pr(>|Chi|)
NULL            -1442.5
RS              -1425.2 34.4446  1  4.386e-09 ***
factor(Dataset) -1421.6  7.3237  5     0.1977

# Results for EP
                 loglik   Chisq Df Pr(>|Chi|)
NULL            -1442.5
EP              -1422.0 40.9558  1  1.557e-10 ***
factor(Dataset) -1419.0  5.9849  5     0.3077
```

Box 5: Analysis of Deviance for model with individual gene signature & dataset as covariates:
(B) In ER+ group

```
Analysis of Deviance Table
Cox model: response is Surv(t_dmfs, e_dmfs)
Terms added sequentially (first to last)

# Results for Intrinsic
                 loglik  Chisq Df Pr(>|Chi|)
NULL             -1078.3
Intrinsic_RORs   -1073.4 9.7678  1   0.001776 **
factor(Dataset)  -1070.0 6.8858  5   0.229275

# Results for PAM50
                 loglik   Chisq Df Pr(>|Chi|)
NULL             -1078.3
PAM50_RORs       -1049.3 57.9666  1  2.665e-14 ***
factor(Dataset)  -1046.4  5.9327  5     0.3128

# Results for 70-gene
                 loglik   Chisq Df Pr(>|Chi|)
NULL             -1078.3
gene70           -1062.7 31.2201  1  2.304e-08 ***
factor(Dataset)  -1059.0  7.4325  5     0.1904

# Results for 76-gene
                 loglik   Chisq Df Pr(>|Chi|)
NULL             -1078.3
gene76           -1056.9 42.8605  1  5.879e-11 ***
factor(Dataset)  -1053.1  7.5285  5     0.1842

# Results for GGI
                 loglik   Chisq Df Pr(>|Chi|)
NULL             -1078.3
GGI              -1050.3 56.0692  1  6.994e-14 ***
factor(Dataset)  -1047.4  5.8061  5     0.3255

# Results for WR
                 loglik  Chisq Df Pr(>|Chi|)
NULL             -1078.3
WR               -1055.0 46.545  1  8.955e-12 ***
factor(Dataset)  -1050.0 10.058  5    0.07361 .

# Results for Hypoxia
                 loglik  Chisq Df Pr(>|Chi|)
NULL             -1078.3
Hypoxia          -1078.2 0.1256  1      0.723
factor(Dataset)  -1074.1 8.3706  5      0.137

# Results for RS
                 loglik   Chisq Df Pr(>|Chi|)
NULL             -1078.3
RS               -1057.1 42.4384  1  7.294e-11 ***
factor(Dataset)  -1054.3  5.5636  5      0.351

# Results for EP
                 loglik   Chisq Df Pr(>|Chi|)
NULL             -1078.3
EP               -1053.5 49.5324  1  1.951e-12 ***
factor(Dataset)  -1051.1  4.9521  5     0.4218
```

Box 5: Analysis of Deviance for model with individual gene signature & dataset as covariates:
(C) In ER- group

```
Analysis of Deviance Table
Cox model: response is Surv(t_dmfs, e_dmfs)
Terms added sequentially (first to last)

# Results for Intrinsic
                 loglik  Chisq Df Pr(>|Chi|)
NULL            -364.14
Intrinsic_RORs  -363.88 0.5204  1     0.4707
factor(Dataset) -362.17 3.4243  5     0.6349

# Results for PAM50
                 loglik  Chisq Df Pr(>|Chi|)
NULL            -364.14
PAM50_RORs      -364.00 0.2851  1     0.5934
factor(Dataset) -362.21 3.5646  5     0.6136

# Results for 70-gene
                 loglik  Chisq Df Pr(>|Chi|)
NULL            -364.14
gene70          -363.71 0.8638  1     0.3527
factor(Dataset) -361.97 3.4811  5     0.6262

# Results for 76-gene
                 loglik  Chisq Df Pr(>|Chi|)
NULL            -364.14
gene76          -362.45 3.3739  1    0.06624 .
factor(Dataset) -361.28 2.3482  5    0.79916

# Results for GGI
                 loglik  Chisq Df Pr(>|Chi|)
NULL            -364.14
GGI             -364.13 0.0249  1     0.8747
factor(Dataset) -362.46 3.3234  5     0.6503

# Results for WR
                 loglik  Chisq Df Pr(>|Chi|)
NULL            -364.14
WR              -363.47 1.3316  1     0.2485
factor(Dataset) -361.49 3.9615  5     0.5550

# Results for Hypoxia
                 loglik  Chisq Df Pr(>|Chi|)
NULL            -364.14
Hypoxia         -359.65 8.9698  1   0.002745 **
factor(Dataset) -358.44 2.4233  5   0.787997

# Results for RS
                 loglik   Chisq Df Pr(>|Chi|)
NULL            -364.14
RS              -363.96 0.3500  1     0.5541
factor(Dataset) -362.14 3.6496  5     0.6009

# Results for EP
                 loglik  Chisq Df Pr(>|Chi|)
NULL            -364.14
EP              -364.12 0.0423  1     0.8371
factor(Dataset) -362.43 3.3839  5     0.6410
```

V. Reference

1. Perou CM, Sørlie T, Eisen MB, van de Rijn M, Jeffrey SS, Rees CA, et al. Molecular portraits of human breast tumours. Nature. 2000; **406**(6797): 747-52.
2. Sørlie T, Perou CM, Tibshirani R, Aas T, Geisler S, Johnsen H, et al. Gene expression patterns of breast carcinomas distinguish tumor subclasses with clinical implications. Proc Natl Acad Sci U S A. 2001; **98**(19): 10869-74.
3. Sørlie T, Tibshirani R, Parker J, Hastie T, Marron JS, Nobel A, et al. Repeated observation of breast tumor subtypes in independent gene expression data sets. Proc Natl Acad Sci U S A. 2003; **100**(14): 8418-23.
4. Hu Z, Fan C, Oh DS, Marron JS, He X, Qaqish BF, et al. The molecular portraits of breast tumors are conserved across microarray platforms. BMC Genomics. 2006; **7**: 96.
5. Perreard L, Fan C, Quackenbush JF, Mullins M, Gauthier NP, Nelson E, et al. Classification and risk stratification of invasive breast carcinomas using a real-time quantitative RT-PCR assay. Breast Cancer Res. 2006; **8**(2): R23.
6. Perou CM, Jeffrey SS, van de Rijn M, Rees CA, Eisen MB, Ross DT, et al. Distinctive gene expression patterns in human mammary epithelial cells and breast cancers. Proceedings of the National Academy of Sciences. 1999; **96**(16): 9212-7.
7. Parker JS, Mullins M, Cheang MCU, Leung S, Voduc D, Vickery T, et al. Supervised risk predictor of breast cancer based on intrinsic subtypes. Journal of Clinical Oncology. 2009; **27**(8): 1160.
8. Sørlie T, Borgan E, Myhre S, Vollan HK, Russnes H, Zhao X, et al. The importance of gene-centring microarray data. The Lancet Oncology. 2010; **11**(8): 719-20.
9. van 't Veer LJ, Dai H, van de Vijver MJ, He YD, Hart AA, Mao M, et al. Gene expression profiling predicts clinical outcome of breast cancer. Nature. 2002; **415**(6871): 530-6.
10. van de Vijver MJ, He YD, van't Veer LJ, Dai H, Hart AA, Voskuil DW, et al. A gene-expression signature as a predictor of survival in breast cancer. N Engl J Med. 2002; **347**(25): 1999-2009.
11. Mook S, Schmidt MK, Viale G, Pruneri G, Eekhout I, Floore A, et al. The 70-gene prognosis-signature predicts disease outcome in breast cancer patients with 1-3 positive lymph nodes in an independent validation study. Breast Cancer Res Treat. 2008.
12. Buyse M, Loi S, Van't Veer L, Viale G, Delorenzi M, Glas AM, et al. Validation and clinical utility of a 70-gene prognostic signature for women with node-negative breast cancer. JNCI Cancer Spectrum. 2006; **98**(17): 1183.
13. Espinosa E, Vara J, Redondo A, Sanchez J, Hardisson D, Zamora P, et al. Breast cancer prognosis determined by gene expression profiling: a quantitative reverse transcriptase polymerase chain reaction study. Journal of Clinical Oncology. 2005; **23**(29): 7278.
14. Wang Y, Klijn JG, Zhang Y, Sieuwerts AM, Look MP, Yang F, et al. Gene-expression profiles to predict distant metastasis of lymph-node-negative primary breast cancer. Lancet. 2005; **365**(9460): 671-9.
15. Foekens JA, Atkins D, Zhang Y, Sweep FCGJ, Harbeck N, Paradiso A, et al. Multicenter validation of a gene expression-based prognostic signature in lymph node-negative primary breast cancer. Journal of Clinical Oncology. 2006; **24**(11): 1665.
16. Desmedt C, Piette F, Loi S, Wang Y, Lallemand F, Haibe-Kains B, et al. TRANSBIG Consortium. Strong time dependence of the 76-gene prognostic signature for node-negative breast cancer patients in the TRANSBIG multicenter independent validation series. Clin Cancer Res. 2007; **13**(11): 3207-14.
17. Sotiriou C, Wirapati P, Loi S, Harris A, Fox S, Smeds J, et al. Gene expression profiling in breast cancer: understanding the molecular basis of histologic grade to improve prognosis. J Natl Cancer Inst. 2006; **98**(4): 262-72.
18. Loi S, Haibe-Kains B, Desmedt C, Lallemand F, Tutt AM, Gillet C, et al. Definition of clinically distinct molecular subtypes in estrogen receptor-positive breast carcinomas through genomic grade. Journal of Clinical Oncology. 2007; **25**(10): 1239.
19. Chang HY, Sneddon JB, Alizadeh AA, Sood R, West RB, Montgomery K, et al. Gene expression signature of fibroblast serum response predicts human cancer progression: similarities between tumors and wounds. PLoS Biol. 2004; **2**(2): E7.
20. Chang HY, Nuyten DSA, Sneddon JB, Hastie T, Tibshirani R, Sørlie T, et al. Robustness, scalability, and integration of a wound-response gene expression signature in predicting breast cancer

survival. Proceedings of the National Academy of Sciences of the United States of America. 2005; **102**(10): 3738.
21. Chi JT, Wang Z, Nuyten DS, Rodriguez EH, Schaner ME, Salim A, et al. Gene expression programs in response to hypoxia: cell type specificity and prognostic significance in human cancers. PLoS Med. 2006; **3**(3): e47.
22. Nuyten DSA, Hastie T, Chi JTA, Chang HY, van de Vijver MJ. Combining biological gene expression signatures in predicting outcome in breast cancer: An alternative to supervised classification. European Journal of Cancer. 2008; **44**(15): 2319-29.
23. Paik S, Shak S, Tang G, Kim C, Baker J, Cronin M, et al. A multigene assay to predict recurrence of tamoxifen-treated, node-negative breast cancer. N Engl J Med. 2004; **351**(27): 2817-26.
24. Albain KS, Barlow WE, Shak S, Hortobagyi GN, Livingston RB, Yeh I. Prognostic and predictive value of the 21-gene recurrence score assay in postmenopausal women with node-positive, oestrogen-receptor-positive breast cancer on chemotherapy: a retrospective analysis of a randomised trial. The Lancet Oncology. 2010; **11**(1): 55-65.
25. Filipits M, Rudas M, Jakesz R, Dubsky P, Fitzal F, Singer CF, et al. A new molecular predictor of distant recurrence in ER-positive, HER2-negative breast cancer adds independent information to conventional clinical risk factors. Clinical Cancer Research. 2011; **17**(18): 6012-20.
26. Miller LD, Smeds J, George J, Vega VB, Vergara L, Ploner A, et al. An expression signature for p53 status in human breast cancer predicts mutation status, transcriptional effects, and patient survival. Proceedings of the National Academy of Sciences of the United States of America. 2005; **102**(38): 13550.
27. Pawitan Y, Bjöhle J, Amler L, Borg AL, Egyhazi S, Hall P, et al. Gene expression profiling spares early breast cancer patients from adjuvant therapy: derived and validated in two population-based cohorts. Breast Cancer Research. 2005; **7**(6): R953-R64.
28. Minn AJ, Gupta GP, Siegel PM, Bos PD, Shu W, Giri DD, et al. Genes that mediate breast cancer metastasis to lung. Nature. 2005; **436**(7050): 518-24.
29. Chin K, DeVries S, Fridlyand J, Spellman PT, Roydasgupta R, Kuo WL, et al. Genomic and transcriptional aberrations linked to breast cancer pathophysiologies. Cancer Cell. 2006; **10**(6): 529-41.
30. van Vliet MH, Reyal F, Horlings HM, van de Vijver MJ, Reinders MJ, Wessels LF. Pooling breast cancer datasets has a synergetic effect on classification performance and improves signature stability. BMC Genomics. 2008; **9**: 375.
31. Bolstad B, Collin F, Brettschneider J, Simpson K, Cope L, Irizarry R, et al. Quality assessment of Affymetrix GeneChip data. Bioinformatics and computational biology solutions using R and bioconductor. 2005: 33-47.
32. Irizarry RA, Bolstad BM, Collin F, Cope LM, Hobbs B, Speed TP. Summaries of Affymetrix GeneChip probe level data. Nucleic acids research. 2003; **31**(4): e15.
33. Gentleman RC, Carey VJ, Bates DM, Bolstad B, Dettling M, Dudoit S, et al. Bioconductor: open software development for computational biology and bioinformatics. Genome biology. 2004; **5**(10): R80.
34. Durinck S, Moreau Y, Kasprzyk A, Davis S, De Moor B, Brazma A, et al. BioMart and Bioconductor: a powerful link between biological databases and microarray data analysis. Bioinformatics. 2005; **21**(16): 3439.
35. Culhane AC, Schwarzl T, Sultana R, Picard KC, Picard SC, Lu TH, et al. GeneSigDB--a curated database of gene expression signatures. Nucleic acids research. 2009.
36. Nielsen TO, Parker JS, Leung S, Voduc KD, Ebbert M, Vickery TL, et al. A comparison of PAM50 intrinsic subtyping with immunohistochemistry and clinical prognostic factors in tamoxifen-treated estrogen receptor positive breast cancer. Clinical Cancer Research. 2010.
37. Cohen J. A coefficient of agreement for nominal scales. Educational and psychological measurement. 1960; **20**(1): 37-46.
38. Haibe-Kains B, Desmedt C, Piette F, Buyse M, Cardoso F, Van't Veer L, et al. Comparison of prognostic gene expression signatures for breast cancer. BMC Genomics. 2008; **9**(1): 394.
39. Dubsky P, Filipits M, Jakesz R, Rudas M, Singer C, Greil R, et al. EndoPredict improves the prognostic classification derived from common clinical guidelines in ER-positive, HER2-negative early breast cancer. Annals of oncology. 2012.
40. Harrell F, Lee K, Mark D. Tutorial in biostatistics multivariable prognostic models: issues in developing models, evaluating assumptions and adequacy, and measuring and reducing errors. Statistics in medicine. 1996; **15**: 361-87.
41. R Development Core Team. R: A language and environment for statistical computing. R Foundation for Statistical Computing. 2011.

42. Schemper M. The relative importance of prognostic factors in studies of survival. Statistics in medicine. 1993; **12**(24): 2377-82.
43. Grambsch PM, Therneau TM. Proportional hazards tests and diagnostics based on weighted residuals. Biometrika. 1994; **81**(3): 515.
44. Aalen OO. A linear regression model for the analysis of life times. Statistics in medicine. 1989; **8**(8): 907-25.
45. Aalen OO, Borgan Ø, Gjessing HK. Survival and event history analysis: a process point of view: Springer Verlag; 2008.
46. Wickham H. ggplot2: elegant graphics for data analysis: Springer-Verlag New York Inc; 2009.
