# Supplementary material for: Systematic assessment of prognostic gene signatures for breast cancer shows distinct influence of time and ER status
Source: BMC Cancer. 2014 Mar 19;14:211. doi: 10.1186/1471-2407-14-211 (PMC4000128; doi:10.1186/1471-2407-14-211)
Supplement: Additional file 3 — Sweave file containing reproducible report for Zhao et al. [file 1471-2407-14-211-S3.pdf]

Reproducible report for Zhao et al.: Systematic assessment of prognostic gene signatures for breast cancer shows distinct influence of time and ER status

Xi Zhao[1]

[1] *Center for Cancer Systems Biology, Department of Radiology, School of Medicine, Stanford University, Stanford. USA*

February 26, 2014

Contents

1 Dataset **2**

1.1 Affy947 2

1.2 METABRIC 4

2 Gene signatures **5**

3 Predicting risk scores by gene signatures for Affy947 dataset **7**

4 Analysis **11**

4.1 Similarity on risk prediction across gene signatures 11

4.2 Time & ER dependency of gene signatures' prognostic effects . . 11

5 Data repository **16**

6 Contact Information **16**

7 Session Info **17**

This document is written to accompany "Systematic assessment of prognostic gene signatures for breast cancer shows distinct influence of time and ER status" by Xi Zhao, Einar Andreas Rødland, Therese Sørlie, Hans Kristian Moen Vollan, Hege G. Russnes, Vessela N Kristensen, Ole Christian Lingjærde and Anne-Lise Børresen-Dale. This document was generated from Rnw (or Sweave) file. All the R code contained herein is evaluable and can be used to recreate the results described in Zhao et al. All the scripts and data from the study are stored in the R data file *GeneSig.RData*.

```
load("GeneSig.RData")
```

1 Dataset

1.1 Affy947

The gene expression dataset (van Vliet et al., 2008) (n=947) is a collection of six published breast cancer microarray datasets (Chin et al., 2006; Desmedt et al., 2007; Loi et al., 2007; Miller et al., 2005; Minn et al., 2005; Pawitan et al., 2005) on Affymetrix Human Genome HG-U133A arrays. A total of 947 gene expression profiles is available in this dataset. Preprocessed and normalised gene expression data, together with the clinical information on the 947 samples are stored in ExpressionSet object `Affy947`.

```
Affy947

## ExpressionSet (storageMode: lockedEnvironment)
## assayData: 22268 features, 947 samples
##   element names: exprs
## protocolData: none
## phenoData
##   sampleNames: des_001 des_002 ... chin_123 (947 total)
##   varLabels: Arrays_ID Sample_ID ... HER2_IHC_expr (66 total)
##   varMetadata: labelDescription
## featureData: none
## experimentData: use 'experimentData(object)'
## Annotation: hgu133a

str(pData(Affy947))

## 'data.frame': 947 obs. of  66 variables:
##  $ Arrays_ID                                  : chr  "GSM177885" "GSM177886" "GSM177887"
##  $ Sample_ID                                  : chr  "VDXGUYU_4002" "VDXGUYU_4008" "VDXG
##  $ set_1                                      : chr  "desmedt" "desmedt" "desmedt" "desm
##  $ set_2                                      : chr  "GUY" "GUY" "GUY" "GUY" ...
##  $ Age                                        : int  57 57 48 42 46 58 44 58 47 38 ...
##  $ t_rfs                                      : num  23.3 5.9 16.9 70.7 123.3 ...
##  $ e_rfs                                      : int  1 1 1 1 1 0 1 1 0 1 ...
##  $ t_dmfs                                     : num  23.3 212.6 16.9 201.8 123.3 ...
##  $ e_dmfs                                     : int  1 0 1 1 1 0 0 1 0 1 ...
```

```
##  $ t_os                                       : num  30.2 212.6 29.7 201.8 133.3 ...
##  $ e_os                                       : int  1 0 1 1 1 0 0 1 0 1 ...
##  $ t_sos                                      : num  NA NA NA NA NA NA NA NA NA NA ...
##  $ e_sos                                      : int  NA NA NA NA NA NA NA NA NA NA ...
##  $ Histtype                                   : int  1 2 1 1 1 2 1 2 2 2 ...
##  $ Angioinv                                   : int  1 1 0 1 1 1 1 1 2 2 ...
##  $ Lymp_infil                                 : int  2 3 2 3 2 2 3 2 NA 3 ...
##  $ Size                                       : num  30 30 25 18 30 20 20 25 30 25 ...
##  $ Grade                                      : int  3 3 3 3 2 2 3 1 3 2 ...
##  $ ER                                         : int  0 1 0 1 1 1 0 1 1 1 ...
##  $ PGR                                        : int  NA NA NA NA NA NA NA NA NA NA ...
##  $ Node                                       : int  0 0 0 0 0 0 0 0 0 0 ...
##  $ p53_seq_mut_status_1mutant_0wt             : int  NA NA NA NA NA NA NA NA NA NA ...
##  $ p53_DLDA_classifier_result_0wtlike_1mtlike: int  NA NA NA NA NA NA NA NA NA NA ...
##  $ DLDA_error_1yes_0no                        : int  NA NA NA NA NA NA NA NA NA NA ...
##  $ p53mut_seq_paw                             : int  NA NA NA NA NA NA NA NA NA NA ...
##  $ p53_mutlike_paw                            : int  NA NA NA NA NA NA NA NA NA NA ...
##  $ Erbb2_ihc                                  : int  NA NA NA NA NA NA NA NA NA NA ...
##  $ p53_ihc                                    : int  NA NA NA NA NA NA NA NA NA NA ...
##  $ ki67                                       : logi  NA NA NA NA NA NA ...
##  $ risksg                                     : int  2 2 2 2 2 2 2 2 2 2 ...
##  $ NPI                                        : num  4.6 4.6 4.5 4.36 3.6 3.4 4.4 2.5 4.
##  $ risknpi                                    : int  2 2 2 2 2 2 2 1 2 2 ...
##  $ AOL_os_10y                                 : num  62.7 69 66.2 84.9 80.2 83.1 80.6 82
##  $ risk_AOL                                   : int  1 1 1 1 1 1 1 1 1 1 ...
##  $ veridex_risk                               : chr  "Poor" "Poor" "Poor" "Poor" ...
##  $ Wang                                       : num  28.5 169 51 55.8 112.7 ...
##  $ Wang_bin                                   : int  1 1 1 1 1 0 1 0 1 1 ...
##  $ GGI                                        : num  0.626 1.84 0.396 1.168 -0.129 ...
##  $ GGI_bin                                    : int  1 1 1 1 0 0 1 0 0 1 ...
##  $ IGS                                        : num  0.0707 0.4416 0.1736 0.4849 -0.2091
##  $ IGS_bin                                    : int  1 1 1 1 0 1 1 0 1 0 ...
##  $ RSU                                        : num  16.33 9.99 17.08 8.94 9.44 ...
##  $ RS                                         : num  100 65.7 100 44.8 54.8 ...
##  $ Rsbin                                      : int  2 2 2 2 2 2 2 0 0 2 ...
##  $ CSR                                        : num  0.0192 0.2545 0.0631 0.0889 -0.0232
##  $ CSR_bin                                    : int  1 1 1 1 1 0 1 0 1 1 ...
##  $ LumB_HU                                    : num  0.0248 0.3456 -0.3397 0.3547 0.3304
##  $ LumA_HU                                    : num  -0.217 -0.123 -0.473 -0.213 0.152 .
##  $ Normal_HU                                  : num  -0.1764 -0.4332 0.0845 -0.2693 -0.0
##  $ Basal_HU                                   : num  0.0972 -0.0281 0.5953 0.0685 -0.220
##  $ Her2_HU                                    : num  0.15 0.136 0.065 0.263 0.063 ...
##  $ max                                        : num  0.15 0.346 0.595 0.355 0.33 ...
##  $ which_max                                  : int  5 1 4 1 1 2 4 3 2 3 ...
##  $ CIN70                                      : num  9.81 54.02 10.18 27.43 -4.68 ...
##  $ CIN25                                      : num  1.49 22.3 4.43 13.15 -2.79 ...
##  $ CIN70_bin                                  : int  1 1 1 1 0 0 1 0 0 1 ...
##  $ CIN25_bin                                  : int  1 1 1 1 0 0 1 0 0 1 ...
##  $ ER_IHC                                     : int  0 1 0 1 1 1 0 1 1 1 ...
```

```
##  $ ER_205225_at                                 : num  -3.264 0.327 -3.264 0.644 0.379 ...
##  $ ER_bin_205225_at                             : int  0 1 0 1 1 1 0 1 1 1 ...
##  $ HER_216836_S_at                              : num  -0.866 -1.013 -0.738 -0.352 -0.358
##  $ HER_bin_216836_S_at                          : int  0 0 0 0 0 0 0 0 0 0 ...
##  $ EE                                           : int  3 3 3 3 2 2 3 1 3 2 ...
##  $ ER_IHC_expr                                  : int  0 1 0 1 1 1 0 1 1 1 ...
##  $ SystemicTreatment                            : int  0 0 0 0 0 0 0 0 0 0 ...
##  $ HER2_IHC_expr                                : int  0 0 0 0 0 0 0 0 0 0 ...
```

1.2 METABRIC

METABRIC (Curtis et al., 2012) expression discovery set was used in Zhao et al. as an additional evaluation. A total of 996 gene expression profiles and clinical information are in ExpressionSet object `METABRIC_training_996`.

```
METABRIC_training_996

## ExpressionSet (storageMode: lockedEnvironment)
## assayData: 48803 features, 996 samples
##   element names: exprs
## protocolData: none
## phenoData
##   sampleNames: MB.0002 MB.0008 ... MB.5654 (996 total)
##   varLabels: age_at_diagnosis group ... Grade (17 total)
##   varMetadata: labelDescription
## featureData: none
## experimentData: use 'experimentData(object)'
## Annotation: illuminaHumanv3.db

str(pData(METABRIC_training_996))

## 'data.frame': 996 obs. of  17 variables:
##  $ age_at_diagnosis    : num  43.2 77 78.8 84.2 85.5 ...
##  $ group               : int  4 4 4 4 4 4 4 4 4 4 ...
##  $ grade               : int  3 3 3 2 2 2 3 3 2 2 ...
##  $ size                : int  10 40 31 28 22 33 17 23 16 34 ...
##  $ lymph_nodes_positive: chr  "neg" "pos" "neg" "neg" ...
##  $ histological_type   : chr  "IDC" "IDC" "IDC" "ILC" ...
##  $ ER_IHC_status       : chr  "pos" "pos" "pos" "neg" ...
##  $ cellularity         : chr  "high" "high" "moderate" "high" ...
##  $ Pam50Subtype        : chr  "LumA" "LumB" "LumB" "Her2" ...
##  $ Treatment           : chr  "HT/RT" "CT/HT/RT" "HT/RT" "NONE" ...
##  $ Site                : int  1 1 1 1 1 1 1 1 1 1 ...
##  $ time                : num  49.5 41.4 7.8 36.3 77.1 ...
##  $ event               : num  0 1 1 1 0 0 0 0 0 0 ...
##  $ ER_IHC_expr         : num  1 1 1 0 1 1 1 1 1 1 ...
##  $ Node                : num  0 1 0 0 0 1 0 0 1 0 ...
##  $ Size                : int  10 40 31 28 22 33 17 23 16 34 ...
##  $ Grade               : int  3 3 3 2 2 2 3 3 2 2 ...
```

To recreate the main analysis in Zhao et al., we use the Affy947 dataset in this document for evaluating prediction of Distant Metastasis Free Survival (DMFS) by gene signatures.

```
X = Affy947
x = exprs(X)
para.t = "t_dmfs"
para.e = "e_dmfs"
```

2 Gene signatures

Nine gene signatures were included in the study: Intrinsic signature, PAM50, 70-gene signature, 76-gene signature, Genomic Grade Index (GGI), Wound response (WR), Hypoxia, 21-gene-Recurrence-Score (RS), EndoPredict (EP). They are stored in the following R objects: `Intrinsic`, `Pam50`, `Gene70`, `Gene76`, `GGI`, `WR` ,`Hypoxia` ,`OncotypeDX` ,`EndoPredict`. Each of the signature R objects contains information essential for the gene signatures such as the published centroids (e.g. `Intrinsic`, `Pam50`, `Gene70`, `WR`) or associated classifier (e.g.`Gene76`, `GGI`), the type of platform where the signature was originally developed from and geneID annotation.

```
str(Intrinsic)

## List of 4
##  $ GeneSignature:'data.frame': 549 obs. of  5 variables:
##   ..$ LumA  : num [1:549] -0.0176 1.2072 0.2045 0.8868 -0.5632 ...
##   ..$ LumB  : num [1:549] 0.2708 -0.3287 -0.18 -0.0905 0.1803 ...
##   ..$ Her2  : num [1:549] 0.844 0.38 -0.141 -0.21 0.21 ...
##   ..$ Basal : num [1:549] 0.3076 -0.4682 0.0299 -0.2879 0.1528 ...
##   ..$ Normal: num [1:549] -0.365 -0.319 -0.133 0.072 -0.78 ...
##  $ platform     : chr "Stanford44kcDNA"
##  $ Annotation   :'data.frame': 549 obs. of  4 variables:
##   ..$ CloneID  : chr [1:549] "IMAGE:1031045" "IMAGE:1031076" "IMAGE:108422" "IMAGE:1086
##   ..$ UGCluster: chr [1:549] "Hs.1176" "Hs.98428" NA "Hs.603766" ...
##   ..$ GeneID   : chr [1:549] "6508" "3216" NA "23389" ...
##   ..$ UGRepAcc : chr [1:549] "AY142112" "AK292200" NA "AY338463" ...
##  $ subtypeCode  :'data.frame': 5 obs. of  2 variables:
##   ..$ level: chr [1:5] "Basal" "Her2" "LumA" "LumB" ...
##   ..$ color: chr [1:5] "red" "purple" "darkblue" "cyan" ...

str(Pam50)

## List of 3
##  $ GeneSignature:'data.frame': 50 obs. of  5 variables:
##   ..$ Basal : num [1:50] 0.718 0.537 -0.575 -0.119 0.3 ...
##   ..$ Her2  : num [1:50] -0.482 0.267 -0.476 -0.158 0.406 ...
##   ..$ LumA  : num [1:50] 0.00998 -0.57925 0.75822 0.28749 -0.88143 ...
##   ..$ LumB  : num [1:50] -0.1906 0.0988 -0.4055 -0.4413 0.6039 ...
##   ..$ Normal: num [1:50] 0.466 -0.837 0.317 0.534 -0.877 ...
```

```
##  $ platform     : chr "AgilentExpr44k_2channel"
##  $ subtypeCode  :'data.frame': 5 obs. of  2 variables:
##   ..$ level: chr [1:5] "Basal" "Her2" "LumA" "LumB" ...
##   ..$ color: chr [1:5] "red" "purple" "darkblue" "cyan" ...
```

```
str(Gene70)
```

```
## List of 3
##  $ GeneSignature:'data.frame': 70 obs. of  1 variable:
##   ..$ GoodPrognosis: num [1:70] -5.162 -1.441 -0.938 -1.499 -1.284 ...
##  $ platform     : chr "Rossetta AgilentHu25k"
##  $ Annotation   :'data.frame': 70 obs. of  5 variables:
##   ..$ Sequence     : chr [1:70] "AL080059" "Contig63649_RC" "Contig46218_RC" "NM_016359
##   ..$ GoodPrognosis: num [1:70] -5.162 -1.441 -0.938 -1.499 -1.284 ...
##   ..$ GenBank      : chr [1:70] "AL080059" "AW014921" "AI813331" "NM_016359" ...
##   ..$ EnsemblID    : chr [1:70] "ENSG00000180543" "" "ENSG00000139734" "ENSG00000137804
##   ..$ CLID         : chr [1:70] "IMAGE:745011" "" "" "IMAGE:951241" ...
```

```
str(Gene76)
```

```
## List of 2
##  $ GeneSignature:'data.frame': 76 obs. of  3 variables:
##   ..$ Standard.Cox.coef: num [1:76] -3.83 -3.87 3.63 -3.47 3.51 ...
##   ..$ Cox_p_val        : num [1:76] 0.00005 0.00001 0.00002 0.00016 0.00008 0.00001 0.0
##   ..$ ER               : int [1:76] 1 1 1 1 1 1 1 1 1 1 ...
##  $ platform     : chr "AffyHGu133a"
```

```
str(GGI)
```

```
## List of 3
##  $ GeneSignature:'data.frame': 128 obs. of  1 variable:
##   ..$ grade: int [1:128] 3 3 3 3 3 3 3 3 3 3 ...
##  $ platform     : chr "AffymetrixU133A"
##  $ Annotation   :'data.frame': 128 obs. of  9 variables:
##   ..$ probeID           : chr [1:128] "201088_at" "201090_x_at" "201195_s_at" "201475_
##   ..$ grade             : int [1:128] 3 3 3 3 3 3 3 3 3 3 ...
##   ..$ GeneBankID        : chr [1:128] "NM_002266" "NM_006082" "AB018009" "NM_004990" .
##   ..$ EntrezGeneID      : int [1:128] 3838 NA 8140 4141 7334 10212 4605 NA 332 332 ...
##   ..$ NCBI.GeneSymbol   : chr [1:128] "KPNA2" "" "SLC7A5" "MARS" ...
##   ..$ HUGO.GeneSymbol   : chr [1:128] "KPNA2" "" "SLC7A5" "MARS" ...
##   ..$ Cytoband          : chr [1:128] "17q23.1-q23.3" "" "16q24.3" "12q13.2" ...
##   ..$ Alternative.symbols: chr [1:128] "IPOA1|QIP2|RCH1|SRP1alpha" "" "4F2LC|CD98|D16S4
##   ..$ Description       : chr [1:128] "karyopherin alpha 2 (RAG cohort 1, importin alp
```

```
str(WR)
```

```
## List of 3
##  $ GeneSignature:'data.frame': 380 obs. of  1 variable:
##   ..$ Activated_Fibroblast_centroid_value: num [1:380] -0.398 -0.462 0.513 -0.394 -0.42
##  $ platform     : chr "Stanford44kcDNA"
##  $ Annotation   :'data.frame': 380 obs. of  4 variables:
```

```
##   ..$ CloneID  : chr [1:380] "IMAGE:50276" "IMAGE:271855" "IMAGE:812088" "IMAGE:502586"
##   ..$ UGCluster: chr [1:380] "Hs.195642" "Hs.46850" "Hs.247460" "Hs.490795" ...
##   ..$ GeneID   : chr [1:380] "57674" "79158" "57486" "57488" ...
##   ..$ UGRepAcc : chr [1:380] "NM_020914" "AM085438" "AB033052" "NM_020728" ...
```

```
str(Hypoxia)
```

```
## List of 3
##  $ GeneSignature: chr [1:253] "IMAGE:436065" "IMAGE:204569" "IMAGE:795178" "IMAGE:16231
##  $ platform     : chr "Stanford44kcDNA"
##  $ Annotation   :'data.frame': 253 obs. of  4 variables:
##   ..$ CloneID  : chr [1:253] "IMAGE:436065" "IMAGE:204569" "IMAGE:795178" "IMAGE:162310
##   ..$ UGCluster: chr [1:253] "Hs.687693" "Hs.176626" "Hs.654377" "Hs.372031" ...
##   ..$ GeneID   : chr [1:253] NA "55363" "3948" "5376" ...
##   ..$ UGRepAcc : chr [1:253] "AW979248" "AY244805" "BC064388" "NM_000304" ...
```

```
str(OncotypeDX)
```

```
## List of 2
##  $ GeneSignature:'data.frame': 21 obs. of  4 variables:
##   ..$ published_symbol      : chr [1:21] "Ki67" "STK15" "Survivin" "CCNB1" ...
##   ..$ published_accessionNum: chr [1:21] "" "" "" "" ...
##   ..$ AffyHGu133a           : chr [1:21] "" "" "" "" ...
##   ..$ group                 : chr [1:21] "Proliferation" "Proliferation" "Proliferation
##  $ platform     : chr "Taqman RT-PCR"
```

```
str(EndoPredict)
```

```
## List of 2
##  $ GeneSignature:'data.frame': 11 obs. of  4 variables:
##   ..$ published_symbol      : chr [1:11] "BIRC5" "UBE2C" "DHCR7" "RBBP8" ...
##   ..$ published_accessionNum: logi [1:11] NA NA NA NA NA NA ...
##   ..$ AffyHGu133a           : logi [1:11] NA NA NA NA NA NA ...
##   ..$ group                 : chr [1:11] "GOI" "GOI" "GOI" "GOI" ...
##  $ platform     : chr "Taqman RT-PCR"
```

3 Predicting risk scores by gene signatures for Affy947 dataset

On Affy947 dataset , the predicted risk scores by PAM50 can be computed using function `pam50.symbol2affy()`. It takes the expression matrix `x` as input and applies the nearest centroid classification to generate molecular subtype calls for each of the Affy947 samples. The subtype calls are futher used by the ROR model (Parker et al. 2009) to caculate the risk scores. The conversion of the gene identifies from gene symbols to Affymetrix U133a probeIDs is carried out by an internal function `symbol2affyhgu133a()` through R library *biomaRt*.

```
out.pam50 = pam50.symbol2affy(x, UCSC_hg18 = T)

## #----------- Pam50 gene signature (Parker et al 2009) ----------#
## >> Classify data from Affy U133a expresstion platform ...
## >> Extracting matched PAM50 gene expression matrix ...
## cross-platform mapping coverage: 42/50=84%
## >> Gene median centering ...
## >> Calibrating subtypes for single sample ...
## >> ROR scoring by Relapse risk Prediction Models (Parker et al 2009) ...
## [DONE]
```

The predicted risk scores by Intrinsic signature can be computed using function `intrinsic.clone2affy()` on the Affy947 data. Similar to the PAM50, the ROR model (Parker et al. 2009) is applied to the intrinsic subtypes to caculate the risk scores. The conversion of the gene identifies from Stanford cDNA cloneIDs to Affymetrix U133a probeIDs is carried out by an internal function `clone2affyhgu133a()` with HG18 for probe annotation (option `UCSC_hg18 = T`).

```
out.intrinsic = intrinsic.clone2affy(x, UCSC_hg18 = T)

## #----------- Intrinsic signature (Perou et al 2000) ----------#
## >> Classify data from Affy U133a expresstion platform ...
## >> Extracting matched intrinsic gene expression matrix ...
## cross-platform mapping coverage: 410/549 = 75%
## >> Gene median centering ...
## >> Calibrating subtypes for single sample ...
## >> ROR scoring by Relapse risk Prediction Models (Parker et al 2009) ...
## [DONE]
```

The predicted risk scores by 70-gene signature can be computed using function `gene70.agilent25k2affy()` on the Affy947 data. The conversion of the gene identifies from Agilent 25k platform probeIDs to Affymetrix U133a probeIDs is carried out by an internal function `agilentHu25k2affyhgu133a()`.

```
out.70gene = gene70.agilent25k2affy(x, UCSC_hg18 = T)

## #----------- 70 gene signature/MammaPrint (vant Veer et. al 2002) ----------#
## >> INFO: Original platform: AgilentHu25k.
## >> INFO: Mapping (to EnsemblID) was done in advance and based sequency similarity.
## >> Classify data from affyU133a platform ...
## >> Extracting matched signature gene expression matrix ...
## cross-platform mapping coverage: 46/70=65.7%
## >> Calibrating 70gene centroid correlation for single sample ...
## [DONE]
```

The predicted risk scores by 76-gene signature can be computed using function `gene76.affy()` on the Affy947 data. This signature requires the information for ER status to be able to apply ER-specific classifier. Here, we use the ER status combined from the IHC status and ER probe expression value

(column *ER_IHC_expr*). Also, the 76-gene signature was originally applied to node-negative BC patients. The option `nodeNegOnly` can be turned off to applied to the whole cohort. We did not standardize the gene expression level on top of the normalized data (option `standardisation`=F). By default, the risk scores are computed using the *population based strategy* described in the study instead of the original cutoffs used in Wang et. al 2005.

```
col.ER = "ER_IHC_expr"
col.LN = "Node"
ER = pData(X)[, col.ER]
LN = pData(X)[, col.LN]
out.76gene = gene76.affy(x, ER = ER, LN = LN, standardisation = F, nodeNegOnly = F)

## #----------- 76 gene signature/Veridex (Wang et. al 2005)  ----------#
## ** Original platform:  Affy HG U133a & only on Node negative BCs
## >> Classify data from affyU133a platform ...
## >> not only compute for node negative patients
## >> Extracting matched signature gene expression matrix ...
##    coverage for genes in ER positive group: 60/60=100%
##    coverage for genes in ER negative group: 16/16=100%
## >> computing the raw relapse scores
## >> scaling prognositic group by population based classification
## >> scaliong risk group by cutoffs defined in origninal signature
```

The predicted risk scores by GGI can be computed using function `GGI.affy()` on the Affy947 data. This signature requires histological grade information to apply the classifier (column *Grade*).

```
col.HG = "Grade"
HG = pData(X)[, col.HG]
out.ggi = GGI.affy(x, HG = HG)

## #----------- GGI: Gemonic Grade Index (sotiriou et. al 2006) ----------#
## >> Classify data from affyU133a platform ...
##    coverage for G1 markers: 16/16=100%
##    coverage for G3 markers: 112/112=100%
```

The predicted risk scores by Wound Response signature can be computed using function `WR.clone2affy()` on the Affy947 data. The conversion of the gene identifies from Stanford cDNA cloneIDs to Affymetrix U133a probeIDs is carried out by an internal function `clone2affyhgu133a()`.

```
out.wr = WR.clone2affy(x, UCSC_hg18 = T)

## #----------- Wound-healing response signature (Chang et.al 2004 & 2005) ----------#
## >> Classify data from Affy U133a expresstion platform ...
## >> Extracting matched signature gene expression matrix ...
## cross-platform mapping coverage: 298/380=78%
## >> Gene median centering ...
## >> Calibrating CSR centroid correlation for single sample ...
## [DONE]
```

The predicted risk scores by Hypoxia signature can be computed using function `hypoxia.clone2affy()` on the Affy947 data. The conversion of the gene identifies from Stanford cDNA cloneIDs to Affymetrix U133a probeIDs is carried out by an internal function `clone2affyhgu133a()`.

```
out.hypoxia = hypoxia.clone2affy(x, UCSC_hg18 = T)

## #----------- Hypoxia signature (Chi et al 2006) ----------#
## >> Classify data from Affy U133a platform ...
## >> Extracting matched gene expression matrix ...
##    cross-platform mapping coverage: 117/253=46.2%
## Gene median centering ...
## >> Computing hypoxic score for single sample ...
## [DONE]
```

The predicted risk scores by the 21-gene-Recurrence-Score signature can be computed using function `OncotypeDX.symbol2affyhgu133a()` on the Affy947 data. The conversion of the gene identifies from gene symbol to Affymetrix U133a probeIDs is carried out by an internal function `symbol2affyhgu133a()`.

```
col.ER = "ER_IHC_expr"
col.LN = "Node"
ER = pData(X)[, col.ER]
LN = pData(X)[, col.LN]
out.RS = OncotypeDX.symbol2affyhgu133a(x, UCSC_hg18 = T)

##   mapping coverage for 16 cancer-related genes: 16/16=100%  mapping coverage for 5 refe
##   missing:
```

The predicted risk scores by the EndoPredict signature can be computed using function `EndoPredict.symbol2affyhgu133a()` on the Affy947 data.

```
out.EP = EndoPredict.symbol2affyhgu133a(x, UCSC_hg18 = T)

##   mapping coverage for 8 cancer-related genes: 8/8=100%  mapping coverage for 3 referen
##   missing:
```

The risk scores for individual gene signatures can then be extracted and store in R object `res1`.

```
tmp = pData(X)[, c("t_dmfs", "e_dmfs", "ER_IHC_expr", "Size", "Grade", "Node")]
tmp$Intrinsic_RORs = out.intrinsic$ROR_S
tmp$PAM50_RORs = out.pam50$ROR_S
tmp$gene70 = -out.70gene$corr
tmp$WR = out.wr$corr
tmp$gene76 = out.76gene$rawRelapseScore
tmp$GGI = out.ggi$rawGGI
tmp$RS = out.RS$RSu
tmp$Hypoxia = out.hypoxia$score
```

```
tmp$EP = out.EP$Su
# res1 = tmp
```

4 Analysis

4.1 Similarity on risk prediction across gene signatures

The correlation structure of risk prediction among gene signatures is evaluated using pearson correlation. (Figure 1A in Zhao et al).

```
GenesigOrder = c("Intrinsic_RORs", "PAM50_RORs", "gene70", "gene76", "GGI",
    "WR", "Hypoxia", "RS", "EP")
GenesigLabel = c("Intrinsic", "PAM50", "70-gene", "76-gene", "GGI", "WR", "Hypoxia",
    "RS", "EP")
res = res1
y = data.matrix(res[, GenesigOrder])
colnames(y) = GenesigLabel
colnames(y)[1:2] = c("Intrinsic-RORs", "PAM50-RORs")
corMatHeatmap(cor(y), displayVal.min = 0, live = F)
```

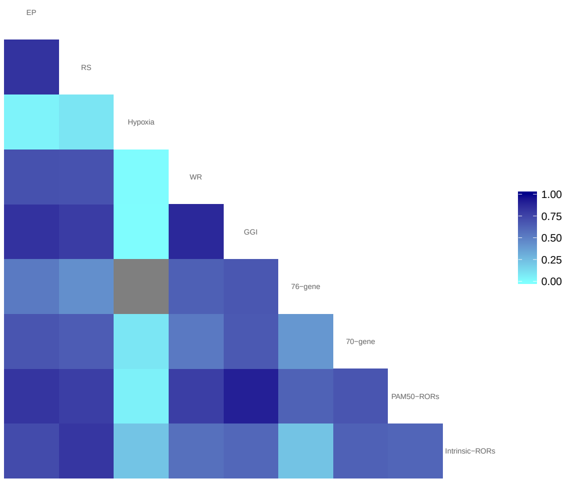

4.2 Time & ER dependency of gene signatures' prognostic effects

Additive regression model is used to study the profile of signature's prognositic power evolving along follow up time. Each gene signature, an additive regression model was fitted using mean-centered risk score as covariate. The time trend analsis is carried out on ER+ group (Figure S2B in Zhao et al.):

```r
res = res1
tmp = llply(GenesigOrder, function(x, y = subset(res, ER_IHC_expr == 1)) {
    y[, x] = y[, x] - mean(y[, x])  # mean center each covariates
    tmp = addreg(as.formula(paste("Surv(", para.t, ",", para.e, ") ~ ", x, sep = "")),
        data = y[y[, para.t] != 0, ])
    getdata_plot.addreg.plus(tmp)
})

## Remark: Stopped at time 293.8 because of too low rank.
##          (Last estimate at time 231.8)
## Remark: Stopped at time 293.8 because of too low rank.
##          (Last estimate at time 231.8)
## Remark: Stopped at time 231.8 because of too low rank.
##          (Last estimate at time 227.6)
## Remark: Stopped at time 293.8 because of too low rank.
##          (Last estimate at time 231.8)
## Remark: Stopped at time 293.8 because of too low rank.
##          (Last estimate at time 231.8)
## Remark: Stopped at time 293.8 because of too low rank.
##          (Last estimate at time 231.8)
## Remark: Stopped at time 231.8 because of too low rank.
##          (Last estimate at time 227.6)
## Remark: Stopped at time 293.8 because of too low rank.
##          (Last estimate at time 231.8)
## Remark: Stopped at time 293.8 because of too low rank.
##          (Last estimate at time 231.8)

names(tmp) = GenesigLabel
plot.addreg.plus(tmp, ncol.facet = 3) + theme(axis.text.x = element_text(v = 1,
    colour = "grey50", size = 8)) + theme(axis.text.y = element_text(h = 1,
    colour = "grey40", size = 6)) + theme(axis.ticks = element_line(colour = "grey75",
    size = 0.3)) + scale_x_continuous("Time to distant metastasis (year)", breaks = c(0,
    12 * 5, 12 * 10, 12 * 15, 12 * 20), labels = c(0, 5, 10, 15, 20))
```

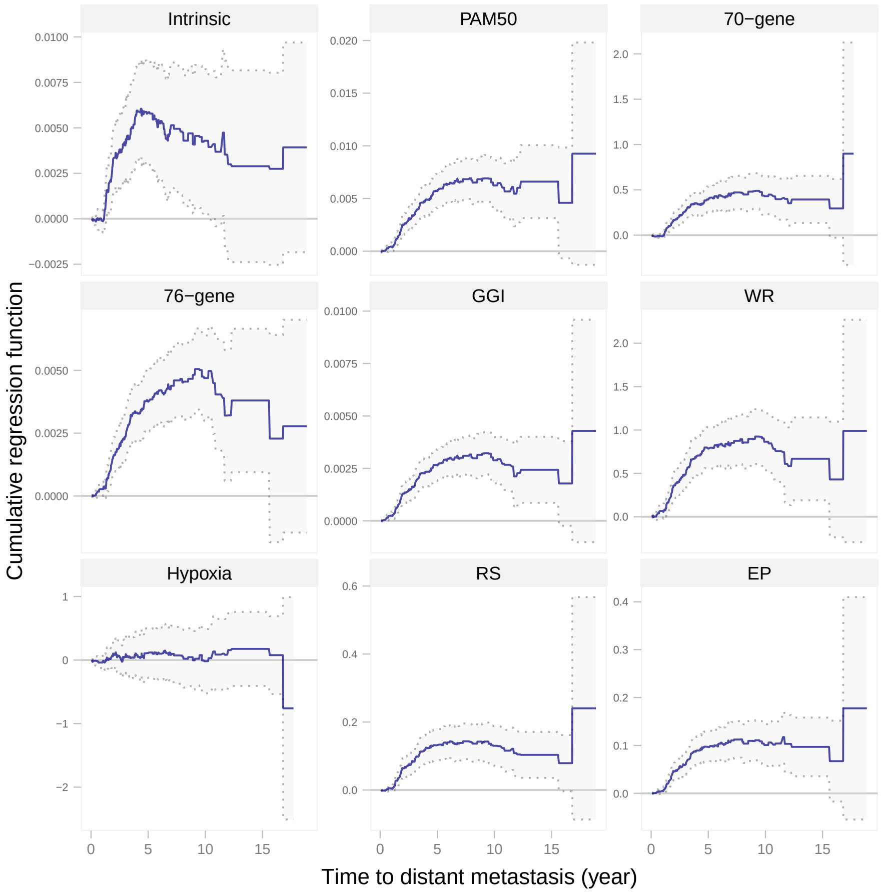

And the following figure shows the time trend analsis on ER- group (Figure S2C in Zhao et al.):

```
tmp = llply(GenesigOrder, function(x, y = subset(res, ER_IHC_expr == 0)) {
    y[, x] = y[, x] - mean(y[, x])  # centered by mean
    tmp = addreg(as.formula(paste("Surv(", para.t, ",", para.e, ") ~ ", x, sep = "")),
        data = y[y[, para.t] != 0, ])
    getdata_plot.addreg.plus(tmp)
})

## Remark: Stopped at time 205.2 because of too low rank.
##         (Last estimate at time 201.6)
## Remark: Stopped at time 205.2 because of too low rank.
##         (Last estimate at time 201.6)
## Remark: Stopped at time 205.2 because of too low rank.
##         (Last estimate at time 201.6)
## Remark: Stopped at time 205.2 because of too low rank.
##         (Last estimate at time 201.6)
## Remark: Stopped at time 205.2 because of too low rank.
##         (Last estimate at time 201.6)
## Remark: Stopped at time 205.2 because of too low rank.
```

```
##          (Last estimate at time 201.6)
## Remark: Stopped at time 205.2 because of too low rank.
##          (Last estimate at time 201.6)
## Remark: Stopped at time 205.2 because of too low rank.
##          (Last estimate at time 201.6)
## Remark: Stopped at time 205.2 because of too low rank.
##          (Last estimate at time 201.6)

names(tmp) = GenesigLabel
plot.addreg.plus(tmp, ncol.facet = 3) + theme(axis.text.x = element_text(v = 1,
    colour = "grey50", size = 8)) + theme(axis.text.y = element_text(h = 1,
    colour = "grey40", size = 6)) + theme(axis.ticks = element_line(colour = "grey75",
    size = 0.3)) + scale_x_continuous("Time to distant metastasis (year)", breaks = c(0,
    12 * 5, 12 * 10, 12 * 15, 12 * 20), labels = c(0, 5, 10, 15, 20))
```

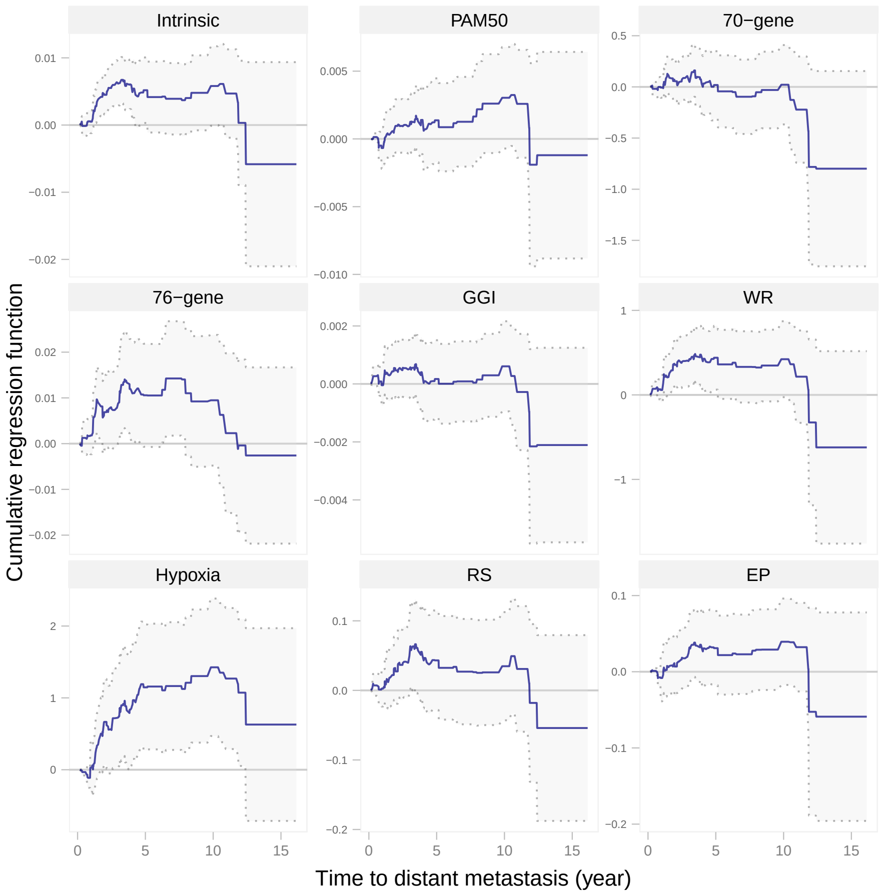

Based on the indications from the running risk profiles by the additive regression model, we devide the follow-up time intervals into: up to 5-year, 5-10 year, and beyond 10 year. Within each subinterval, a univariate Cox model per signature was fitted. The analysis is done for ER+ group and ER- group seperately. The following figure (Figure 2 in Zhao et al.) shows the estimated effect (standardized hazard ratios, with 95% confidence intervals) of gene signatures

for survival prediction within different time intervals for each of the ER groups.

```
paras = GenesigOrder
mat = res1
colnames(mat)[colnames(mat) %in% para.t] = "Time"
colnames(mat)[colnames(mat) %in% para.e] = "Status"

# 1.follow-up to 5-year (censor patients experiencing an event > 5 years at
# 5 year)
tmp.t = mat$Time
tmp.t[(mat$Status == 1) & (mat$Time > 5 * 12)] = 5 * 12
tmp.e = mat$Status
tmp.e[(mat$Status == 1) & (mat$Time > 5 * 12)] = 0
tmp = mat
tmp$Time = tmp.t
tmp$Status = tmp.e
colnames(tmp)[match(c("Time", "Status"), colnames(tmp))] = c(para.t, para.e)
mat1 = tmp
mat1.sd = mat1
mat1.sd[, paras] = apply(mat1.sd[, paras], 2, scale)

# 2. 5-10 year (excluded patients experiencing an event <= 5 years and
# censor those > 10 years at 10 year
tmp = subset(mat, !((Status == 1) & (Time <= 5 * 12)))
tmp.t = tmp$Time
tmp.t[(tmp$Status == 1) & (tmp$Time > 10 * 12)] = 10 * 12
tmp.e = tmp$Status
tmp.e[(tmp$Status == 1) & (tmp$Time > 10 * 12)] = 0
tmp$Time = tmp.t
tmp$Status = tmp.e
colnames(tmp)[match(c("Time", "Status"), colnames(tmp))] = c(para.t, para.e)
mat2 = tmp
mat2.sd = mat2
mat2.sd[, paras] = apply(mat2.sd[, paras], 2, scale)

# 3. beyond 10 year (excluded patients experiencing an event <= 10 years)
tmp = subset(mat, !((Status == 1) & (Time <= 10 * 12)))
colnames(tmp)[match(c("Time", "Status"), colnames(tmp))] = c(para.t, para.e)
mat3 = tmp
mat3.sd = mat3
mat3.sd[, paras] = apply(mat3.sd[, paras], 2, scale)

# plot the HR + CI in two ER groups: variables were scaled -----
mat_list_ER1 = list(`0~5` = scalesubdata(mat1, ER = 1), `5~10` = scalesubdata(mat2,
    ER = 1), `>10` = scalesubdata(mat3, ER = 1))
mat_list_ER0 = list(`0~5` = scalesubdata(mat1, ER = 0), `5~10` = scalesubdata(mat2,
    ER = 0), `>10` = scalesubdata(mat3, ER = 0))

strataval = c("ER+", "ER-")
```

```
d.l = list(mat_list_ER1, mat_list_ER0)
d = list()
d[[1]] = ldply(mat_list_ER1, function(x) summary_cox(x, xparas = paras, xpara.t = para.t,
    xpara.e = para.e))
d[[2]] = ldply(mat_list_ER0, function(x) summary_cox(x, xparas = paras, xpara.t = para.t,
    xpara.e = para.e))
names(d) = strataval
d = melt(d, id.vars = colnames(d[[1]]))
d$vars = factor(d$vars, levels = GenesigOrder, labels = GenesigLabel)
d$p.lab2 = cut(d$p, breaks = c(0, 0.05, 1), labels = c("p<=0.05", "p>0.05"))
d$.id = factor(d$.id, levels = c("0~5", "5~10", ">10"))
d$L1 = with(d, factor(L1, levels = c("ER+", "ER-")))

ytickval = c(seq(0.1, 0.5, 0.2), seq(0.5, 2, 0.5), seq(2, 4, 1))
ggplot(data = d, aes(x = .id, y = expcoef)) + geom_hline(yintercept = 1, size = 0.2,
    colour = "darkblue", alpha = I(1/2)) + geom_line(aes(colour = factor(L1),
    group = factor(L1)), alpha = I(1/2)) + geom_errorbar(aes(ymin = l, ymax = h,
    colour = factor(L1)), alpha = I(1/3), width = 0.3) + geom_point(aes(colour = factor(L1
    shape = p.lab2), fill = "white", alpha = 0.9, size = 1.4) + facet_grid(. ~
    vars) + labs(colour = "", linetype = "", x = "Follow-up time interval (year)",
    y = "Standardized hazard ratio") + myTheme.white.facet.axis() + scale_y_log10(breaks =
    labels = ytickval) + scale_shape_manual(name = "", values = c(19, 21), breaks = c("p<=
    "p>0.05"), labels = c(expression(p <= 0.05), expression(p > 0.05))) + theme(legend.pos
    legend.direction = "horizontal", legend.title.align = 0) + theme(axis.text.x = element
    colour = "grey50", size = 8, angle = 45))
```

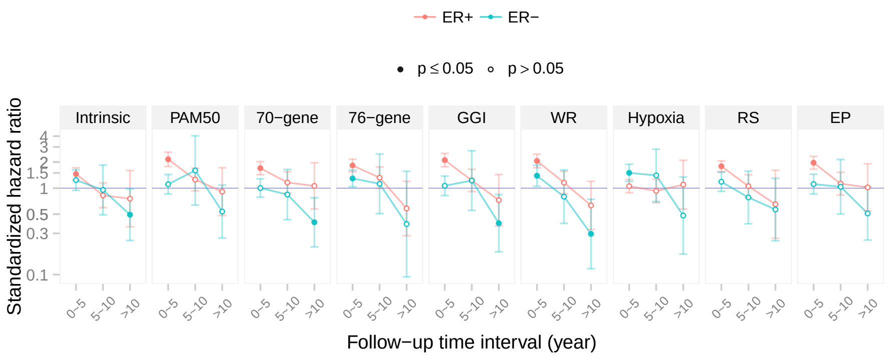

5 Data repository

All data used in this analysis is archived at `http://heim.ifi.uio.no/~bioinf/Projects/GeneSignatures/Zhao2014.htm`.

6 Contact Information

For questions or comments on the data contained within this analysis, the methods used or further help to reproduce the analyses please contact Xi Zhao

(xi.cameron@me.com).

7 Session Info

```
sessionInfo()

## R version 3.0.2 (2013-09-25)
## Platform: x86_64-apple-darwin10.8.0 (64-bit)
##
## locale:
## [1] en_US.UTF-8/en_US.UTF-8/en_US.UTF-8/C/en_US.UTF-8/en_US.UTF-8
##
## attached base packages:
##  [1] grid      splines   parallel  stats     graphics  grDevices utils
##  [8] datasets  methods   base
##
## other attached packages:
##  [1] e1071_1.6-1        class_7.3-9        biomaRt_2.16.0
##  [4] scales_0.2.3       Hmisc_3.13-0       Formula_1.1-1
##  [7] lattice_0.20-24    cluster_1.14.4     survival_2.37-4
## [10] reshape2_1.2.2     plyr_1.8           ggplot2_0.9.3.1
## [13] Biobase_2.20.1     BiocGenerics_0.6.0 knitr_1.5
##
## loaded via a namespace (and not attached):
##  [1] colorspace_1.2-4   dichromat_2.0-0    digest_0.6.4
##  [4] evaluate_0.5.1     formatR_0.10       gtable_0.1.2
##  [7] highr_0.3          labeling_0.2       MASS_7.3-29
## [10] munsell_0.4.2      proto_0.3-10       RColorBrewer_1.0-5
## [13] RCurl_1.95-4.1     stringr_0.6.2      tools_3.0.2
## [16] XML_3.95-0.2
```
