# Supplementary material for: Systematic assessment of prognostic gene signatures for breast cancer shows distinct influence of time and ER status
Source: BMC Cancer. 2014 Mar 19;14:211. doi: 10.1186/1471-2407-14-211 (PMC4000128; doi:10.1186/1471-2407-14-211)
Supplement: Additional file 5 — Supplementary figures. [file 1471-2407-14-211-S5.pdf]

Supplementary Figure Legends

Figure S1. Comparison of subtype calls between "Intrinsic subtype" and "PAM50". (A) The distributions of the PAM50 calls within individual Intrinsic subtypes. The classification agreements for individual subtypes (in percentage) between Intrinsic and PAM50 were: 86.5% for Basal-like, 74.4 % for HER2-enriched, 52.4% for LumA, 58.2% for LumB and 60.1% for Normal-like. Cohen's kappa coefficient κ on discrete subtype calls was used to measure the overall agreement on the subtype assignments. **(B)** Correlation for Intrinsic signature and PAM50. Each dot represents the centroid correlation score for one sample from Intrinsic (X-axis) and PAM50 (Y-axis). Pearson correlation coefficient on subtype-centroid correlations was used to measure the stability of a specific subtype. **(C)** A distance measurement *Delta* (D) per sample was defined to gauge the *discriminant ability* for individual subtypes in a signature and the *degree of inconsistency* for the same subtype in another signature. The D score was essentially the distance between the two subtype assignments on a sample, and it was computed as the difference of the corresponding centroid correlations. For a tumor per signature, the distance between the top two potential subtype assignments was measured: the larger the D, the more distinguishable the assigned subtype on the sample by the signature. In the case of inconsistent assignments for the same sample across the signatures, the distance between the previous assignment and the new assignment was measured to quantify the degree of the inconsistency. *Upper panel*: Samples were ranked by their distance measurements from intrinsic signature. *Lower panel*: X-axis indicates ranks from the intrinsic signature for individual samples. Y-axis indicates the D scores from PAM50 for the corresponding samples. Basal subtype was fairly robust in the two signatures,

as only the samples with borderline calls in the intrinsic signature had inconsistent calls in PAM50.

Figure S2. Evaluation of time- & ER-dependency in predicting Distant Metastasis Free Survival by gene signatures on the complete dataset (n=912). (A-B) Test for non-proportional hazard assumption for DMFS prediction by gene signatures in ER-positive group (A; n=692) and ER-negative group (B; n=220) separately. A univariate Cox model was fitted for each signature, and the scaled Schoenfeld residuals were tested against transformed time (Kaplan-Meier estimates). Residuals distributed randomly around 0 would satisfy the PH assumption. **(C-D)** Cumulative regression plots of the estimate along with 95 percent pointwise confidence interval from a univariate additive regression model for individual gene signatures. The curve in each plot reflects the cumulative effect of a signature covariate on survival over time. The slope of the curve reflects the effects of the signature on survival at any given time. Hence, a time-independent effect should result in a curve with a constant slope. **(C)** In ER-positive group (n=692). **(D)** In ER-negative group (n=220).

Figure S3. Evaluation of time- & ER-dependency in predicting Distant Metastasis Free Survival by gene signatures on systemically untreated patients (A-C) and systemically treated patients (D-F), separately. (A-B; D-E) Cumulative regression plots of the estimate along with 95 percent pointwise confidence interval from a univariate additive regression model for each individual gene signature. **(A)** In ER-positive & systemically untreated group (n=274). **(B)** In ER-negative & systemically untreated group (n=121). **(D)** In ER-positive & systemically treated

group (n=373). **(E)** In ER-negative & systemically treated group (n=63). **(C; F)** Estimated effect (standardized hazard ratios, e^{β}, with 95% confidence intervals) of gene signatures for survival prediction within different time intervals stratified by ER status. The X-axis indicates the follow-up time intervals: up to 5-year, 5-10 year, and beyond 10 year. Within each subinterval, a univariate Cox model per signature was fitted. The Y-axis indicates the estimated hazard ratios (HR) on a logarithmic scale corresponding to a 1 standard deviation increase in the signature. The null, HR=1, is indicated by the blue line. Solid dots indicate HRs significantly different from 1 (P<0·05). ER+ is denoted as red and ER– is denoted as blue**.**

Figure S4. Analysis on additional parameters for breast cancer prognostication in the studied cohort. (A-B) Known prognostic parameters predict Distant Metastasis Free Survival. **(A)** Kaplan Meier plot up to 15 years for: Node, Tumor size and Histological grade, respectively. **(B)** Kaplan Meier plot up to 15 years for Histological grade in ER+ subgroup and ER- subgroup, respectively. **(C)** *TP53* mutated tumors associated with elevated hypoxic score in ER-negative group. Analysis is based on the 335 breast cancers that had been characterized for the presence or absence of *TP53* mutations. One-tailed t-test was performed to assess the significance of increases in Hypoxia score for samples with *TP53* mutated over the wild-type ones. **(D)** Univariate analysis on gene signatures with G1, G2, G3 separately in ER+ group. Risk scores for individual gene signatures were standardized, and prognostic effects of signatures were compared using Hazard Ratio from univariate Cox model: Risk = *Signature* in ER+ group (n= 715; panel *ER+*), G1 samples of the ER+ group, (n= 153; panel *ER+/G1*), G2 samples of the ER+ group, (n= 311; panel *ER+/G2*) and G3 samples of the ER+ group, (n=149; panel *ER+/G3*).

X-axis indicates the Standardized Hazard Ratio. **(E)** Univariate analysis on gene signatures with T1, T2, T3 separately in ER+ group. Risk scores for individual gene signatures were standardized, and prognostic effects of signatures were compared using Hazard Ratio from univariate Cox model: Risk = *Signature* in ER+ group (n= 715; panel *ER+*), T1 samples of the ER+ group, (n= 349; panel *ER+/T1*), T2 samples of the ER+ group, (n= 335; panel *ER+/T2*) and T3 samples of the ER+ group, (n=26; column *ER+/T3*). X-axis indicates the Standardized Hazard Ratio.

Figure S5. (A) Evaluation of time- & HER2-dependency in predicting Disease-specific Survival by gene signatures on the complete dataset (n=912). Estimated effect (standardized hazard ratios, e^{β}, with 95% confidence intervals) of gene signatures for survival prediction within different time intervals stratified by HER2 status. The X-axis indicates the follow-up time intervals: up to 5 years, 5-10 years, and beyond 10 years. Within each subinterval, a univariate Cox model per signature was fitted. The Y-axis indicates the estimated hazard ratios (HR) on a logarithmic scale corresponding to a 1 standard deviation increase in the signature. The null, HR=1, is indicated by the blue line. Solid dots indicate HRs significantly different from 1 ($P<0{\cdot}05$). HER2+ (n=114) is denoted as red and HER2– (n= 798) is denoted as blue**. (B) Evaluation of time- & HER2 / ER dependency in predicting Disease-specific Survival by gene signatures on the complete dataset (n=912).**

Figure S6. Evaluation of time- & ER-dependency in predicting Disease-specific Survival by gene signatures on METABRIC discovery set (n=996). (A-B) Cumulative regression plots of the estimate along with 95 percent pointwise confidence interval from a univariate additive regression model for each individual

gene signature. **(A)** In ER-positive group of the complete METABRIC discovery set (n=801). **(B)** In ER-negative group of the complete METABRIC discovery set (n=195). **(C-E)** Estimated effect (standardized hazard ratios, e^{β}, with 95% confidence intervals) of gene signatures for survival prediction within different time intervals stratified by ER status. The X-axis indicates the follow-up time intervals: up to 5-year, 5-10 year, and beyond 10 year. Within each subinterval, a univariate Cox model per signature was fitted. The Y-axis indicates the estimated hazard ratios (HR) on a logarithmic scale corresponding to a 1 standard deviation increase in the signature. The null, HR=1, is indicated by the blue line. Solid dots indicate HRs significantly different from 1 ($P<0{\cdot}05$). ER+ is denoted as red and ER– is denoted as blue**.** **(C)** On the complete METABRIC discovery set. **(D)** On the systemically untreated patients and **(E)** On the systemically treated patients**.**

Figure S1

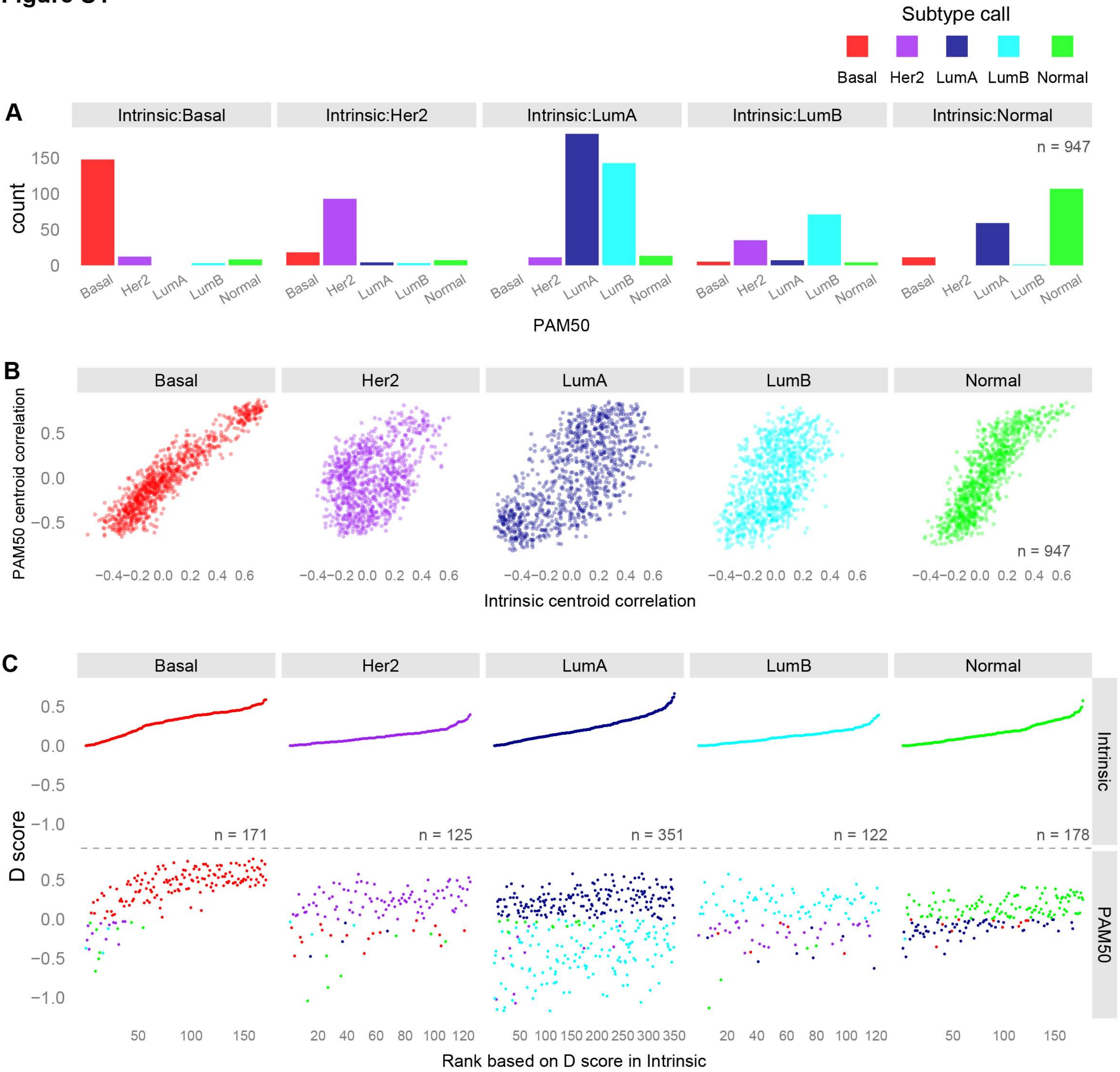

Subtype call
Basal
Her2
LumA
LumB
Normal
A
Intrinsic:Basal
Intrinsic:Her2
Intrinsic:LumA
Intrinsic:LumB
Intrinsic:Normal
n = 947
count
PAM50
B
PAM50 centroid correlation
Intrinsic centroid correlation
n = 947
C
Intrinsic
PAM50
D score
n = 171
n = 125
n = 351
n = 122
n = 178
Rank based on D score in Intrinsic

Figure S2

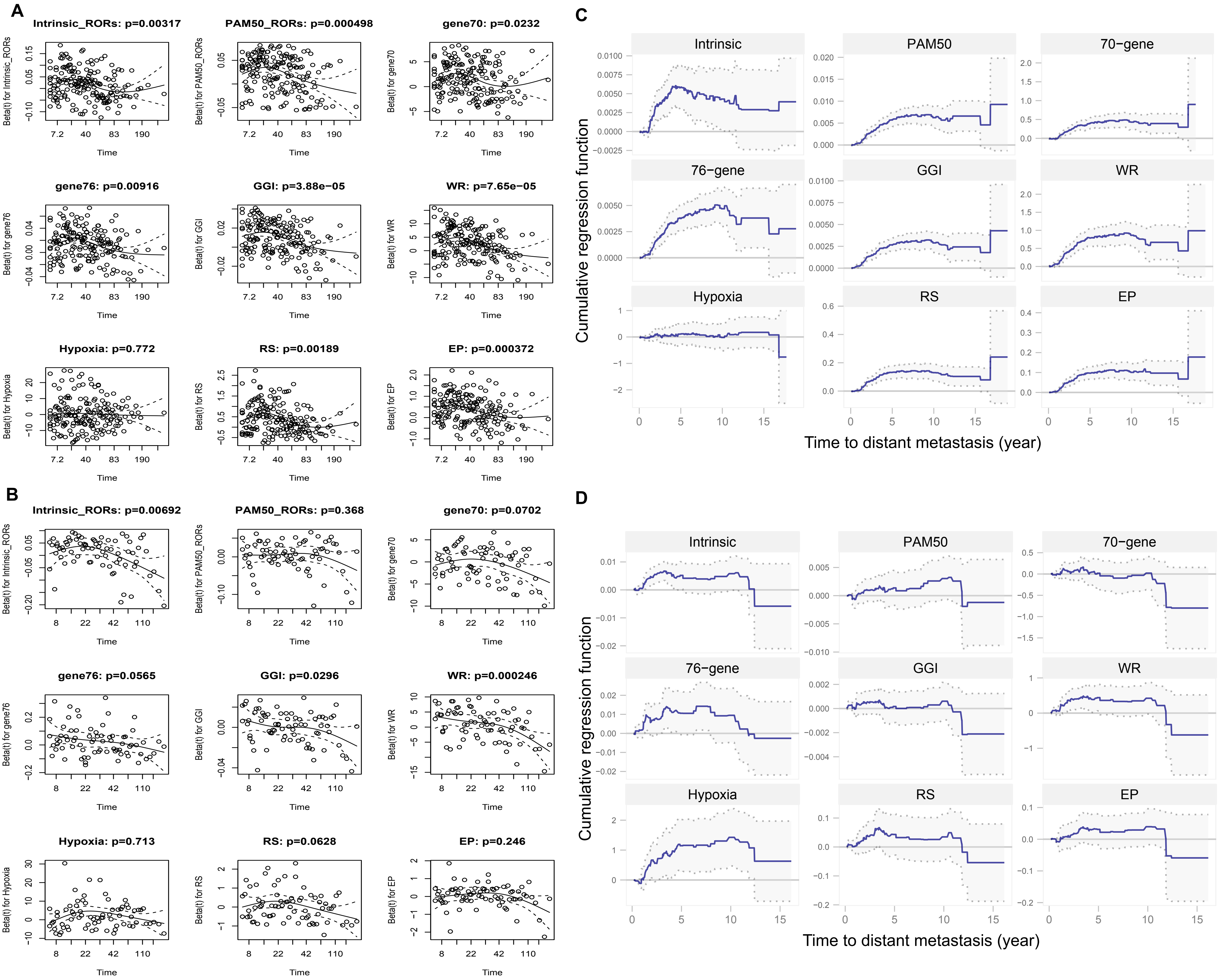
A
Intrinsic_RORs: p=0.00317
PAM50_RORs: p=0.000498
gene70: p=0.0232
gene76: p=0.00916
GGI: p=3.88e−05
WR: p=7.65e−05
Hypoxia: p=0.772
RS: p=0.00189
EP: p=0.000372
Beta(t) for Intrinsic_RORs
Beta(t) for PAM50_RORs
Beta(t) for gene70
Beta(t) for gene76
Beta(t) for GGI
Beta(t) for WR
Beta(t) for Hypoxia
Beta(t) for RS
Beta(t) for EP
Time
B
Intrinsic_RORs: p=0.00692
PAM50_RORs: p=0.368
gene70: p=0.0702
gene76: p=0.0565
GGI: p=0.0296
WR: p=0.000246
Hypoxia: p=0.713
RS: p=0.0628
EP: p=0.246
C
Intrinsic
PAM50
70−gene
76−gene
GGI
WR
Hypoxia
RS
EP
Cumulative regression function
Time to distant metastasis (year)
D
Intrinsic
PAM50
70−gene
76−gene
GGI
WR
Hypoxia
RS
EP
Cumulative regression function
Time to distant metastasis (year)

Figure S3

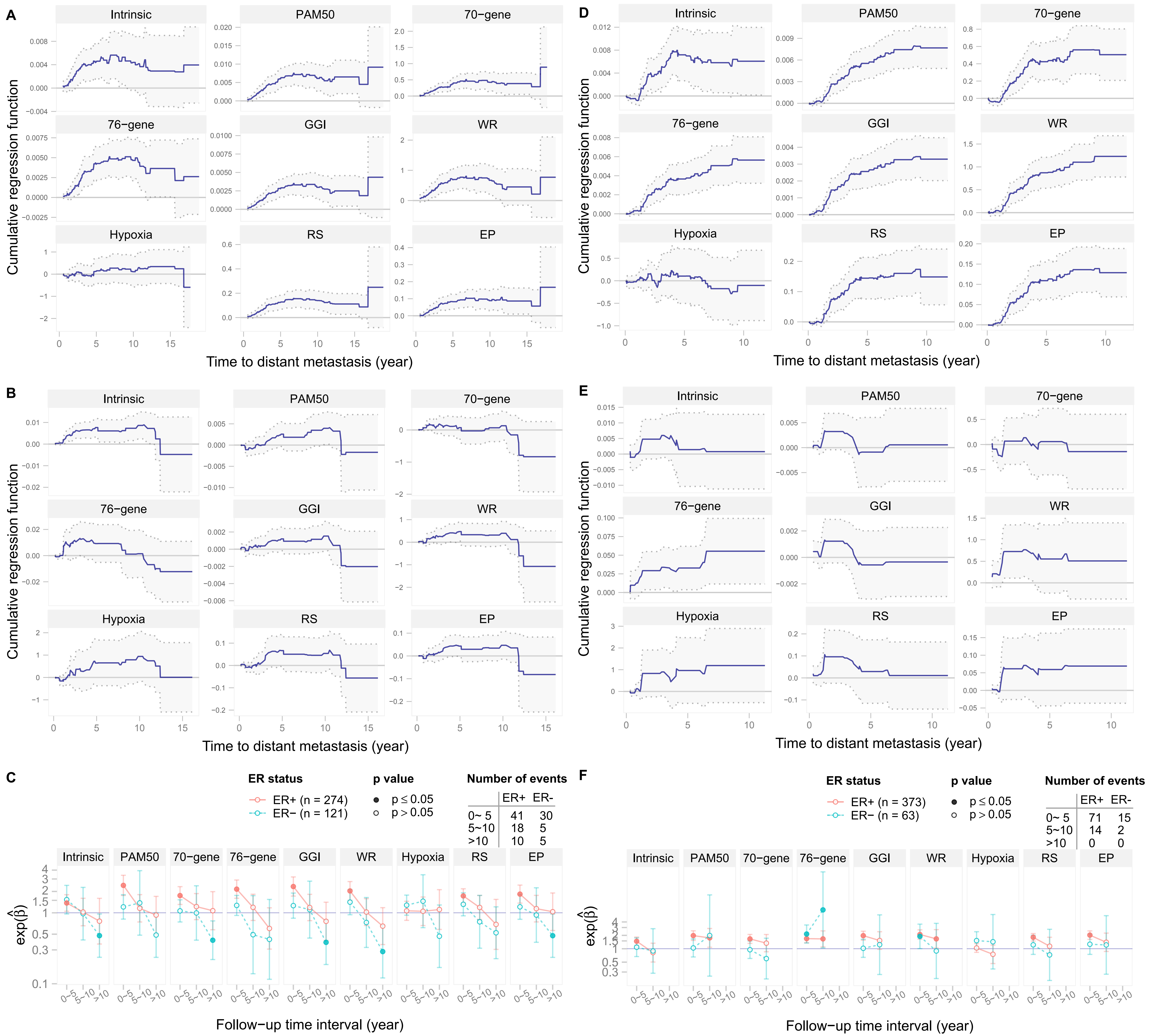
A
Intrinsic
PAM50
70−gene
76−gene
GGI
WR
Hypoxia
RS
EP
Cumulative regression function
Time to distant metastasis (year)
B
Intrinsic
PAM50
70−gene
76−gene
GGI
WR
Hypoxia
RS
EP
Cumulative regression function
Time to distant metastasis (year)
C
ER status
ER+ (n = 274)
ER− (n = 121)
p value
p ≤ 0.05
p > 0.05
Number of events
ER+ ER-
0~ 5 41 30
5~10 18 5
>10 10 5
Intrinsic
PAM50
70−gene
76−gene
GGI
WR
Hypoxia
RS
EP
exp(β̂)
0~5
5~10
>10
Follow−up time interval (year)
D
Intrinsic
PAM50
70−gene
76−gene
GGI
WR
Hypoxia
RS
EP
Cumulative regression function
Time to distant metastasis (year)
E
Intrinsic
PAM50
70−gene
76−gene
GGI
WR
Hypoxia
RS
EP
Cumulative regression function
Time to distant metastasis (year)
F
ER status
ER+ (n = 373)
ER− (n = 63)
p value
p ≤ 0.05
p > 0.05
Number of events
ER+ ER-
0~ 5 71 15
5~10 14 2
>10 0 0
Intrinsic
PAM50
70−gene
76−gene
GGI
WR
Hypoxia
RS
EP
exp(β̂)
0~5
5~10
>10
Follow−up time interval (year)

Figure S4

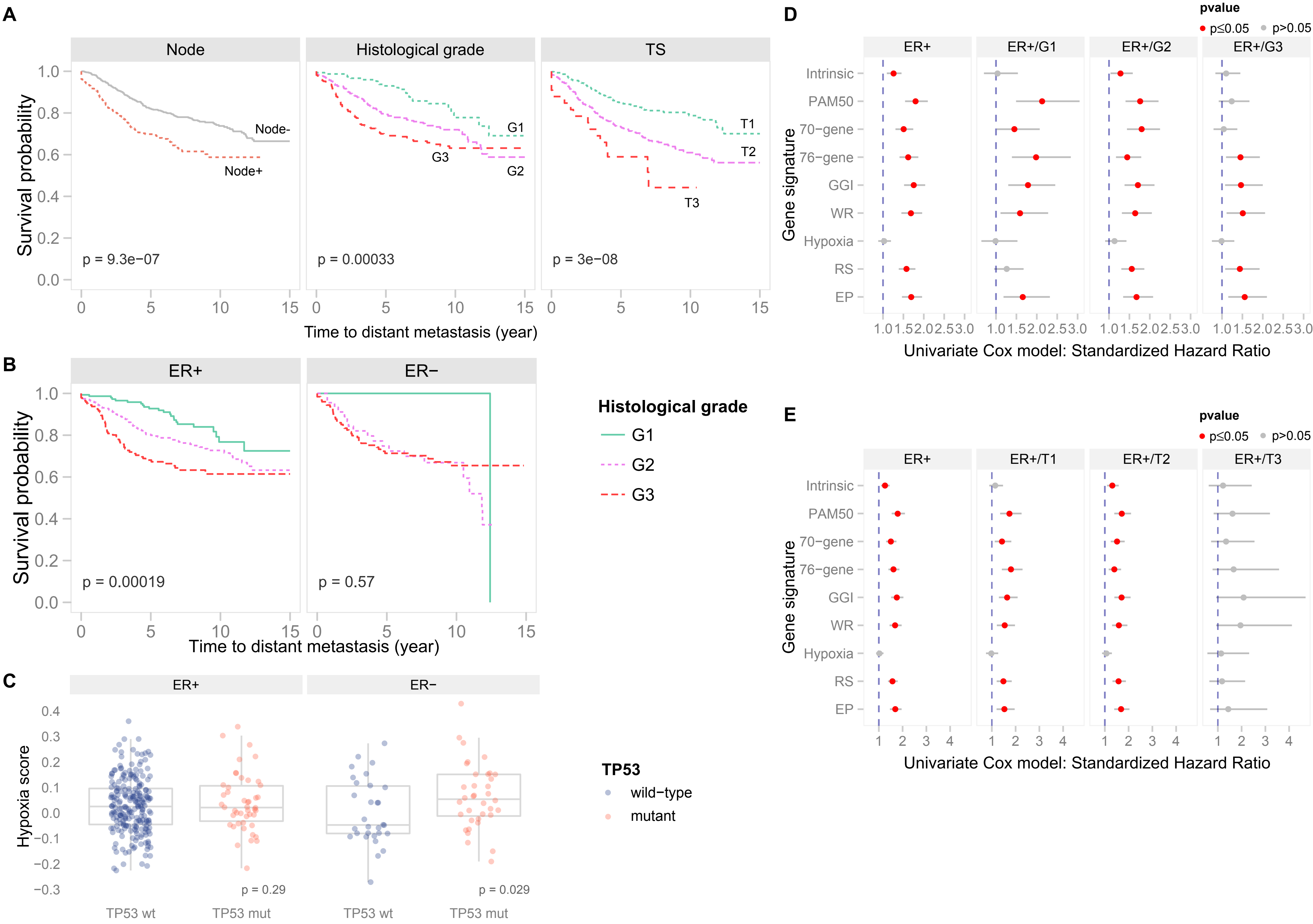

A
Node
Histological grade
TS
Survival probability
1.0
0.8
0.6
0.4
0.2
0.0
Node-
Node+
p = 9.3e−07
G1
G3
G2
p = 0.00033
T1
T2
T3
p = 3e−08
0
5
10
15
Time to distant metastasis (year)
B
ER+
ER−
p = 0.00019
p = 0.57
Histological grade
G1
G2
G3
C
Hypoxia score
TP53 wt
TP53 mut
p = 0.29
p = 0.029
TP53
wild-type
mutant
D
pvalue
p≤0.05
p>0.05
ER+/G1
ER+/G2
ER+/G3
Gene signature
Intrinsic
PAM50
70−gene
76−gene
GGI
WR
Hypoxia
RS
EP
Univariate Cox model: Standardized Hazard Ratio
E
ER+/T1
ER+/T2
ER+/T3

Figure S5

A

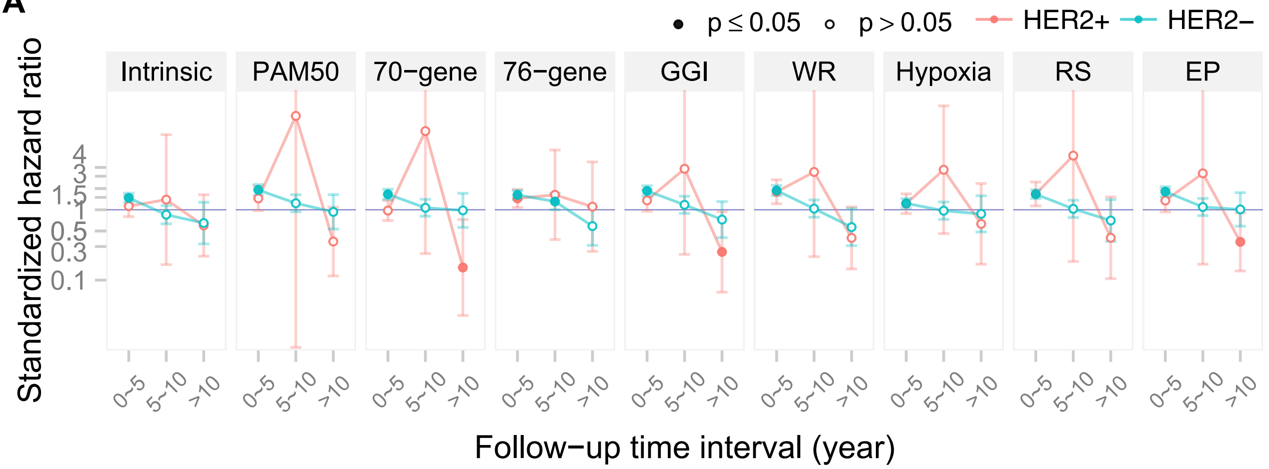

p ≤ 0.05
p > 0.05
HER2+
HER2−
Intrinsic
PAM50
70−gene
76−gene
GGI
WR
Hypoxia
RS
EP
Standardized hazard ratio
4
3
1.5
1
0.5
0.3
0.1
0~5
5~10
>10
Follow−up time interval (year)

B

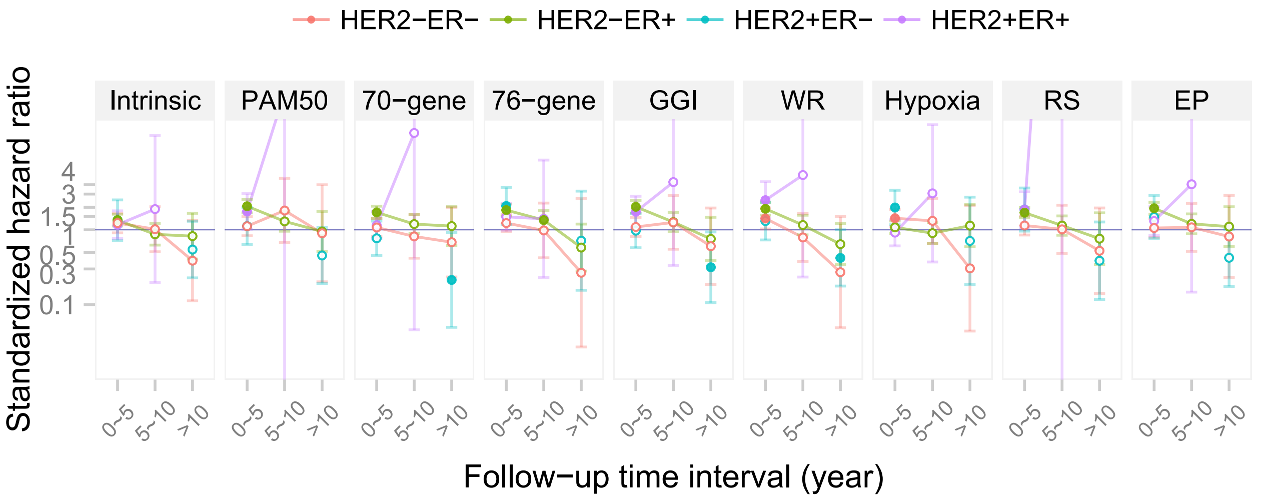

HER2−ER−
HER2−ER+
HER2+ER−
HER2+ER+
Intrinsic
PAM50
70−gene
76−gene
GGI
WR
Hypoxia
RS
EP
Standardized hazard ratio
4
3
1.5
1
0.5
0.3
0.1
0~5
5~10
>10
Follow−up time interval (year)

Figure S6

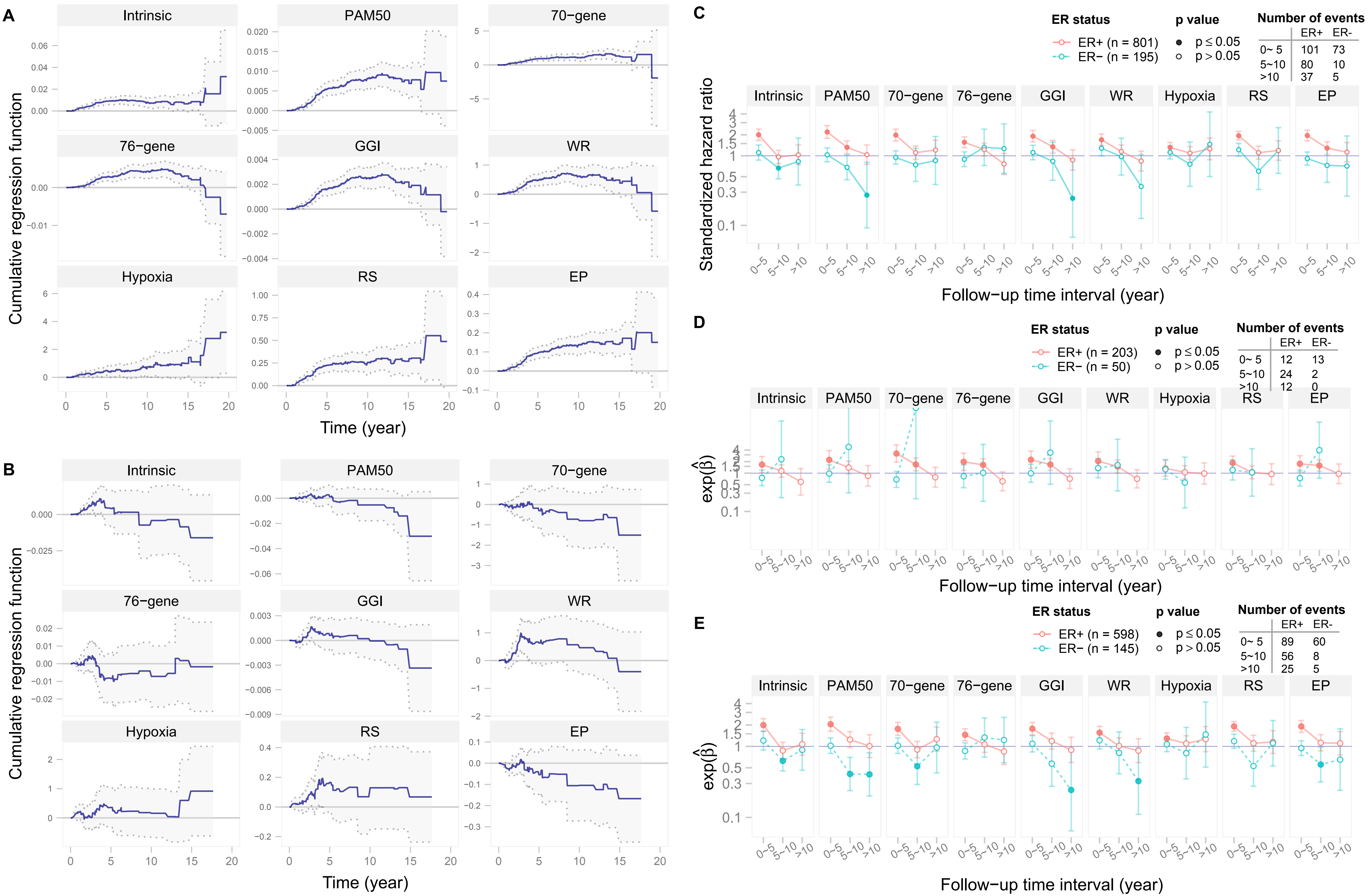

A
Intrinsic
PAM50
70−gene
76−gene
GGI
WR
Hypoxia
RS
EP
Cumulative regression function
Time (year)
B
Intrinsic
PAM50
70−gene
76−gene
GGI
WR
Hypoxia
RS
EP
Cumulative regression function
Time (year)
C
ER status
ER+ (n = 801)
ER− (n = 195)
p value
p ≤ 0.05
p > 0.05
Number of events
ER+ ER-
0~ 5 101 73
5~10 80 10
>10 37 5
Standardized hazard ratio
Follow−up time interval (year)
D
ER status
ER+ (n = 203)
ER− (n = 50)
p value
p ≤ 0.05
p > 0.05
Number of events
ER+ ER-
0~ 5 12 13
5~10 24 2
>10 12 0
Follow−up time interval (year)
E
ER status
ER+ (n = 598)
ER− (n = 145)
p value
p ≤ 0.05
p > 0.05
Number of events
ER+ ER-
0~ 5 89 60
5~10 56 8
>10 25 5
Follow−up time interval (year)
